# Genome-Wide Identification and Tissue-Specific Expression Analysis of UDP-Glycosyltransferases Genes Confirm Their Abundance in *Cicer arietinum* (Chickpea) Genome

**DOI:** 10.1371/journal.pone.0109715

**Published:** 2014-10-07

**Authors:** Ranu Sharma, Vimal Rawat, C. G. Suresh

**Affiliations:** 1 Division of Biochemical Sciences, CSIR-National Chemical Laboratory, Pune, Maharashtra, India; 2 Department of Plant Developmental Biology, Max Planck Institute for Plant Breeding Research, Cologne, Germany; ISA, Portugal

## Abstract

UDP-glycosyltransferases (EC 2.4.1.x; UGTs) are enzymes coded by an important gene family of higher plants. They are involved in the modification of secondary metabolites, phytohormones, and xenobiotics by transfer of sugar moieties from an activated nucleotide molecule to a wide range of acceptors. This modification regulates various functions like detoxification of xenobiotics, hormone homeostasis, and biosynthesis of secondary metabolites. Here, we describe the identification of 96 *UGT* genes in *Cicer arietinum* (*CaUGT*) and report their tissue-specific differential expression based on publically available RNA-seq and expressed sequence tag data. This analysis has established medium to high expression of 84 *CaUGTs* and low expression of 12 *CaUGTs*. We identified several closely related orthologs of *Ca*UGTs in other genomes and compared their exon-intron arrangement. An attempt was made to assign functional specificity to chickpea UGTs by comparing substrate binding sites with experimentally determined specificity. These findings will assist in precise selection of candidate genes for various applications and understanding functional genomics of chickpea.

## Introduction

*Cicer arietinum*, commonly known as chickpea belongs to the plant family *Fabaceae*. It is one of the ancient and second most widely grown legumes in the world (FAO, 2008) [1]. Owing to its capacity for symbiotic nitrogen fixation, chickpea seeds are a primary source of human dietary protein. Chickpea is free from cholesterol and a good source of vitamins, minerals and fibers [2]. It has carotenoids like β-carotene, lutein, zeaxanthin, β-cryptoxanthin, lycopene and α-carotene. Chickpea contains phenolic compounds like isoflavones, biochanin A, formononetin, daidzein, genistein, matairesinol and secoisolariciresinol [2]. Research has shown that the consumption of chickpea seeds reduces the cholesterol level in blood [3]. Various bioactive compounds in plants have several economic and health benefits, therefore it will be important to study the genes involved in their biosynthesis.

In plants glycosylation of terpenoids, phenylpropanoids, cyanogenic glucosides and glucosinolates that alter their activity, sub-cellular location and modulates chemical properties like stability and solubility [4]. Glycosylation is catalyzed by a class of enzymes known as glycosyltransferases (EC 2.4.x.y) which belong to the transferase family and present in prokaryotes as well as eukaryotes. These enzymes are classified into 96 families in the CAZy database according to their amino acid sequence similarity [5], [6]. Out of these 96 GT families, the largest number belongs to family 1 involved in the glycosylation of secondary metabolites like hormones, flavonoids, pesticides and herbicides [6].

The UGTs transfer the glycosyl group (glucose, galactose, rhamnose, xylose etc) from an activated nucleoside diphosphate sugar donor (UDP-sugar) to a wide range of sugar or non-sugar acceptors as mentioned above with a direct displacement S_N_^2^-like mechanism [7], [8]. At the N-terminal domain (NTD) of UGTs, a conserved histidine residue, present close to both the bound sugar donor and acceptor molecules, plays the crucial role of catalytic base by interacting with the protonating group (OH, NH etc) of the acceptor and helps in its deprotonation. The protonated histidine in turn is stabilized by a conserved proximate aspartic acid in the structure. After deprotonation, the acceptor forms a nucleophilic oxyanion center which attacks the C1 carbon atom of the sugar donor and forms β-glycosidic linked product accompanied with the displacement of UDP moiety [8]. However, an alternative catalytic mechanism has been proposed in UGT of *G. max*, which is devoid of the catalytic histidine [9]. A conserved signature motif, known as Putative Secondary Plant Glycosyltransferase [8] or Plant Secondary Product Glycosyltransferase [9] (PSPG) motif of 44 amino acid length, present at the C-terminal domain of plant UGTs is involved in the binding of the nucleotide sugar donor substrate whereas the highly variable NTD accommodates a wide array of acceptor substrates (Figure S1) [10].

In this study, we have identified 96 *UGT* genes from chickpea using bioinformatics approaches. Based on genome similarity their close orthologs were identified in four dicot plants such as *Medicago truncatula*, *Glycine max*, *Vigna angularis*, and *Lotus japonicus*. Nine sequentially diverged UGTs were identified in chickpea which indicated their diversification from other four dicot genomes considered in this study. Arrangement and location of UGT genes on genome/chromosomes was analyzed and their exon-intron architecture was compared. 74 of the 96 chickpea UGTs could be functionally annotated by comparison with experimentally characterized and functionally annotated other plant UGT enzymes. RNA-seq data and expressed sequence tag (EST) libraries available at NCBI were searched for the expression patterns which indicated their differential expression in various chickpea tissues.

## Materials and Methods

### Identification of *Ca*UGTs

Draft genome of *C. arietinum* was downloaded from Legume information system (http://cicar.comparative-legumes.org/). Estimated genome size of *C. arietinum* was around 740 Mb. Draft genome assembly of *C. arietinum* consists of 28,269 gene models and 7,163 scaffolds covering 544.73 Mb (over 70% of estimated genome size) [11]. *Ca*UGTs were identified by following three methodologies i.e. Blastp, Position-Specific Weight Matrix (PSWM) guided search and hidden Markov model-profile (HMM-profile) search, respectively. Predicted proteome, which consisted of 28,269 gene models, of chickpea was taken as a dataset to carry out Basic Local Alignment Search (stand-alone blastp 2.2.22) [12] by taking conserved PSPG motif of UGT from *Vitis vinifera* (PDB-2C1Z) as a query which is the signature pattern for UGTs using Expectation value (E-value) cut off of 1. Identification of superfamily to which the predicted UGTs belong was carried out by using SUPERFAMILY server [13].

To further confirm the above results, a dataset of 89 protein sequences of UGTs from various plant sources was composed (Table S1). These sequences were used to de novo find the conserved motif of UGTs. Multiple EM for Motif Elicitation v4.9.0 suite (MEME) [14], [15] with zoops (zero or one occurrence) was used for searching conserved motif (E-value 2.5*10^−2741^). Accurate length of the motif was confirmed by considering bit score and relative entropy. A PSPG motif of these sequences was then used to create PSWM. This PSWM was used to screen the *Ca*UGTs using Motif Alignment & Search Tool v4.9.0 (MAST) of MEME suite [16]. All previously identified UGTs were also confirmed with this alternative approach (with E-value below 9.9*10^−09^).

Predicted proteome of chickpea was searched for the presence of UGTs by screening using HMM-profiles of Pfam 27.0 (Pfam family: PF00201.13) [17] with the help of HMMER 3.0. [18] (http://hmmer.org/) selecting E-value cut off of 1. The identified *UGT* genes and the corresponding protein sequences were used in further analysis.

### Phylogenetic and molecular evolutionary analysis

Dendrogram was drawn for 96 *Ca*UGTs in PHYML [19] to study their evolutionary relations. Amino acid sequences were given as input in phylip format keeping LG (Le and Gascuel) substitution model and proportion of invariable sites and number of substitution rate categories as 0 and 4. Nearest Neighbor Interchanges (NNI) algorithm was utilized in order to improve a reasonable starting tree topology. The fast likelihood-based method selected in order to generate the dendrogram was approximate LRT (aLRT) method [20].

### Functional specificity of chickpea UGTs

In order to identify functional specificity of *Ca*UGTs, 38 experimentally characterized UGT proteins with known substrate specificity from 21 plant species were retrieved from Swiss-Prot database (Table S2) [21]–[35]. These 38 UGTs have diverse acceptor specificity towards molecules like zeatin, abscisate, anthocyanidins, flavonols, hydroquinone and many more. A phylogenetic tree for the 96 *Ca*UGTs was generated combined with the above 38 other plant UGTs to analyze the clustering pattern by keeping the parameters same as those previously used in PHYML. The clustered UGTs were further analyzed for similarities of the eight regions of NTD exposed to substrate binding pocket.

### Molecular modeling of chickpea UGTs

A search using Basic Local Alignment Search Tool (BLAST) algorithm [36] was carried out against the Protein Data Bank (PDB) [37] to identify the high resolution crystal structures of homologous proteins. The sequence identity and E-value cut off were set to ≥30% and 1. Homology modeling of UGT protein sequences was performed using the Composite/Chimeric model type of Prime 3.1 [38], by taking crystal structures homologous to the target proteins as templates, in order to analyze their structural features, binding mode and affinity with the substrates. The stereochemical quality of the three-dimensional models generated was evaluated using PROCHECK [39], Verify3D [40], ERRAT [41], and ProSA [42]. Subsequently, the initial models were refined by employing impref minimization of protein preparation wizard [38], [43] and Impact 5.8 [38] minimization. These energy minimized structures were further used for the binding studies in Glide 5.8 [44] with their substrates generated using 2-D sketcher utility in Maestro 9.3.

### Detection of *Ca*UGTs orthologs in dicots

Orthologs of predicted *Ca*UGTs were searched in four dicot plant genomes of *M. truncatula*, *G. max*, *V. angularis*, and *L. japonicus* using Blast2Go [45] tool keeping E-value cut off 0.001 and sequence similarity ≥80%. These dicots were selected for analysis based on their reported chickpea homologous genomes [11].

### Analysis of intron gain/loss events

Introns in *CaUGT* genes were explored to identify characteristic features such as length, number, phase, and location in the genome. The three intron phases were assigned as 0 for introns between two codons, 1 for those between first and second base of codon and 2 for introns inserted between second and third base. The exon-intron architecture and their phases were obtained using the online Gene Structure Display Server (GSDS; http://gsds.cbi.pku.edu.cn) extracting both coding and genomic sequences [46].

### Gene expression analysis using RNA-seq & EST data

#### RNA-seq data

RNA-seq raw read data was downloaded from Sequence Read Archive (SRA) (http://www.ncbi.nlm.nih.gov/sra), for 5 different tissues from ICC4598 chickpea genotype namely, germinating seed (G. seed) (GSM1047862), young leaves (GSM1047863), shoot apical meristem (SAM) (GSM1047864), flower bud (GSM1047865, GSM1047866, GSM1047867, GSM1047868) and flower (GSM1047869, GSM1047870, GSM1047871, GSM1047872) [47]. Reads were mapped to genomic sequence of *C. arietinum* with spliced read mapper, TopHat [48]. Cufflinks tool [49] was used to estimate abundance of reads mapped to genes body and calculated Fragments Per Kilobase of transcript per Million (FPKM) as proxy for gene expression in different tissues.

#### EST data

Information on tissue-specific gene expression was acquired by blastn search of chickpea UGT genes against the National Centre for Biotechnology Information (NCBI; http://www.ncbi.nlm.nih.gov/) EST database applying standard genetic code, E-value threshold 1 and gap existence and extension cost set to 11 and 1.

## Results

### Identification of *Ca*UGT proteins

Chickpea UGTs from its predicted proteome data were identified using a stand-alone blastp search of PSPG motif as query against 28,269 chickpea gene models. 125 UGT sequences with lengths ranging from 126 to 596 amino acids could be identified this way. However, 15 of these sequences showed comparatively higher E-value. Family 1 UGTs utilizes low molecular weight compounds as acceptors bound in the N-terminal domain and possess a highly conserved carboxy terminal signature motif (PSPG motif), involved in the binding of sugar donor (UDP sugar) in the binding site. Taking these features into account, 96 sequences possessing both the domains and length variation 410–596 amino acids were selected for further analysis, for which the following GenBank Accession numbers were assigned [GenBank ID: KC990643, KF000375–KF000405, KF006942–KF006953, KF018245–KF018279, KF039755–KF039769 and KF843731–KF843732] (Table S3, Figure S2). These 96 sequences were taken forward for the further analysis.

**Figure 1:**
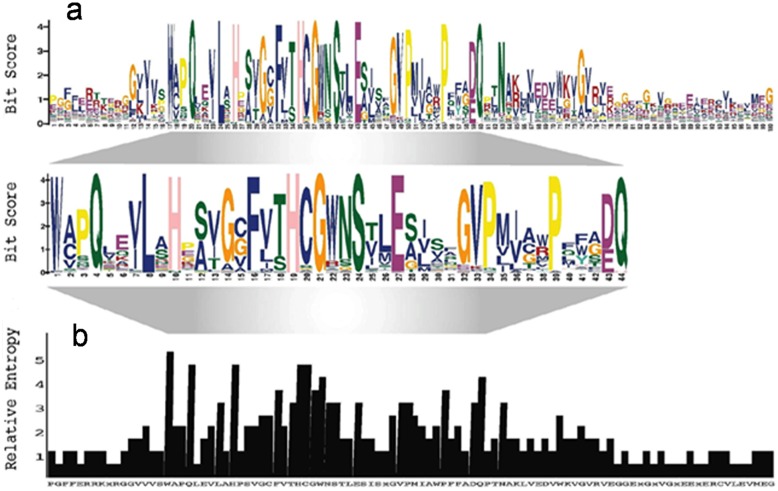
Gene identification by PSWM. Conservation [Bit score (a) and Relative entropy (b)] of the PSPG motif of 89 UGTs from various plant sources.

To further confirm the above result, PSWM was created using PSPG motif from 89 protein sequences of various plant sources with the help of MEME below E-value 2.5*10^−2741^ (Table S1). Length of PSPG motif (44 amino acids) was re-confirmed by comparing bit score and relative entropy of protein motif of long length (100 amino acids) identified by MEME (Figure 1). PSWM was then given as input to MAST of MEME suite to screen the *Ca*UGTs in the whole predicted proteome of chickpea. This method identified 124 *Ca*UGTs (with E-value below 9.9*10^−0.9^), out of which 123 sequences matched with the previously identified *Ca*UGTs using blastp. The sequence not predicted by blast (Ca_06153) was identified in the next methodology too but it was found to be a false positive hit.

Predicted proteome of chickpea was searched for the UGTs with the help of HMM-profile of Pfam Family UDPGT (PF00201). This method resulted in 129 UGT hits which matched with the hits of previous two methods. The PSPG motif of the four additional hits not predicted by BLAST was variable when compared with the remaining 125 sequences. Even these protein sequence hits (Ca_06794, Ca_06153, Ca_27131 and Ca_19130) showed slightly higher E-value as well as missing NTD or CTD and hence, were not considered in further study (Figure S3).

**Figure 2:**
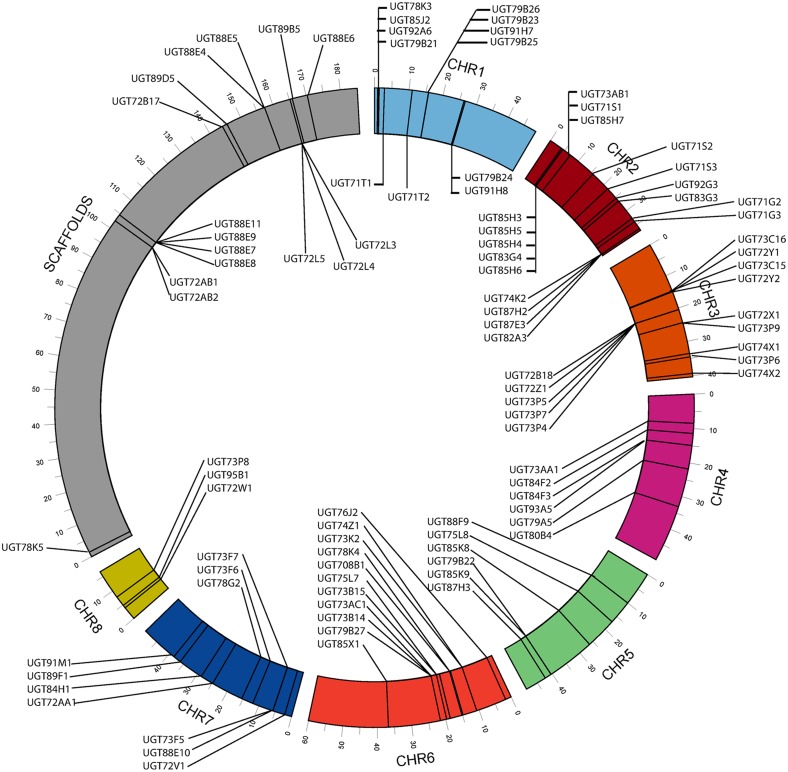
Genomic distribution of *CaUGTs*. Chromosomal distribution of *CaUGTs* in chickpea genome.

Out of total 125 predicted UGTs by BLAST, 123 sequences were identified through both PSWM and HMM profile search (Figure S4). These results confirm that most of the UGTs of chickpea were identified by following three different methodologies. Location and genomic distribution of each *UGT* on the genome is shown in Figure 2.

### Phylogenetic analysis and recent gene duplication events

**Figure 3:**
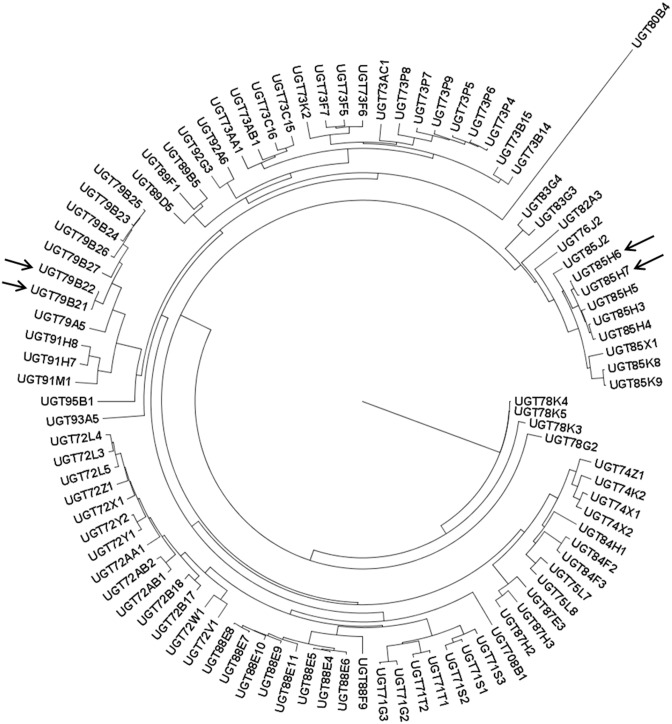
Phylogenetic analysis of *Ca*UGTs. Dendrogram showing clustering of 96 *Ca*UGTs along with two recent gene duplication events marked by arrows.

Nomenclature of UGTs used was that recommended by UGT nomenclature committee (Table S4). All 96 predicted UGTs of chickpea belong to the family UDPGT-like and UDP glycosyltransferase/glycogen phosphorylase superfamily that utilize nucleotide molecule uridine diphosphate (UDP) with attached sugar molecule to perform glycosylation reaction. A phylogenetic tree of the predicted 96 *Ca*UGTs was prepared using maximum likelihood method by employing aLRT SH-like fast likelihood-based method. The long gene of UGT80B4 bearing several introns might have diverged away from the rest. Similarly, the closely related UGT85H6 & UGT85H7 (sequence identity: 96%) and UGT79B21 & UGT79B22 (identity: 98%) might be related by recent duplication events (Figure 3).

### Functional specificity of chickpea UGTs

**Figure 4:**
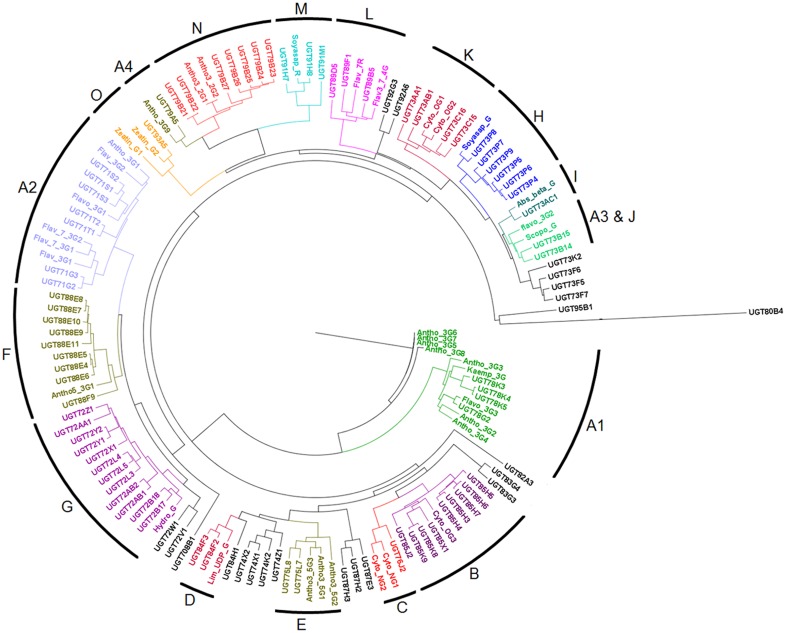
Functional annotation of *Ca*UGTs. Dendrogram showing clustering of 96 *Ca*UGTs with 38 well characterized UGT proteins from other plant species. The image shows distinct clustering of *Ca*UGTs with the functionally related UGTs.

In the combined dendrogram of 96 *Ca*UGTs and 38 selected plant UGTs, the identified UGTs clustered into 15 groups (designated A to O) (Figure 4, Table S5). Previously, we have used a strategy of comparing eight substrate binding regions of UGTs combined with clustering to identify flavonoid-3-O glycosyltransferases (F3GTs) in the database [50]. We have used a similar strategy here to identify UGT specificity. In the present analysis of *Ca*UGTs four clusters of F3GTs (A1 to A4) comprising glycosyltransferases specific to flavonol-3-O and anthocyanidin-3-O were observed. Significant conservation of the eight regions in the vicinity of acceptor binding site is present in each group of the dendrogram (Figure S5). A mixing of scopoletin glycosyltransferases and flavonoid-3-O glycosyltransferases was observed in groups A3 and J. As is known, it is possible that they indeed have mixed activity towards both the substrates [51]. Similarly, a mixed preference towards different –OH group of flavonoid in group L (flavonoid 7-O, 4′O and 3-O GT) is seen. Successful functional assignment could be achieved for 74 chickpea proteins with some reliability.

### Experimental validation of chickpea UGTs

**Figure 5:**
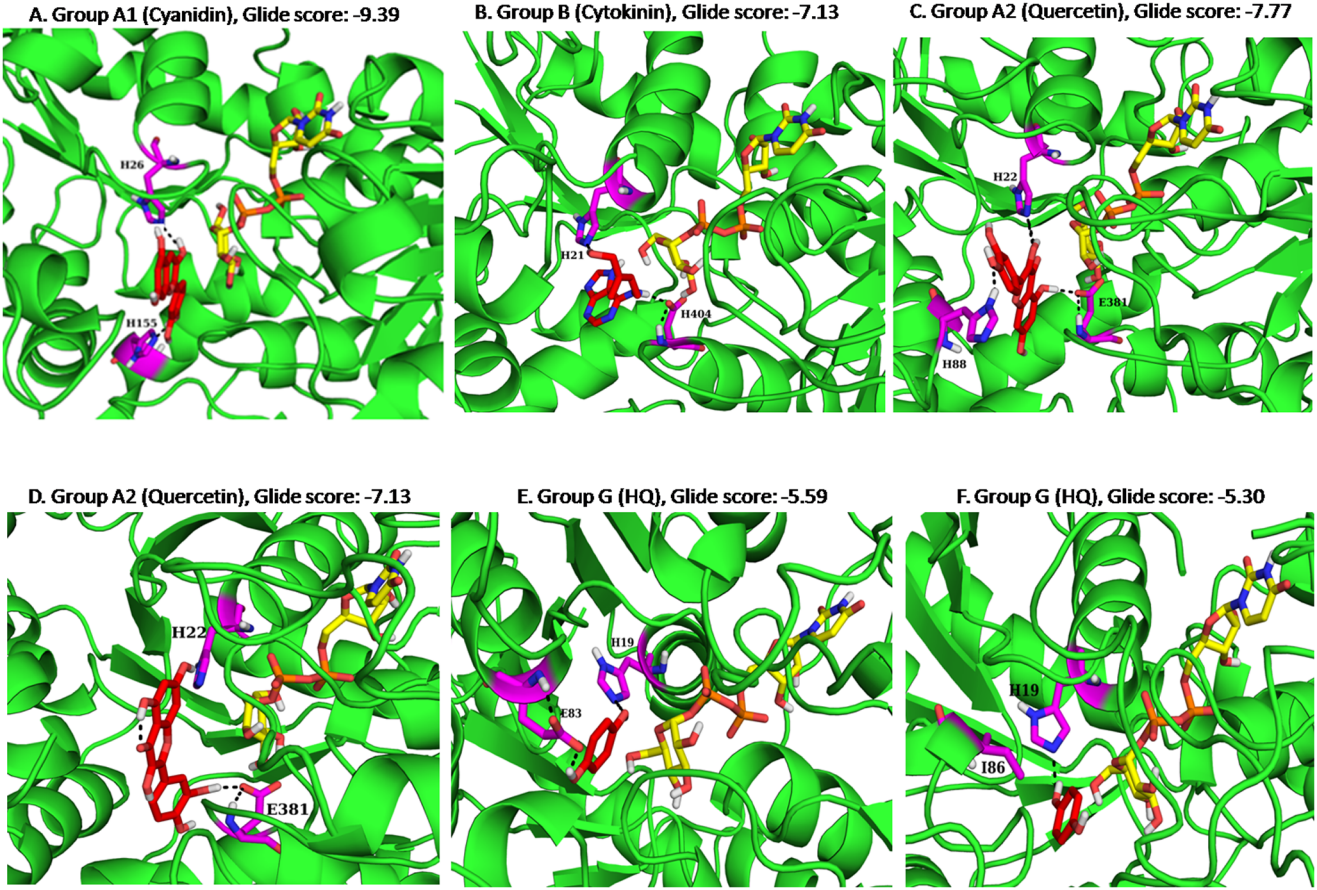
Docked complexes of *Ca*UGTs with their respective acceptor and sugar donor. **A.** The docked complex of *Ca*UGT of group A1 with cyanidin (shown in stick form) interacting with H26 and H155. **B.** The docked complex of *Ca*UGT of group B with cytokinin (shown in stick form) interacting with H21 and H404. **C.** The docked complex of *Ca*UGT of group A2 in which 3-OH group of quercetin (shown in stick form) interacting with H22. **D.** The docked complex of *Ca*UGT of group A2 in which 7-OH group of quercetin (shown in stick form) is pointing towards H22. **E.** The docked complex of *Ca*UGT of group E with hydroquinone (shown in stick form) interacting with H19 and E83 shown in stick form. **F.** The docked complex of *Ca*UGT of group G with hydroquinone (shown in stick form) interacting with H19.

Out of the total 15 groups identified, UGTs of four clusters share a significant sequence identity with the crystal structure of homologous proteins. Therefore in order to study the binding affinity and specificity for the substrates, UGT78G2 of group A1, UGT71G2 of group A2, UGT85H3 of group B, UGT72B18 and UGT72×1 of group G were modeled by taking templates of high resolution and identity with the respective targets (Table S6). The sequence alignment of the target and the templates used for model building and secondary structure prediction by Psipred showed the similarity between the templates and the target with respect to the arrangement of secondary structure elements. Very few gaps were observed in the alignment of the target and template sequences (Figure S6). The structure validation parameters revealed the high quality of the generated 3-dimensional homology models (Table S7). The docking studies with the sugar acceptors showed higher affinity towards a specific substrate as compare to others. As shown in the dendrogram, group A1 members are shown to have high similarity with the Anthocyanidin 3-O and flavonol 3-O glycosyltransferase. The best docked complex of UGT78G2 of group A1 with an anthocyanidin named cyanidin showed high binding affinity in which its 3-OH group is interacting with the catalytic histidine (Figure 5A). The group B UGTs are specific towards the glycosylation of cytokinin at the oxygen (O-glycosylation). Docking studies revealed the interaction between oxygen and NE2 atom of catalytic histidine of UGT85H3 in the best docked complex (Figure 5B). The experimentally validated proteins of group A2 showed mixed specificity towards multiple hydroxyl groups of flavonoid, the docked complex between UGT71G2 and quercetin showed the similar pattern of interactions (Figure 5C and 5D). Another group analyzed in this study was group G which is specific towards the glycosylation of hydroquinone (HQ). The conserved glutamic acid residue in region N3 and phenylalanine residue of region N4 involved in the hydrogen bond interaction and stacking interaction with the ring of hydroquinone identified by docking studies with *Solanum lycopersicum* UGT [52] are present in UGT72 family of chickpea (Group G- glutamic acid present in UGT72B17 & UGT72B18). The docking studies have shown that the UGTs with glutamate present in the N3 region interact with the –OH group of HQ (Figure 5E). Contrary to this, glutamate is substituted by other residues in some of the proteins. In UGT72x1, isoleucine replaced this glutamate and the hydrogen bond at this site was lost (Figure 5F).

### Detection of *Ca*UGT orthologs and gene divergence

Among the four papilionoideae plant genomes (*M. truncatula*, *G. max*, *V. angularis*, and *L. Japonicus*) on comparison with chickpea the maximum number of orthologs for *Ca*UGTs was detected in *M. truncatula* (143) while least number was found in *V. angularis* (only 2). Out of the 96 *Ca*UGTs, 87 had close orthologs in one of the four related dicot plants while nine UGTs seem diverged in chickpea (Table S8). The number of introns was found to be similar in their corresponding orthologs (except UGT83G4).

### Intron incursion/deletion events in *CaUGT* genes

Out of the 96 *UGT* genes of chickpea 52 have no introns whereas 26 have one intron each in them. Two *UGTs* (UGT83G4 and UGT80B4) alone showed deviations as they contain 2 and 13 introns, respectively (Table S3). On the contrary ortholog of UGT83G4 (*M. truncatula*: XM_003621376) has only one intron, thus some intron gain or loss event would have occurred during the evolution. Gene length varies due to the presence of introns while the overall protein length is similar in almost all of them with an average protein length of 472 amino acids. Standard deviation (SD) of the protein length calculated showed maximum deviation for UGT95B1, UGT72AB2, UGT74Z1, UGT80B4, and UGT83G4 from mean value (Figure S7, Table S9).

### Gene expression analysis using RNA-seq and EST data

**Figure 6:**
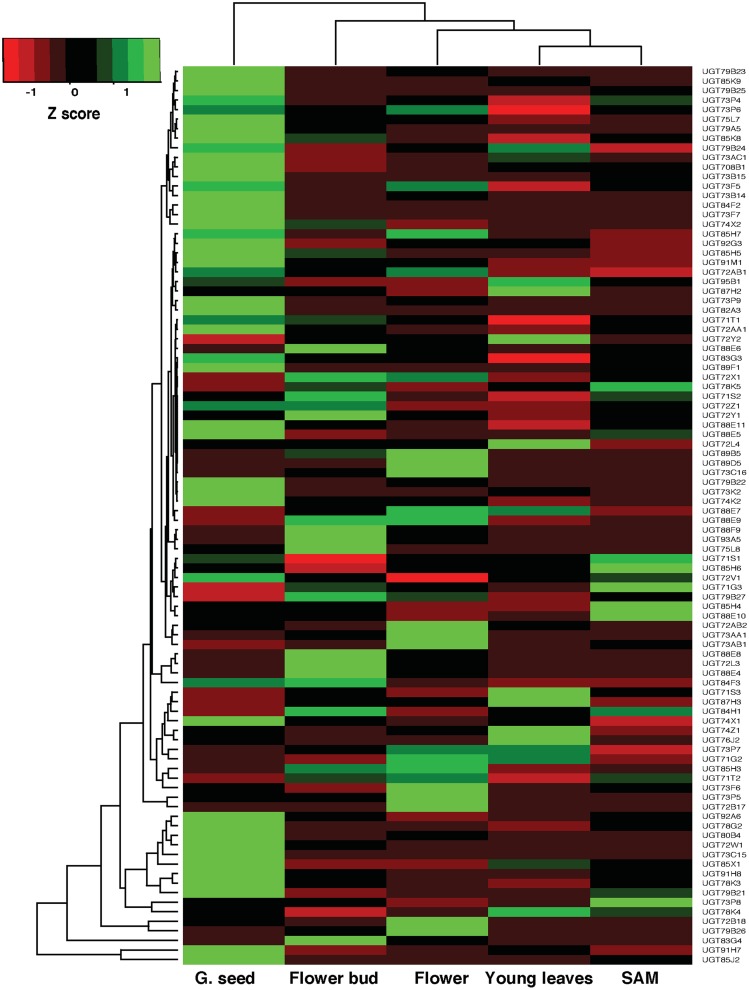
Expression level for chickpea *UGT* genes in various tissues by RNA-seq data analysis. Heatmap showing relative gene expression in various tissue samples. The color scale (−1 to 1) represents Z-score, calculated by comparing Fragments Per Kilobase of transcript per Million (FPKM) value for UGT genes in different tissues. The *UGT* genes with FPKM>0 are included in the analysis. Dendrogram on the top and side of the heatmap shows hierarchical clustering of tissues and genes using complete linkage approach.

Using RNA-seq data 84 *UGT* genes showed medium to high expression level (FPKM> = 5) in one or more tissue, whereas 10 *UGT* genes were lowly expressed (5>FPKM>0) and 2 (UGT72L5 and UGT87E3) showed no expression (FPKM = 0) in all the five tissues examined. Differential expression patterns were observed across the tissues with most of the *CaUGTs* showing highest expression in germinating seeds (Figure 6). To investigate whether it is due to sample bias, as most of the genes are expressed highly in germinating seed tissue, we compared distribution of expression values (FPKM) for all the genes in five tissues considered in the study. Expression distribution didn’t show any obvious bias (Figure S8 & Table S10).

Gene expression for *CaUGTs* was identified also by carrying out blastn search against the chickpea EST database available at NCBI. We have used ≥90% sequence identity criteria to map the ESTs over gene models. Expression has been observed for 19 *UGTs* out of 96 in various tissue types such as root, shoot, stem and leaf. Out of 19 *CaUGTs*, 13 have shown expression in the root tissue of chickpea (Table S11). However, these 19 *UGTs* are also showing expression in the RNA-seq analysis although the plant tissues tested happen to be different. The gene expression matches for specific genes when checked in the same tissues by using both the methods.

## Discussion

Glycosyltransferases are part of an essential multigene family present in all species including bacteria, fungi, animals, plants etc. In plants, they perform glycosylation of important plant products which helps in their proper functioning as well as survival in adverse situations. Genome sequencing projects help the researchers to analyze the new data and get useful information out of it. Gene identification methods based on biochemical studies and characterization are difficult as well as time consuming, therefore in the present research identification of novel *UGT* genes of chickpea was carried out by screening the signature motif of UGTs as well as by aligning HMM profile of UDPGT family with the predicted proteome. Very few sequences were identified exclusively by MEME-MAST and HMM profile search but not by blast search. None of these sequences possess the key features of UGTs therefore might be considered as false positive hits**.** Two possible recent gene duplication events and nine diverged *Ca*UGTs were found. Maximum number of *CaUGT* genes has only one intron in them while two *CaUGTs* have two introns each and one has thirteen introns. The phylogenetic tree can be useful to deduce the structure-function relationship of these predicted UGTs and further assist in their functional analysis. The phylogenetic analysis carried out combining with the UGTs of known specificity helped us to achieve functional assignment of 74 chickpea UGTs.

Our results are consistent with the previous findings that expression of *UGTs* was localized to regions of rapidly dividing cells [53]. High expression of *UGTs* coinciding with tissues involved in intense cell division (germinating seeds, flower etc.) indicates possible involvement in cell cycle regulation. Gene expression analysis not only confirmed that 84 (out of 96) *CaUGTs* significantly expressed but also revealed tissue-specific role as possible explanation for their high content.

Phylogenetic tree generated by exploiting the activity information of other experimentally validated proteins revealed distinct clustering for all the 15 identified groups. The eight regions, identified by us in our previous study, in the proximity of the sugar acceptor were found to be highly conserved, which shows their selectivity towards specific sugar acceptors. The above findings were further supported by the docking simulation studies. These findings are very useful in assigning the putative functions to the identified chickpea UGTs and can be further validated by experimental approaches.

UGT class of enzyme constitutes approximately 0.4% of the total predicted chickpea proteome, which is quite a significant number for one particular class of enzyme. If we consider other sequenced genomes like *Arabidopsis thaliana* and *Oryza sativa*, similar pattern of occurrence of UGT genes has been observed [54], [55]. Such high abundance of ubiquitous GT family in any plant genome must have indispensable role in the glycosylation of diverse array of acceptor substrates and perform distinct functions. Previous studies have shown the role of higher duplication rate behind the expansion and high content of UGT gene family in a genome [56], [57]. The phylogenetic analysis of chickpea UGTs can be beneficial for understanding the structure-function relationship and might further assist in their functional analysis. Identification of novel chickpea UGTs helps in developing genetically modified genes and their products with improved properties and thus to develop plants that react efficiently to adverse or stress conditions.

## Supporting Information

Figure S1 **Surface representation of UGT88E9 with bound quercetin (Yellow) and UPG (blue) shown in stick form.** The NTD and CTD are shown in red and green color with the interdomain linker marked by arrows (The image is drawn in PyMOL).
(TIF)

Figure S2 **Multiple sequence alignment of 96 chickpea UGTs.** The important conserved residues of PF00201 pfam family are marked with an arrow. This and the following sequence alignments are generated using ClustalX [58].
(PDF)

Figure S3 **Multiple sequence alignment of four chickpea UGTs [Ca_06794, Ca_06153 (by MEME-MAST), Ca_27131 and Ca_19130] identified by HMM search.**
(PDF)

Figure S4 **Gene identification statistics.** The number of *Ca*UGTs predicted using various methods such as PSWM search in MEME-MAST, Blastp and HMM-profiles shown with the help of a Venn diagram.
(TIF)

Figure S5 **Multiple sequence alignment of chickpea UGTs with experimentally validated UGT proteins.** Regions marked in boxes are important for acceptor specificity.
(PDF)

Figure S6 **Multiple sequence alignment of chickpea UGT protein sequences and their respective templates utilized for the homology modeling studies.**
(PDF)

Figure S7 **Standard deviation plot of chickpea UGTs.**
(TIF)

Figure S8 **Distribution of expression (FPKM) values for all the expressed genes in various tissues.** Violin plot representing distribution of FPKM values of all the expressed genes (FPKM>0) in different tissues. Natural logarithm scale of FPKM values was plotted to reduce the range of FPKM values.
(TIF)

Table S1 **Sequence information of 89 UGTs dataset.**
(XLS)

Table S2 **Sequence information of 38 UGTs dataset.**
(XLS)

Table S3 **Summary of 96 chickpea UGTs: information of genes and intron size, numbers, phase and positions.**
(XLS)

Table S4 **Gene nomenclature of chickpea UGTs.**
(XLS)

Table S5 **Functional specificity of chickpea UGTs.**
(XLS)

Table S6 **Statistics of Blast results.**
(DOC)

Table S7 **Structure evaluation statistics of generated homology models of** ***Ca*** **UGTs protein sequences.**
(DOC)

Table S8 **Orthologs of chickpea UGTs in four selected dicot plants.**
(XLS)

Table S9 **Standard deviation of protein lengths of** ***Ca*** **UGTs.**
(XLS)

Table S10 **Expression values (FPKM) of all the chickpea UGT genes in various plant tissues.**
(XLS)

Table S11 **Description of** ***Cicer arietinum*** **EST BLAST hits against the chickpea dbEST in NCBI.**
(XLS)
