# Supplementary figures and images for: Genome-Wide Identification and Tissue-Specific Expression Analysis of UDP-Glycosyltransferases Genes Confirm Their Abundance in Cicer arietinum (Chickpea) Genome
Source: PLoS One. 2014 Oct 7;9(10):e109715. doi: 10.1371/journal.pone.0109715 (PMC4188811; doi:10.1371/journal.pone.0109715)

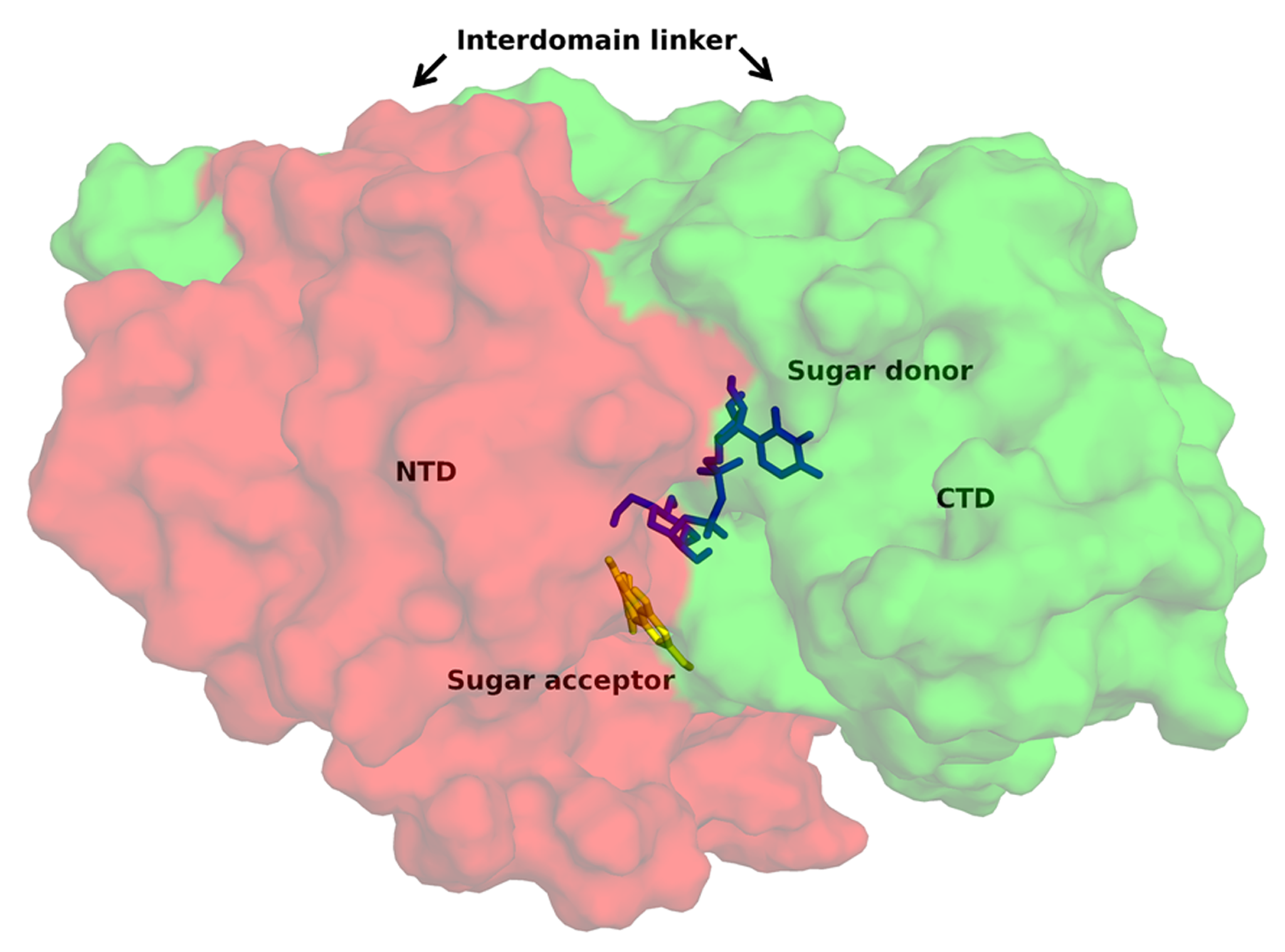

Supplement: Figure S1 — Surface representation of UGT88E9 with bound quercetin (Yellow) and UPG (blue) shown in stick form. The NTD and CTD are shown in red and green color with the interdomain linker marked by arrows (The image is drawn in PyMOL). (TIF) [file pone.0109715.s001.tif]

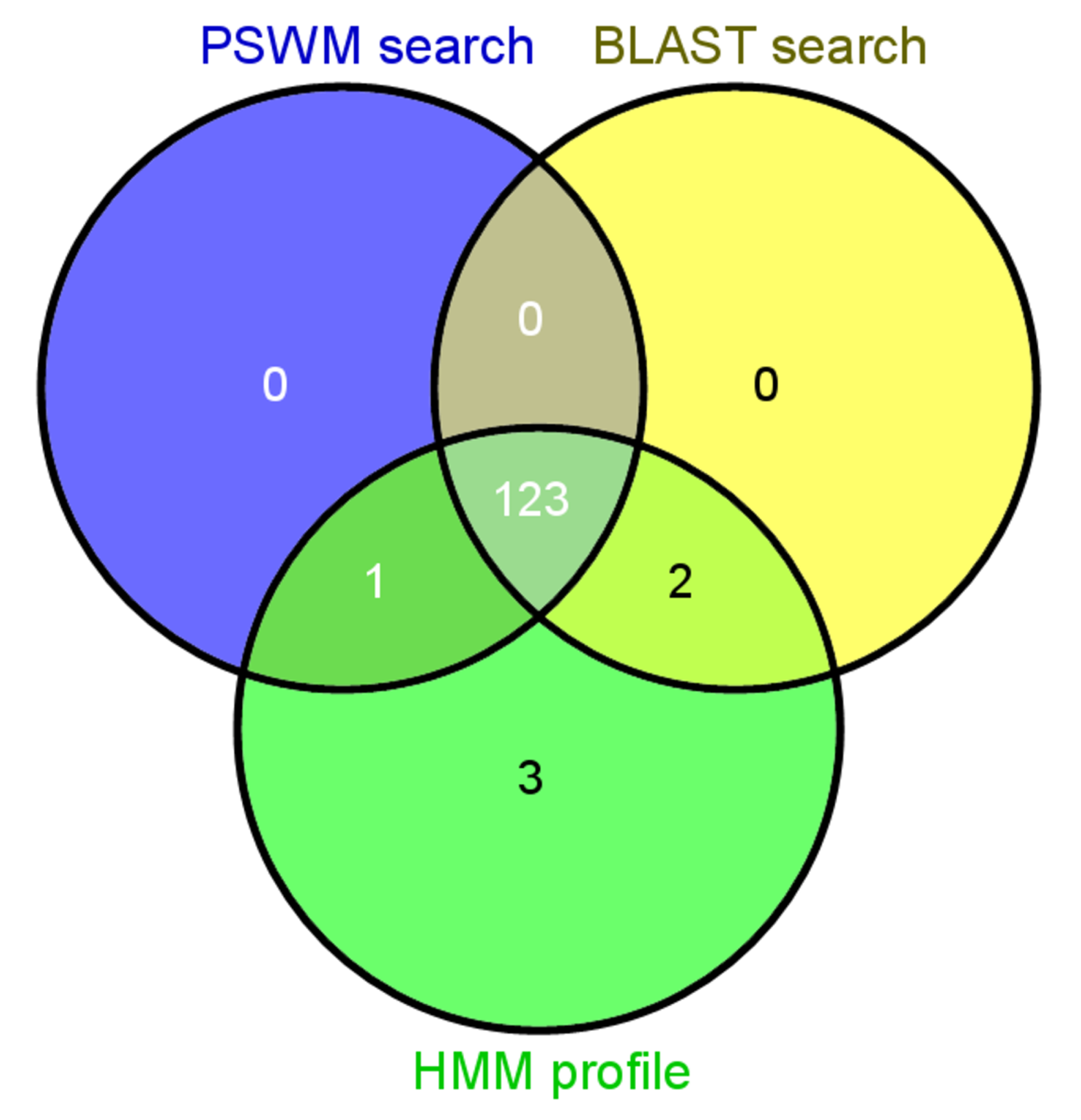

Supplement: Figure S4 — Gene identification statistics. The number of CaUGTs predicted using various methods such as PSWM search in MEME-MAST, Blastp and HMM-profiles shown with the help of a Venn diagram. (TIF) [file pone.0109715.s004.tif]

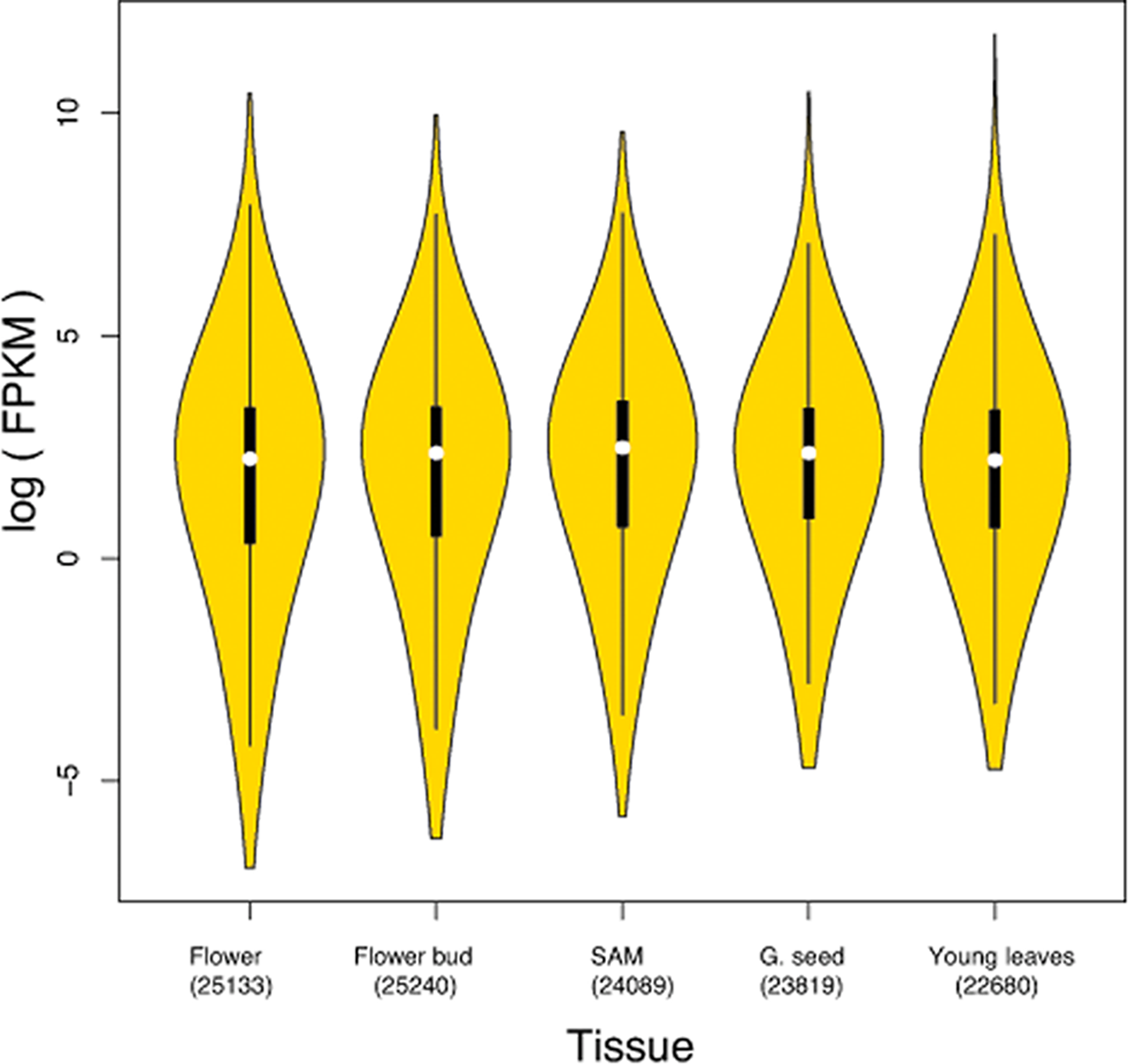

Supplement: Figure S8 — Distribution of expression (FPKM) values for all the expressed genes in various tissues. Violin plot representing distribution of FPKM values of all the expressed genes (FPKM>0) in different tissues. Natural logarithm scale of FPKM values was plotted to reduce the range of FPKM values. (TIF) [file pone.0109715.s008.tif]
