# Supplementary material for: Genome-Wide Identification and Tissue-Specific Expression Analysis of UDP-Glycosyltransferases Genes Confirm Their Abundance in Cicer arietinum (Chickpea) Genome
Source: PLoS One. 2014 Oct 7;9(10):e109715. doi: 10.1371/journal.pone.0109715 (PMC4188811; doi:10.1371/journal.pone.0109715)

Figure S2 Multiple sequence alignment of 96 chickpea UGTs. The important conserved residues of PF00201 pfam family are marked with an arrow

```
UGT708B1   -------------MSDEAVHVAMFPSAGMGHLTPFLRLAALLLNNHCKVTLINPLPTVSHAESNLLSHFHSSFPHLNILP   67
UGT71S1    --------------MMKKSEVVFIPFPGPTHLISTLEFAKLLINRDNRLRITVLVFKFPHTPDTDIHTKSLPISDS-LHL   65
UGT71S2    ---------------MKKAEVMFIPSPGLGHLVSTLEFAKLLINRDNRLRITVLVMKFPNTTETDIYTKSLPITDS-LQL   64
UGT71S3    ---------------MKKIEVVFIPSPGVGHLISTLEFANLLINRNNRLNITVLVINFPKTVEKQTN-YSLTESEN-LHV   63
UGT71G2    --------MALNEMHNKKSELIFIPSPGIGHLASSLEFAKLLINTNTNLHITVLCIKFPGTPFSDSYIKTVLNSQPQIKL   72
UGT71G3    -------------MKNKKAELIFIPSPGIGHLTSSLEFAKLLINTNTNLHITVLCINLPDTPFSDSYIKSVLNSQPQIHL   67
UGT71T1    ----------------------------MGHIVSTVEFANYITKNHPEISTTILIITMPHTSLVNTYTQSRASTDTNLRF   52
UGT72V1    ------------MDFHKPPHIVLLSSPGLGHLIPIIELAKRFIIHHKFNLTILAVTS------QTSHAESQILKSATNSS   62
UGT72Y1    ---------------MGKHHIVVVPSAGFTHLVPIIEFSKRLVHLHPQFQITCIIPS------IGSPPSSSKSYIQTLPP   59
UGT72B17   -----------MENQTLPPLVTMMPSPGMGHLIPMIEFAKRLINHH-NLHVTFIIPS------DGPPSKAQITVLTSLPR   62
UGT72W1    -------------MVSPKTHVVLLASPGMGHLIPTIELGKRLTTHHNLNATVFVVTT------DHS-SDTRTSNILQQTS   60
UGT72Y2    ---------------MEKRHIVVVPSAGFTHLVPILEFSKRLVHLHPQFEITCIIPS------IGSPPSSSKSYLQTLPP   59
UGT72B18   -------------MAPKLPMVAMLPSPGMGHLIPMIEFAKRIILHH-NIHVTFIIPT------EASPSKAQISVLQSLPK   60
UGT72AB1   --------------MEKKRCIAMIPSPGLSHLIPLVEFAKLLLQKHNEFIITFLIPT------FGPLSSSMQSILNTLPP   60
UGT72Z1    --------------MAKTIHIAVISIPAFSHQASIIQFCKNLIHLHNHFHITCIFPT------IDSPLPSTLTLLKSLPP   60
UGT72AA1   --------------MDRKTHIAVVTVPIVSHQISISEFCKKLLNLNPNIHITLIIPV------LDSLSNASKSIIASLSS   60
UGT72L3    --------------MEKPIHIAVVPGVGYSHLNPILQFSKLLVHLHPHFHVTCFIPS------LGSLPTDSKTILQTLPS   60
UGT72L5    -------MNLD---MEKTIHIAVVPGVGFGHLVPILQFTKLLVHLHPFIHVTCLIPT------LGSPPSALKTILQTLPS   64
UGT72X1    --------------MAKTTHIAVIPSPGFSHLVPILEFTKRLVTNHPNFHITCIIPS------LGSPPNSSKSYLQTIPP   60
UGT72L4    -------MNMNTNFHGEPIHIAVVSGVGYSHLVPILHFSNILVNLHPYIHVTCLIPS------LGSPPNSSKSILQTLPS   67
UGT73K2    -----------METKSKPIKIYMLPFFAQGHLIPLVNLAR--LVASKN-QKVTIITTPSNAQLFDKTIEH-DKATGSFIN   65
UGT73AA1   ----MTQEVAMDSTKLSNVHIVCIPFMAPGHILPMVDMAK--LLARHN-VKVTIITTPLNAIPFKTNINK-EIQLGSPIQ   72
UGT73AB1   -----------MAFEFKHLHFVLVPLFAQGHMIPMIDMAK--MLAEQS-VMVTLVTTPQNTSRFHNTFCR-ATKSGLSIN   65
UGT73C15   -----------MESKYPKLHFVLFPMMAQGHMIPMIDIAK--ILAQYENVIVTIVTTPHNASRFTSILARYNLESGFHIQ   67
UGT73F5    ---------MDLEIEEKPLKLYFIPYLAPGHMIPLCDIAI--QFAARR-HHVTIITTPFNAQILTKSLSF-N----QNLR   63
UGT73F7    --------------MDKPLKLYFIPYLAAGHMIPLCDIAS--LFASRG-HLVTIITTPSNAQIISKSN---------NLN   54
UGT73F6    --------MGALNLEDLPLKFHFIPYPAPGHMIPLCDIAT--LFASCG-HHVTIITTPSNAQILLKSIPS-H----HNLH   64
UGT73P5    ---------MESQ--SQQLNVIFVPYPTPGHMIPMIDTAR--LFAKHG-ANVTIITTHANASTFQKSIDS-DFNSGHPIK   65
UGT73P7    ---------MESQ----QLNIVFLPYPTPGHMIPMIDTAR--LFAKHG-ANVTIITTHANVSSFQKAIDS-DFSAGHSIK   63
UGT73P4    ---------MDSQ--SQQLNVIFLPYPTPGHMIPMIDTAR--LFAKHG-ANVTIITTHANASSFQKAIKS-DFNSGHPIK   65
UGT73B15   ---------MGNE--NNDLHIILFPFLANGHIIPCIDLAR--LFFSRG-LKVTIITTHLNVPLISRTIGK-SK-----IN   60
UGT73AC1   ---------MDHE--TDSVRIFFFPFVGGGHQIPMIDTAR--VFAKHG-AISTILTTPSNALQFKNSITR-DQKSNLPIT   65
UGT73B14   ---------MASQ--NNPLHIFFFPFMGHGHMIPSIDMAK--LFASKG-VKATIVTTPLNKPLVTKAIEQ-FKTHSNNID   65
UGT73P9    ---------MESQQLHNQLNVIFLPFPTPGHMIPMIDTAR--LFAKHG-ANVTIITTHANASTFQKSIDN-DFNSGHPIK   67
UGT74X1    ---------MEKK----TVHCLVLSFPAQGHINPMLQLSKLLQHEGVK---VTLVTTLYYRKSLQ--SIPSSIA----IE   58
UGT74K2    ---------MEKERKQHVPHCLILPYPAQGHMNPMIQFSNRLIQKQVK---VTLITVVSLWKTITNKNLSSNIQ----VE   64
UGT75L7    --------------MPHRHHILLIPYPVQGHINPAFQFAKRLISLGAH---VTLSTTLHMHNRITHKPTLPNLS----YL   59
UGT75L8    --------------MSHNHNFLIVTYPLQGHINPALQFAKRLVTMGAH---VTFTTTIYLHRRLINKPTIPNLS----FA   59
UGT76J2    -----------------------MASPFQGHITPLLQLAHILYTNAFSITILHTIFNSP---------NTSSYP-HFTFL   47
UGT78K3    ----------MTISSDHIKHVAVYVFPFGSHPVPLLNLVLKLAHASPN---TLFSFIGTQQSNKPLFSKPN-IPNNIKPF   66
UGT78K4    ----------MSNSNENKKHIAVFPFPFSSHLLPLLTLTINLAHESPN---SHFSFISTQKTNQTLFSKTN-IPKNIKHY   66
UGT78K5    ----------MSNSNENKKHIAVFPFPFSSHLLPLLTLTIKLAHESPN---SHFSFIATEKTNQTLFSKTN-IPNNIKHY   66
UGT78G2    ----MMPNLLKDETNVEKLHVAILAFPFGTHASPLLSLVRKIAGEVPK---VTFSFFTTTASNATLFSPNNDCLPNIKHY   73
UGT79B21   ------MVAN-------IFHVAMYPWFALGHLTSYLHISNKLAERGHKISFLMPKNTISKLEH----FNLHPNLISFIPI   63
UGT79B22   ------MVAN-------IFHVAMYPWFALGHLTSYLHISNKLAERGHKISFFIPKNTISKLEH----FNLHPNLISFIPI   63
UGT79B27   ------MDSSH------PLHIAMFPWFAMGHQTAFLHLSNKLAKKGHKITFFTPQKAESKLKP----FNLHPESITFITI   64
UGT79A5    -MSSEAMSNND------KLHVFMFPFLAFGHISPFVQLSNKLFSHGVQITFLSASSNIPRIKST---LNLNP-SIKIIPL   69
UGT79B26   ------MEAP-------PLHLAMFPWLAMGHFTPYLHLSNKLAKRGHKISFFIPKNTLNKLQH----LNLNPHLITFVPI   63
UGT79B23   ------MDTP-------PLHIAMFPWFAMGHLTPFLHLSNKLAQRGHKISFFIPKKTQTKLQH----LNLHPHLITFFTI   63
UGT79B25   ------MEAP-------PLHIAMFPWFAMGHFTPFLHLSNKLAQRGHKISFFIPKKTLNKLQH----LNLHPHLITFFTI   63
UGT79B24   ------MEAP-------PLHLAMFPWFAMGHFTPFLHLSNKLAQRGHKISFFIPKKTLNKLQH----LNLHPHLITFFTI   63
UGT80B4    MEDDELVSVAVEVASQQDDDLASSNLEHQSLQVTSLSHRDSVHEGLEHCITAPIGAERNPLIGEDEVMISRSMTEKRVLR   80
UGT82A3    ---------------MKKPIVILIPYPAQGHVTPMQNLASTFVSQGFEAVIVLPQYVHKKIIDSDDENIKWVG-------   58
UGT83G4    ---------------MGIPHFLVIPYPIPGHVNPLMQLSNLLAKNNCKITFLNTEFSHKRTNNISKDNLKNDQPTTINFV   65
UGT83G3    --------------MAILPHFLAIPFPVQGHVNPLMQFSQILTKYGCKVTFLHTEFSHKRSN---TDDYQNDK---IKVV   60
UGT84F2    ------------MGSEAPMKILLVSYPAQGHINPLLRLAKSLAAKGSS---VIFTTTEQAGKSMLTVKNITNKT----VT   61
UGT84F3    ------------MGSKAPIHILLVSYPAQGHINPLLRLAKCLAAKGSS---VIFTTTKKAGKDMQTGCNITDKT----VT   61
UGT84H1    ----------------------MVSAAFQGHINPMLKVANRLISNGFH---VTFAITENCRDNILKNIPNSNSS----SG   51
UGT85H3    -------MRNFTN---RKPHAVLTPYPAQGHINPLLKLAKLLHLRGFHITFVNTEYNHKRLLKSRGPNSLNGFT-DFNFE   69
UGT85H5    -------MSNYAE---TKPHAVLTPYPLQGHINPMFKLAKLLHLRGFHITFVNTEYNHKRLLKSRGPNSLDGFI-DFSFE   69
UGT85H4    -------MNNYAE---KKPHAVLIPYPLQGHINPLFKVAKLLHLKGFHITFVNTEYNHKRLLNSKGPNSLDGFT-DFNFE   69
UGT85H6    -------MSYITD---KKPHVLLIPYPAQGHINPLLKLAKILHFKGFHITFVNTEYNHKRLVESKGPNSLDGFT-DFNFE   69
UGT85H7    -------MSYITD---KKPHVLLIPYPAQGHINPLLKLPKILHFKGFHITFVNTEYNHKRLNESKGPNSVDGFT-DFNFE   69
UGT85J2    -------MDLLADGTKKKPHALLIPFPIQGHINPFLKLAKILHNKGFYITFVNTEFNHKRLFKSIGPNALNGFQ-DFQFE   72
UGT85K8    -------MDSISPHT-KKHHAVCIPFPAQGHVNPLMQLAKLLRCMGFHITFVNTEFNHKRLVNSLGQDFVKDIP-DFEFE   71
UGT85K9    -------MDTT-PHK-QKPHAVCVPFPSQGHVNPFMQLTKLLRCMGFHITFVNTEFNHKRLVKSLGQDFVKDIP-DFEFE   70
UGT85X1    -------MNSE-MDA-ERPHVICVPLPAQGHINPMLKLAKLFHHIGFFITFVHTKFNFDRLIKSNGPNSLKGLP-DFRFE   70
UGT87E3    --------MEPPLTS--LCHVVAMPYPARGHINPMMNLCKLLVSKNNN-IIVTFVVTEEWLSFISPDPKPDNIC----FR   65
UGT87H3    --------MEIPTPSMLNGHVVAIPYPTRGHINPMMNLCKHLASSSAS-LLITVVVTQEWLGFIGSDPKPNNIH----FA   67
UGT87H2    --------MNFSGEGGDICHVVAMPFPGRGHINPMLSLCKILASRKQTNILITFVVTEEWLGYIGADPKPEAIR----FA   68
UGT88E4    ----------------MKDTIVLYPALGSGHLMSMIELGKLILTHHPPFSITIFILTPPTNNNNNQHILSHTQQYIASVT   64
UGT88E5    ----------------MKDTIVLYPALGSGHLMSMIELGKLILTHHTSFSITILILTPPTNNNNNQHLLSHTKQYIASVT   64
UGT88E6    ----------------MKDTIVLYPALGNGHLMSMIELGKLISTHHPSFSITILILTPPTNNNNNQHTLSHTQQYIASVT   64
UGT88E11   ----------------MKDTLVLYPALGKGHLNSMVELGKLILTHNPSLSITILILSPPNTK--PQSLQDIQTLVKAFAC   62
UGT88E9    ----------------MKDTIVLYPAYGSGHLMSMVELGKLILTHHPSFSIKILILTPPNN----KNNNDSTTQYIASTS   60
UGT88E10   ----------------MKDTIVLYPAVGSGHLMSMVELGKLILTHHPSFSIQILILNPPNQNITTNNNNDSTTQYTTSIS   64
UGT88E7    ----------------MKETIVLYPAFGSGHLMSMVELGKLILTHHPSFSITILILTPPNN----KNN-DSTTQYIASIS   59
UGT88E8    ----------------MKDTIVLYPAYGSGHLMSMVELGKLILTHHPSFSITILILTPPNN----KNN-DSTTQYIASIS   59
UGT88F9    --------------MVETTTIVMYPAPSIGHIISMVELAKLVIQHK--ISITILLTTGFLDH-------PSIDSYINRIS   57
UGT89F1    -------------MSTLQSHLLAYPFPSSGHVIPLIDFTKNLVTRG---VHVTILVTPSTQNLVP---TNFSP----LLQ   57
UGT89D5    -------------MSNSKTHILVFPYPAQGHMLPLLDLTHHLALQT--TLTITIIITPKNLPTLTPLLSTHPN----TIQ   61
UGT89B5    -------MTIQSGSTNPPPHILVIPFPAQGHMIPLLDLTHKLASTNKNNLTITILTTPKNQTFLTPLLNSHPS----IIH   69
UGT91H7    ------MDNKNN----KPLHVVMFPWLAMGHMYPCFEVSKILAQNGFFVTILSTPSIINRLPKLPQTLSQFIHLIKLPLS   70
UGT91H8    -MGSIVNDNNNNNIEHKPLHVVMLPWLAMGHIYPYFEVAKILAQKGHTVTLINSPKNIDHIPKTPKTLQPFIKLVKLPL-   78
UGT91M1    ------MAED-------IIHVVMLPWLAFGHLIPFFKLSIALAKSGVHVSYISTPKNIQRLPKLPSTLSHLINLVEISL-   66
UGT92A6    --------------MEEKQSIVLFPFMAQGHIIPFLALALHLEQK-NKNHNITIINTPLNIQKLKSSLP-----QNSKIK   60
UGT92G3    ----------MAETTKKKGHIVMIPFMAHGHLIPFLALARKIQQTTNTIYTITIASTPLNIQHLKSAISTTFSNNDISIH   70
UGT93A5    ----MGSKDESNSKSHNQVVVIMVPFPAQGHLNQLLHMSCLILSHNIPVHYVGTSTHNRQATIRVQGWDQNFISKIHFHH   76
UGT95B1    --------------------------------------------------------------------------------
UGT71T2    -------------------------------MVPTVEFANHLTKHNPQFSATILFITMPHRPLVNTYAQSRASTATNLRF   49
UGT72AB2   ----------------------------------------------------------------------MQDILYSLPP   10
UGT73C16   -----------------------------------MDIAK--LLAQRG-VIVTIFTTPKNASRFTSVISR-AVSSGLQIK   41
UGT73P6    -------------------------------MNPMIDTAR--LFAKHG-VSVTIITTQANALIFQKAIDN-DLHSGYFIK   45
UGT73P8    -------------------------------------MAR--LFALHD-VEVTIITTKYNSTIFQNSIDL-DSSRGRSIT   39
UGT74X2    ----------------------------------MLQFSKLLKYQGVK---VTLVTTFFFAKNLQ--KLPPSIT----LE   37
UGT74Z1    ----------------------------------------------------------MHLKSSS---LPSTLQ----FD   15
           1.......10........20........30........40........50........60........70........80
```

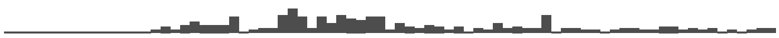

```
UGT708B1   FHLPLPS-----------SSPPSNSIDPFFFRVQTLRDSIH----LLPPLLSSLS----------PPLSAFISDIMLISP   122
UGT71S1    INLPECS-------IPPTTDPIST----MTALLESQKLNVK---QAVTNIITRE-----------GNLTAFVVDMFCTTM   120
UGT71S2    INLPECS-------IPPSSDLPSS----ITALLESQKSNVR---QVVTNLTTGEQ---------NGNLAAFVVDMFCTTM   121
UGT71S3    INLPQTTTH-----VPSTSDVGNS----ISALVETQKSNVK---QAVSNLT--------------GTLAAFVVDMFCTTM   117
UGT71G2    IDLPEVE--------QPPHELLKSHEFYILTFMESLIPHVR---STLQTITS-------------NQVVGLVLDFFCISM   128
UGT71G3    IDLPKVE--------PPSKELFITPPSYFKTLIETLIPHVK---ATIQNILSSHS----------NQVVGLVLDFFCISI   126
UGT71T1    IYLPTVD--------PPTPDQYQSLNGFLSLLIQNHKNNIK---NALLDIMITESTSES------VKLAALFIDLFSTTI   115
UGT72V1    -LYTIIQIP-----SPNISSLLPTSATVSTRLSVTMRQAIP---SIKSALTNTPL-----------PPSALIVDIFGTES   122
UGT72Y1    -SISSIFLP-----PIILDQVPDD-EILAFQIELSVKYSLP---HIKQELKSLCS---------RSKVVALVVDVFAHDA   120
UGT72B17   -GISHIFLP-----PITLSDLPPN-TKIEPLISLTVLRSLP---SLRQTILSLTS---------SHRIAALTVDLFGTDA   123
UGT72W1    -NLNIVLVP-----SIDVSDKLPPKPSLVARIILTLIESLP---FIRSQIQSMEL-----------PPSSLIVDIFGTVA   120
UGT72Y2    -SISSIFLP-----PIILDQVLDP-KVLAVQIELSVTQSLP---FIKQELNSLCS---------RSKVVALVVDVFAHDV   120
UGT72B18   -SISHIFLP-----PVSFADLPSN-TKIETLISLTVLRSLP---SLRQTFRSLSD---------SYTLTAVIVDLFGTEA   121
UGT72AB1   -NMNFTVLP-----QLNNIQQDLP-IEPATQMKLIVKNSVP---FLIEEVKSLIS---------KTHLIALVFSMFSTDA   121
UGT72Z1    -NINYTFLP-----PINKQNLPQH-LSPAVQIQLAVSQSMP---SFHNTILSLSSSS-------TTPILALISDPFANES   123
UGT72AA1   LKINTIVLP-----SIN---LPPQ-TAPTLKLPLAMSLTMP---SIIETLNSITS-----------KVVAIVADYFAYEV   117
UGT72L3    -NIHCYFLP-----PLDPKNLPLQ-LPLELQLQFTVNHSLP---SLHQVLKTLTL---------KTPFVAMIVDSFAVEA   121
UGT72L5    -NINYTFLL-----PVDPNDLPQETLTLEMKSQLIVTLSLP---YLHQALKSLAL---------RTPLVALVADSFAVEA   126
UGT72X1    -NINSIFLP-----PINKHDLPQG-AYPGVIIQLTVTLSLP---SIHQALKSLNS---------KAPLVALIADSFAFEA   121
UGT72L4    -NINYTFLP-----PVHPNDLPQG-LPLEMQIEHTVTHSLP---SIHKALKSLTL---------RTPFVAMIADSFVVEA   128
UGT73K2    VHIVKFP-ATQLGLPIGVENLFAASDNQTASKIVMAAHILK---PEIEAFMKQNP------------PDVFIPDIMFTWS   129
UGT73AA1   LLEVKFP-NVEAGIPEGCESLETLPSMDLKENFMIGINLLQ---KPIEDLFEKLDP----------FPTCIICDKNIPCL   138
UGT73AB1   LLQIPFP-SQQFHLPLGCENLDTLPSRNHLRNFYNALDMLQ---DPLENYIKNHPF----------PPSCIISDKCISWT   131
UGT73C15   LIQLEFP-SKDFGLPEGCENLDMLPSLGTASNFFNASKFLE---QEVEKLIEELKS----------PPSCIISDMCLPYT   133
UGT73F5    LHTVSFP-SQEVGLPDGVENLSAATNLDNLTKIYHATTLLR---PPIQHFVEQHP------------PDCIVADFLFPWV   127
UGT73F7    VHTFQFP-SQQVGLPDGVENLTSVTDIDNSYKIFFATTLLR---EHIENFVEQYP------------PDCVIADFLFPWV   118
UGT73F6    LHTIPFP-SHQVGLPPGVENIGCANNLENSYKVHQATKLLQ---SPIHQFVEQNS------------PDCIVADSLFLWM   128
UGT73P5    TQLIQFP-SAQLGLPEGVENIKSGTSQEMLGKISQGISMLQ---DQIEIVLQDQQ------------PDCIVSDMFYPWT   129
UGT73P7    TQLIQFP-SAQVGLPDGVENIKDGTSLEMLGKITRGIWMLQ---DQIEILFQDLE------------PDCIVTDMMYPWT   127
UGT73P4    TQLIHFP-SAQAGLPDGVENIKDATSSEILGKIIHGIEMLK---DEIELLFQKLQ------------PDCIVSDMFYPWT   129
UGT73B15   IKTIQFPSPQHTGLPEGCENSESALAPDKFIKFMKATILLR---EPLEHVLQQEQ------------PNCLVSDMFFPWS   125
UGT73AC1   IHTLTEITDTDTDMSAGP---------------MIDTSVLL---EPLKQFLLQHK------------PDCIVVDMFHRWA   115
UGT73B14   IQIIKFPCVEG-GLPEGCENVDSIPSPHLIPAFFKATWLLQ---EPLEQLLQQQK------------PNCIVADMFFPWS   129
UGT73P9    TQLIQFP-SAQVGLPDGVENLKDATSPEIISKISRGISMLQ---DHIENLFQDLE------------PDCIVTDMCYPWT   131
UGT74X1    TISDG------FDNGG--LEEAGGSYITYLDQFWKIGPKTL---AELIEKLNKLG----------DTVDCVIYNSFFPWA   117
UGT74K2    TISDG------YDDGG--FESAKT-VEDYKQTFWSVGSKTL---SQLLHKLASSN----------NPPNCVIFDAFLPWV   122
UGT75L7    PFSDG------YDDGF--QSTGTDAYLLYTSEFKRRGSEFI---ANLILSNSQKG----------TPFTCLVYSLLLPWA   118
UGT75L8    AFSDG------YDDGY--NSNAIVDLSTYMLELSSRGSEFL---RNIILSAKHGN----------HPFTCLAYTLLLPWA   118
UGT76J2    PLIASLSDY--EAS---NLDAVRLTELINIRCVNPLKECFH---LLLSQ--DKED----------DRVACFISDAALYFT   107
UGT78K3    SIDDGVPKG---------HVLGSNPTEKLNLFLQAGHQNLQ---KGIDLAVAYTK----------QRVTCIISDAFVVPS   124
UGT78K4    NVSDGIPKG---------DEL--LPGSEVTFYLQTGPENFQ---NGIDLAVAESK----------KPITCIIADAFVTPT   122
UGT78K5    NVSDGIPKG---------HEL--HPGSEVNFYLQTGPENFQ---NGIDLAVAESK----------KPITCIIADAFVTPT   122
UGT78G2    NVDDGLPEG---------YVPSGHPLEPIFLFIKAMPENFK---SVMDEAVAETG----------KDITCLVTDAFYWFA   131
UGT79B21   TIPHVHGLP------LGSETTADLPFSLHS-LLMTAMDLTE---PIIEDSLRELR------------PHMVFFDFTY-WL   120
UGT79B22   TIPHVDGLP------LGSETTADLPFSLHS-LLMTAMDLTE---PIIEDSLRELR------------PHMVFFDFTY-WL   120
UGT79B27   TVPHVDPLP------PNSQTTADVPYPLQP-HIMTAMDLTR---PDIESHLLNLK------------PHIVFYDFTH-WI   121
UGT79A5    QFPN------------GISSTADLPPHLAG-NLIHAIDSTQ---PQLKSLFLELK------------PNFVFFDFAQNWL   121
UGT79B26   TVPHVDGLP------HDAETASDVPFSLLP-LIATAMDETK---KEIELLLKELK------------PQIVFFDFQY-WV   120
UGT79B23   NVPHVDGLP------YGAETTSDVPFSLFP-LIATAMDQTD---KDIELLLKELK------------PQIVFFDFQH-WL   120
UGT79B25   NVPHVDGLP------HGAETTSDVPFPLFP-LIATAMDQTE---KDIELLLKELK------------PQIVFFDFQH-WL   120
UGT79B24   NVPHVDGLP------YGAETTSDVPFSLFP-LIATAMDQTD---KEIELLLKELK------------PQIVFFDFQY-WL   120
UGT80B4    RHDLILDRLSEHEKQKLIANLVKIQNDGTVEVDVERSATVASELLELQSFEESTVSGSFISDSKKLVPRLQIVILVVGTR   160
UGT82A3    ----------------LVDGMEEDTTPDFFAIESAMENIMP---KHLEEFLQKYER---------IEVCLVVVDLLASWA   110
UGT83G4    TLPDGLEPE--DN----RSDHKKVIFSIKRNMPNLLPKLIE---DVNDLDVEN-------------KISCIVVTFNMGWA   123
UGT83G3    TLPDGLESK--DD----RSDIKKVMLSIKSTMPNKLSKLIE---DINALEVDNN-----------NKINCIVVTFNMGWA   120
UGT84F2    PIGDGSLTFELFHDGLSDDDPIKINLGAYSDQLELVGKQFV---SQIIKNHAESN----------KPISCLINNPFLPWV   128
UGT84F3    PIGDGSLTFNFFDDGFKEDDPIRTNLSDYTPQLEHVGKKHV---SKIIKNYADLK----------TPISCIINNIFIPWV   128
UGT84H1    LVLDF------FSDGLS-LDFDRSDVKTLIETLQEKGPINL---SNLIKNHTK-N----------QNFSCAIVTPFVPWA   110
UGT85H3    TIPDGLTPI--EGDGDVSQDVPSLSQSIRKNFLKPFCELLA---RLNDSANDALI----------PPVTCLVSDSCMSFT   134
UGT85H5    TIPDGLTPM--DG--DVSQDLDSLRESIRKNCYQPFCEVLA---RLKDSANDGLI----------PPVSCFVSDSFMPFT   132
UGT85H4    TIPDGLTPM--EGNGDVSQDTNSLCQSVMKNFYKPFCEVLA---RLSESANIGLI----------PPVTCLVSDCLMTFT   134
UGT85H6    TIPDGFTPM--EGDDDVSQKILSLCESIRKNFLEPFHKLLA---KLHDSAIAGFI----------PPLTCIVSDYSMSFT   134
UGT85H7    TIPDGLTPM--EGDGDVSQDMMSLCESIRKNVLEPFRKLLA---KLHDSAIAGLI----------PPLTCIVSDYSMSFT   134
UGT85J2    TIPDGLP----HTNMDATQSIPSLCDSTRKYSLAPFCNLIS---KLNDSNCA-------------PPVSCIISDGIMSFT   132
UGT85K8    TIPDGLPPF--DK--DATQDIPPLCEATRNNFYAPFKELVS---KLNS----SSP----------YPVSCIVADGSLGFA   130
UGT85K9    TIPDGLPPF--DK--DATQDIPTLCEATRNNFYAPFKELVN---KLNS----SSP----------YPVTCIIADGNFDFA   129
UGT85X1    TISDGLPPE--NE--RGIMDLPDLCKAIPIDGLISFRDIIT---KIVSL-TDSDV----------PPVTCIISDGVMSFT   132
UGT87E3    SISN-------------VVPSELTRGRDHPAFVDDVMTKME---EHFEKLLDLIE----------QPPSIIVYDTMLYWV   119
UGT87H3    TISND------------VVPLQHQIIADIPAFFLATITNIE---APFEEMLNRLH----------PPVTAIIGDLELKFP   122
UGT87H2    TIPN-------------VIPSDREKAADFLGFYEAVMTKLE---EPFERLLDRLE----------PPVNVIIGDVELRWP   122
UGT88E4    TTFPSINFY-----YIPPISFPK-THLPPHLLTLELSHQSN---HHVQNILQSIS--------KTTNLKAVILDFLTYSA   127
UGT88E5    TTFPSINFH-----YIPTISFP--TTLPPQSLTLELSHQSN---HHVQNILQSIS--------KTTNIKAVILDFLTYSA   126
UGT88E6    TTFPSINFH-----YIPPISLPS-THFPPHFLTLELSHQSN---HHVQNILQSIS--------KTTNIKAVILDFLTFSA   127
UGT88E11   ESFPSITFH-----HIPSISFS--FTLPPHILPVELSRRSN---HHVAHILQSIS--------KCSNLKGVILDFMNYST   124
UGT88E9    NKFPSINFH-----FIPSISIT--LTLPPHLYTLELSPRSN---HYVHHILQSIS--------KTTNLKAVILDFLNFSA   122
UGT88E10   SKFPSINFQ-----FIPSISLT--LTLPLHLHTLELSPHCN---HHVHHILQSIS--------KTTNLKGVILDFFNYSA   126
UGT88E7    NKFPSINFH-----FIPSISLT--STLPPHLQTLELSPHCN---HHVHNILQSFS--------KTTIIKGVILDFLNYSA   121
UGT88E8    NKFPSINFH-----FIPSISLT--LTLPPHLQTLELSPLCN---HHVHNIFQSIS--------KTTNIKGVILDFLNYSA   121
UGT88F9    TSHPSISFL-----RFPSVTVTHSNTHSLAATAFQFIKTNA---VNVQSLLRQIT--------AKTVINAFIIDLFCTSA   121
UGT89F1    TLLLPAP--QFPNP-----NQNWLVN-----VVSFMRQYHYP---ILIEWAQAQPL----------PPSAIISDFFLGWT   112
UGT89D5    TLILPFP--SHPKIPSGAENIREVGNSGNYPFINALSNLQN----LIIQWFNTHHN----------PPVALISDFFLGWT   125
UGT89B5    PLILPFP--SHPLIPPGVENAQDMPNSIRN-IMLAFSDLHH----PLLNWFNSHPS----------PPHYIISDMFCGWA   132
UGT91H7    PHIDTNHLP------QNADSTSDIPSNKLY-YLKQAYDSLQ---QSVSDVLKNSN------------PDWVFFDFAACWL   128
UGT91H8    PQIEN--LP------QGVESTMDITSHNMNRYLKLAYEGLQ---HDVTEILKTSK------------PDWVFYDFASGWL   135
UGT91M1    PSLNTNLLP------EGAEATMDIPIDKIQ-YLEEAYDQLQ---SPVKQLISNWL------------PDWIICDYSPHWI   124
UGT92A6    LLQIPFI-SSNHNLPPNTENTDTVPYNLVIKLIQASLSLQSPFKQLIQNLITQQPH----------HKFSIVSDIFFGWT   129
UGT92G3    LAELPFN-SSQYGLPPNIENTEKLPLTDIIKIFHASTSLQAPFSSLISNITEQEGH----------PPLCIISDVFLGWV   139
UGT93A5    FNVPPFDSP-------PPNPNSESKFPSHLIPSFEASSHLR---EHVKELMQSLSP--------VFKRVVVIHDSLMASV   138
UGT95B1    --------------------MSLMSPPSPEHHHDDLAKGLQN---ILSNYHRPSR------------PVCAIVDVMMNWS    45
UGT71T2    IYLPTVD--------PPTPDQYQSSIGYLCLLIQNQKNNIK---NALQNLTTTESTSESPS--DSVELAGLFIDMFSTTI   116
UGT72AB2   -NIDFILLP-----QVKNLPPNL---EPASQMKLIVKNSVP---FLIEEVESLIS---------KTHLVSLVFGVFSTDV    69
UGT73C16   IVTLNFP-SKQAGLPEGCENFDMVNSRDMICNLFLAITLLQ---KQAEELFDKLTP----------KPNCIISDFCIPWT   107
UGT73P6    TCVIQYP-TAQVGLPDGVENIKDATSHEMLGKIILGISMLQ---DQIENLLQDLQ------------PDCLVSDMLYPWT   109
UGT73P8    THIIDFP-SSKLGLPIGIESFNVNTPREMIPKIYTAIFILQ---PEIEHLFNVLK------------PDFIVTDMFYPWT   103
UGT74X2    TISDG------FDNGG--IGEAKN-FKFYLDHFWQVGPQNL---EKLIEKLGTTN----------CPIDCVIYDSFYPWA    95
UGT74Z1    FISDG------YDQGG--FAQANN-ISTYLSHMETIGSNNL---KELIKKYNFSN----------DPIDCVIYDPLIYWV    73
           ........90.......100.......110.......120.......130.......140.......150.......160
```

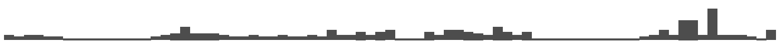

```
UGT708B1   LLSITLKLSIPNYTLFTSSASMFSFFSHFPTLSQSLSSQPISDSD-----------------------------------   167
UGT71S1    IDIAK-ELSIPSFVFFTSGIAFLGLTLHLHTLRERDNIDSTQLQ------------------------------------   163
UGT71S2    IDIAR-EFSVPAFVFFTSGVAFLGLTLHRHTLRERDNINSTQLQ------------------------------------   164
UGT71S3    IDVAN-ELGVPSLVFFTSGVAFLGLMLHLHTIWEQQ--DTELLL------------------------------------   158
UGT71G2    IDIGN-ELGIPSYMFLTSNVGFLSLMLSIKNRKIED--VFDYAD------------------------------------   169
UGT71G3    VDVGT-ELGIPSYLFLTSNVGFLGFMLSLQTRQIND--VFHDSD------------------------------------   167
UGT71T1    IDVAE-ELSVPCYLLFASPASFLGFMLHLP--------QVESIE------------------------------------   150
UGT72V1    LTLAQ-ELNIPKFIYVASHAWFLSLIVYSPILDKQIEGQYVDQK------------------------------------   165
UGT72Y1    LDLAK-ELNLLSYVYLPQAAMVLCTYFYSSQVDEILSDESRDPN------------------------------------   163
UGT72B17   FDVAA-EFNIASYVFFPSPTMVLSFAFYLPQLDQEVQCEFRDLH------------------------------------   166
UGT72W1    LPMAR-DLHMSTYVFFATSAWFSAVTIYLPFIDEEALSRHANDH------------------------------------   163
UGT72Y2    LDLAK-EFNLLSYIYLPQAAMLLSTYFYSSKLDEIISKESRDPN------------------------------------   163
UGT72B18   FDVAV-EFNLPRYVFYPSTAMALSFFLYLSRLDQEVHCEYNELT------------------------------------   164
UGT72AB1   HDVAK-QFNLLSYLFFSSGAVLFSFFLTLPNLDQSAN-TSKFLES-----------------------------------   164
UGT72Z1    LEIAK-RFNLLSYIYFPPSAMTLSLFIHLPKLHEEVSCEYRDHK------------------------------------   166
UGT72AA1   FPLAK-KHNILSYTFFPSSATVLSLCFNSTFLNETITCEFRDLK------------------------------------   160
UGT72L3    LDLAK-EFNMLSYVYFPSAVTTLSSYFHLIKLDKVTSCEYRDLP------------------------------------   164
UGT72L5    LNFAK-DFNMLSYIYFTSAATTLSFSFYFPKLDEETSCEYRDLP------------------------------------   169
UGT72X1    LDFAK-EFNSLSYLYFPSSAMNLSLSLHLVKLDEEVSCEYKDLQ------------------------------------   164
UGT72L4    LEFAK-EFNMLSYIYYPASATTLSLNLYLPKLDAETSCEYKDIQ------------------------------------   171
UGT73K2    ESTAK-ILQIPRLVFNPISIFDVCMIEAIKSHP---EAFVSDSGPYHIPE------------------------------   175
UGT73AA1   ADTSI-KLKIPRIIFDGTSCFNMLCNHNIFASKELGKFSD--LDEFIVPG------------------------------   185
UGT73AB1   TLIAQ-KFNIPRLVFHGMSCFSLLSSYNIKLHGAHRAVNS-DTDPFVIPGV-----------------------------   180
UGT73C15   IHIAR-KFNIPRISFVGVSCFCLLNLHNIHVYNMIEKMANKESEYFDLPG------------------------------   182
UGT73F5    DELAN-KLRITRLAFNGFSLFAICAIESVKAN----SHYDSAS--FLIHD------------------------------   170
UGT73F7    DELAN-KLHIPRLVFNGFSLFTICAMESLKLH----PLPEDASGSFVIPH------------------------------   163
UGT73F6    DELAN-KLHIPRLAFNGFSLFAICAMKSLKA-------HDSMS--CIIQG------------------------------   168
UGT73P5    VESAA-KLGIPRLYYYSSSYFSSCSAHFIRKYKPHENLVS-DTQFFSIPS------------------------------   177
UGT73P7    VQCAA-KLGIPRLHFYSSSYFSNCACHFIRKCKPHEGLVS-DTNKFTIPG------------------------------   175
UGT73P4    VESAA-KLSIPRLYYYSTSYFSNCSAHFIRKYKPHEDLVS-DTQIFSIPG------------------------------   177
UGT73B15   TDSAA-KFNIPRIVFHGLGFFPLCVLACIRQYKPQEKVSS-YTEPFLVPN------------------------------   173
UGT73AC1   GDIID-DLKIPRIVFTGNGCFPRCVIENTRKHVVFDNLSS-DYEPFLVPG------------------------------   163
UGT73B14   TASAA-KFGIPRIVFHGTSFFSLCASQCLNLYEPFKNVSS-DTELFEITN------------------------------   177
UGT73P9    VESAA-KLGIPRLYFYSSSYFSNCASYFVRNYRPHDSLVS-DTQKFTIPC------------------------------   179
UGT74X1    LDVAK-RFGIIGVCFLTQNMSVNSIFYNVHIG----KLKVPIIE------------------------------------   156
UGT74K2    LDVSK-TFGLLGVAFFTQSCSVNSIYYHTHQK----LIELPLS-------------------------------------   160
UGT75L7    AETAR-GFHLPTALLWVQPATMFDILYHYFHGYSESIKNPS---------------------------------------   158
UGT75L8    ANVAR-ELQLPYALLWIQAATVFDIYYYYLHEHGDYITNKSKDA------------------------------------   161
UGT76J2    QDVCD-HFGIRRFVLRTGGPSSFLVFASFPLLKQKGYFPLQE--------------------------------------   148
UGT78K3    FFLAQ-KLNVPWIPIWPPLSCSLSAHFYTDLIRQNIAENNEKDRV-----------------------------------   168
UGT78K4    FDVAK-TLNVPWIPVWIPMSCSLSVHFHGDIIRAHCSVN-DVNRT-----------------------------------   165
UGT78K5    FDVAK-TLNVPWIPVWIPMSCSLSVHFHGDIIRTHCSVN-DGNRT-----------------------------------   165
UGT78G2    ADLAQ-QINAKWVPLWTAGPHALLTHIYTDLLRHACKQVHDVQN------------------------------------   174
UGT79B21   PALAC-QLGIKALHYCTISPATVGYLISPERKL--HEKSLTEADLINPPP------------------------------   167
UGT79B22   PALAC-QLGIKALHYCTISPATVGYLISPERKL--HEKSLTEADLINPPP------------------------------   167
UGT79B27   PSVTK-ALGIKCLHYCTASSVMVGYTLSPARYS--KGKDLTEFDLMEPPP------------------------------   168
UGT79A5    PKLAS-EVGVKSIHFSVFSAISDAYIIVPSRLDDIEGRSITFEDLKKPPQ------------------------------   170
UGT79B26   PNLTK-KLGIKSVQYWIASPYSISYFFHGPRQS--LGKELTVDDLKKPPS------------------------------   167
UGT79B23   PNLTK-KHGIKSLQYLIWNPISTAYLGTGPRQS--QGRELNEVDLMKPPS------------------------------   167
UGT79B25   PNLTK-KLGIKSVQNWIMNPISTAYLGN--RPS--QGRELTEVDLLKPPS------------------------------   165
UGT79B24   PNLTK-KLGIKSVQYMIINPMTTAYLGTGPRQS--QGRELTEADLMKPPS------------------------------   167
UGT80B4    GDEYGHRVRLATHANFKTFVRSAGVNFYPLGGDPRVLAGYMARNKGLIPS------------------------------   210
UGT82A3    IQVTAGMFGIPTAGFWPAMLSSYLLIASIPHMLQTRLISDTGVP------------------------------------   154
UGT83G4    LEVGF-NLGIKGVLLWTASATSLAYCYSIPKLIDDGIMDSAGHMNPLMQFSHILAKHGCKITFLTSDENYNKMKTTSIIG   202
UGT83G3    LEVGH-KLGIKGALLCPASATSLACAVCIPRLIEDEIIDSEG--------------------------------------   161
UGT84F2    CDVAA-EHEIPSALLWIQSSAVLAAYYNYSNK----LVRFPTET------------------------------------   167
UGT84F3    CDVAT-EHKIPFAILWSESSAVFTAYYNYFHK----LARFPSKP------------------------------------   167
UGT84H1    IDTVA-QHEIPCAMLWIQASALYSIYYHYFKN--NDIFPSLEDP------------------------------------   151
UGT85H3    IQAAE-ELSLPNVLYFPASACSLMCILHFRSFVEKGLTPLKD--------------------------------------   175
UGT85H5    MQVAE-ELALPIVLLFPSSAFTFLSVLHFKTLIQKGLIPLKD--------------------------------------   173
UGT85H4    IQAAE-ELALPIALFVPANACSLLCVFYLHSFVERGITPLKD--------------------------------------   175
UGT85H6    IQASE-ELSLPIILFSPANACNFLCSLHLPTLFEKGLIPLKD--------------------------------------   175
UGT85H7    IQASE-ELSLPIILFSPSNACNFLCGLHLSTLFEKGLIPLKD--------------------------------------   175
UGT85J2    VKASQ-QFGLPNVLFWTHSACGFMSFKECKNLMDRGLIPLKD--------------------------------------   173
UGT85K8    ARVAK-ELGIPELKFWTASTCGFLGYLQFDELVKRAILPFKD--------------------------------------   171
UGT85K9    GRVAK-DLGIPELQFWTASTCGFLGYLQFDELVKRAILPFKD--------------------------------------   170
UGT85X1    LKVAQ-EFNIPEFMLFTPSGCGMLGYLNFEELQKRGYFPLKD--------------------------------------   173
UGT87E3    VVVGN-RRNIPVASLWTTSALIFSVFLHHHLLEQNGHYPVKFSE------------------------------------   162
UGT87H3    VAVAR-RRNIPVALLWTMSSSFYLMMHRLGSFALNRRLEVNLL-------------------------------------   164
UGT87H2    VDVAN-RRNILVAAFWTMSASFYSLLHHLDVFSRHHNLTVDKL-------------------------------------   164
UGT88E4    SQVTT-TLEIPTYFYYTSGATVLSMFIYFPTIHQNATKPIK--D------------------------------------   168
UGT88E5    SQVTT-TLEIPTYFYYTSGATVLSMFIYFPTIHQNATKPIK--D------------------------------------   167
UGT88E6    SQVTT-TLEIPTYFYYTSGATVLSVFLHFPTVHQNATKPIK--D------------------------------------   168
UGT88E11   NQITS-TLDIPTYFYYTSGASSLAVCLQLPTIHQNTTKSLK--D------------------------------------   165
UGT88E9    SQVTN-TLEIPTYFYYTSGASLLSLFLHFPTFHQKSTKPIKDFD------------------------------------   165
UGT88E10   SQVIN-TLQIPIYFYFTSSASMLSFFLHFPTFHKNATKPIKDFD------------------------------------   169
UGT88E7    SQVTN-TLKIATYFYFTSAASMLSIALHFPTFHKNATKGIKDFD------------------------------------   164
UGT88E8    SQINN-ILDIPTYFYFTSAASMLSLFLHLPTYHKKATKAIKDFN------------------------------------   164
UGT88F9    MEIAS-AIGIPVYYFFTSGAAVLSLYSYFPEIHTQTTASFKD-M------------------------------------   163
UGT89F1    HHLAR-DLHVPHLVFSPSGAFAFSVTVSMWRLLPQNDKPEDPNLVVSLPN------------------------------   161
UGT89D5    HQLAT-RLSIPRIAFYSSGALLTSVINYCWKD-PLLFQSR---EVTNFAE------------------------------   170
UGT89B5    QHLAS-ELNIRRLVFSPSAAFAFSTMCFLWKNLPTRVNSKDQNEVVFYHN------------------------------   181
UGT91H7    PQIAR-SMNISCAYFSPCPAWSICFFDTPKQQLGDVESIRKNDEDYYGPP------------------------------   177
UGT91H8    APIAK-SLNISCAHYNITPAWNKCFFDPPKDQVKSSFKL----EDMCGPP------------------------------   180
UGT91M1    VEIAQ-EFHVKLIYYSVVSAATLVFLGPPSNTMKARFSP----ESLTAPP------------------------------   169
UGT92A6    STVAK-ELGLFHVVFSGCSGYGLACYYSLWMNLPHRVTNS---DEFLLPD------------------------------   175
UGT92G3    TNVAK-CLGTKNITFTTCGAYGTLAYISIWSNLPHRKTDS---DEFWVEG------------------------------   185
UGT93A5    VQDAANIENVENYTFHSTCAFTVFSFFWEKMGK-----------------------------------------------   171
UGT95B1    NDVFK-KFDVPTVAFFTSGACSAAMEVAIWKAHPLDMKPDEIRFLPGLP-------------------------------    93
UGT71T2    IDVAV-ELAIPCYLFFASPASFLGFMLHLP--------RVELIE------------------------------------   151
UGT72AB2   HEVAK-QFNLSSYIFYASGAVSLSFLHTLPNLDEKLSSEVKSLES-----------------------------------   113
UGT73C16   SQIAQ-KHQIPRISFHGFSAFCLHCLLKIHTSKILESVNS-ESEYFKVPS------------------------------   155
UGT73P6    VESAA-KLGIPRLYYYSSSYFSSCAAHFIKKHKPHENLIS-DTQIFSIPG------------------------------   157
UGT73P8    VEVAE-KLGIPRIMFHGASYLARSAAHAVEQYAPHLKTQS-DTEKFVIPN------------------------------   151
UGT74X2    LDVAK-RFGIVGVAFLTQNIGIHSIYYHAYMG----KLRVPLDV------------------------------------   134
UGT74Z1    LDVAK-EFGLLGAAFFTQMSGVNYIYYYVHHG----LLKLPIS-------------------------------------   111
           .......170.......180.......190.......200.......210.......220.......230.......240
```

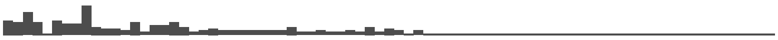

```
UGT708B1    --------------------------------------------------------------------------------   167
UGT71S1     --------------------------------------------------------------------------------   163
UGT71S2     --------------------------------------------------------------------------------   164
UGT71S3     --------------------------------------------------------------------------------   158
UGT71G2     --------------------------------------------------------------------------------   169
UGT71G3     --------------------------------------------------------------------------------   167
UGT71T1     --------------------------------------------------------------------------------   150
UGT72V1     --------------------------------------------------------------------------------   165
UGT72Y1     --------------------------------------------------------------------------------   163
UGT72B17    --------------------------------------------------------------------------------   166
UGT72W1     --------------------------------------------------------------------------------   163
UGT72Y2     --------------------------------------------------------------------------------   163
UGT72B18    --------------------------------------------------------------------------------   164
UGT72AB1    --------------------------------------------------------------------------------   164
UGT72Z1     --------------------------------------------------------------------------------   166
UGT72AA1    --------------------------------------------------------------------------------   160
UGT72L3     --------------------------------------------------------------------------------   164
UGT72L5     --------------------------------------------------------------------------------   169
UGT72X1     --------------------------------------------------------------------------------   164
UGT72L4     --------------------------------------------------------------------------------   171
UGT73K2     --------------------------------------------------------------------------------   175
UGT73AA1    --------------------------------------------------------------------------------   185
UGT73AB1    --------------------------------------------------------------------------------   180
UGT73C15    --------------------------------------------------------------------------------   182
UGT73F5     --------------------------------------------------------------------------------   170
UGT73F7     --------------------------------------------------------------------------------   163
UGT73F6     --------------------------------------------------------------------------------   168
UGT73P5     --------------------------------------------------------------------------------   177
UGT73P7     --------------------------------------------------------------------------------   175
UGT73P4     --------------------------------------------------------------------------------   177
UGT73B15    --------------------------------------------------------------------------------   173
UGT73AC1    --------------------------------------------------------------------------------   163
UGT73B14    --------------------------------------------------------------------------------   177
UGT73P9     --------------------------------------------------------------------------------   179
UGT74X1     --------------------------------------------------------------------------------   156
UGT74K2     --------------------------------------------------------------------------------   160
UGT75L7     --------------------------------------------------------------------------------   158
UGT75L8     --------------------------------------------------------------------------------   161
UGT76J2     --------------------------------------------------------------------------------   148
UGT78K3     --------------------------------------------------------------------------------   168
UGT78K4     --------------------------------------------------------------------------------   165
UGT78K5     --------------------------------------------------------------------------------   165
UGT78G2     --------------------------------------------------------------------------------   174
UGT79B21    --------------------------------------------------------------------------------   167
UGT79B22    --------------------------------------------------------------------------------   167
UGT79B27    --------------------------------------------------------------------------------   168
UGT79A5     --------------------------------------------------------------------------------   170
UGT79B26    --------------------------------------------------------------------------------   167
UGT79B23    --------------------------------------------------------------------------------   167
UGT79B25    --------------------------------------------------------------------------------   165
UGT79B24    --------------------------------------------------------------------------------   167
UGT80B4     --------------------------------------------------------------------------------   210
UGT82A3     --------------------------------------------------------------------------------   154
UGT83G4     GKGKIMESHINLVSLPDGVNPQDDRKDVAKVILSTKTTMSAMLPKVIEEINNINCDNKISCIIVTKNMGWALEVAHQLGI   282
UGT83G3     -------------------NP-----------------------------------------------------------   163
UGT84F2     --------------------------------------------------------------------------------   167
UGT84F3     --------------------------------------------------------------------------------   167
UGT84H1     --------------------------------------------------------------------------------   151
UGT85H3     --------------------------------------------------------------------------------   175
UGT85H5     --------------------------------------------------------------------------------   173
UGT85H4     --------------------------------------------------------------------------------   175
UGT85H6     --------------------------------------------------------------------------------   175
UGT85H7     --------------------------------------------------------------------------------   175
UGT85J2     --------------------------------------------------------------------------------   173
UGT85K8     --------------------------------------------------------------------------------   171
UGT85K9     --------------------------------------------------------------------------------   170
UGT85X1     --------------------------------------------------------------------------------   173
UGT87E3     --------------------------------------------------------------------------------   162
UGT87H3     --------------------------------------------------------------------------------   164
UGT87H2     --------------------------------------------------------------------------------   164
UGT88E4     --------------------------------------------------------------------------------   168
UGT88E5     --------------------------------------------------------------------------------   167
UGT88E6     --------------------------------------------------------------------------------   168
UGT88E11    --------------------------------------------------------------------------------   165
UGT88E9     --------------------------------------------------------------------------------   165
UGT88E10    --------------------------------------------------------------------------------   169
UGT88E7     --------------------------------------------------------------------------------   164
UGT88E8     --------------------------------------------------------------------------------   164
UGT88F9     --------------------------------------------------------------------------------   163
UGT89F1     --------------------------------------------------------------------------------   161
UGT89D5     --------------------------------------------------------------------------------   170
UGT89B5     --------------------------------------------------------------------------------   181
UGT91H7     --------------------------------------------------------------------------------   177
UGT91H8     --------------------------------------------------------------------------------   180
UGT91M1     --------------------------------------------------------------------------------   169
UGT92A6     --------------------------------------------------------------------------------   175
UGT92G3     --------------------------------------------------------------------------------   185
UGT93A5     --------------------------------------------------------------------------------   171
UGT95B1     --------------------------------------------------------------------------------    93
UGT71T2     --------------------------------------------------------------------------------   151
UGT72AB2    --------------------------------------------------------------------------------   113
UGT73C16    --------------------------------------------------------------------------------   155
UGT73P6     --------------------------------------------------------------------------------   157
UGT73P8     --------------------------------------------------------------------------------   151
UGT74X2     --------------------------------------------------------------------------------   134
UGT74Z1     --------------------------------------------------------------------------------   111
            .......250.......260.......270.......280.......290.......300.......310.......320
```

```
UGT708B1   -----------------------------------AVAVEVPGIPFSPLPYSSIPPFLIFPTTIRNFIMEDSPNLT----   208
UGT71S1    ------------------------------QMNELTIPTFATSVPIKSLPSSEI------RKEWESFVTSFSKGLR----   203
UGT71S2    ------------------------------QMNELTIPTFSNSVPLKSVPSIEV------LKELEWFSISFAKGLK----   204
UGT71S3    ------------------------------QQDELDIPSFANPVATNTLPTLVL------RKEWESSFIKYGNGLK----   198
UGT71G2    ------------------------------PDNQLLIPGFSNLVPFNVLPDAAFN-----KDGGYFAYYKLAERFN----   210
UGT71G3    ------------------------------PE--LLIPGFSNPVPSSVLPNSAFS-----KDGGYEAYYKLAERFN----   206
UGT71T1    ------------------------------SDTEFEIPSFKNKLPRLVLPNLALNWK--T-EDGYSWFSYHGRRYR----   193
UGT72V1    --------------------------------EPLKIPGC-KPVRPEDLIDSMLD----RNDSQYKESLIVAEKFP----   204
UGT72Y1    --------------------------------EPVKFPGC-VPFYAKDLPVPFKL----RKKIGYKKFLDRANRFH----   202
UGT72B17   --------------------------------EPVKIPGS-TPVHGKDLLYPVQD----RKDDAYKCFLYHAKRYR----   205
UGT72W1    --------------------------------EPLFIPGC-EPVRFEDTLETFVS----PYGPTHEGFVNTVKNIL----   202
UGT72Y2    --------------------------------EPIKIPGC-VPLCINDLPIPFRF----RSNIGYKKFLDRAKRIQ----   202
UGT72B18   --------------------------------EPVHIPGC-IPIHGKDLLDPVQD----RKNDAYKWVLRNAERYR----   203
UGT72AB1   ------------------------------CYETVNIPGVEIPFHVKELPDPILCE---RSSDTYKSLLDLCQKLT----   207
UGT72Z1    --------------------------------EAIQIPGC-IPIHGNDLPEHFQH----RSSLAYDLILQRCKRLN----   205
UGT72AA1   --------------------------------QPIQIPGCAVPIQGTDLPSSFQD----RTSESYKHFLRRSEGIN----   200
UGT72L3    --------------------------------EPVKIPGC-VPIHGRDLVVQAQD----RLSQSYKFLLKRVERFR----   203
UGT72L5    --------------------------------EPIKIPGC-IPLHGSDLLTPAQD----RSSQAYKHFLQHSKSLC----   208
UGT72X1    --------------------------------EPIKLQGC-VPIHGRDLPAPTKM----RSNDAYKKFLQRAKSMY----   203
UGT72L4    --------------------------------KPIQIPGC-IPLHGRDLSTTIQD----RSSQLYKFKLQRVKKFH----   210
UGT73K2    ------------------------------------LPHPITLP--IKPSPGFAR-----------LTEPLVEAEK----   202
UGT73AA1   ------------------------------------LPHRIEMRKSQLPMIFKSSTSQNLNA----IRERIRKSEE----   221
UGT73AB1   ------------------------------------MPQRIEITRAQLPGTFVSL--PDLDD----YRDKMNEAEM----   214
UGT73C15   ------------------------------------IPDKIEMTIEQTG-LGLKG--EAWKQ----FNDDMLEAEL----   215
UGT73F5    ------------------------------------LPHPISMN--AAPPKKMHE-----------LLVTLFETVF----   197
UGT73F7    ------------------------------------FPHDIVIN--STPPVGSKS-----------FIDPLLTVAL----   190
UGT73F6    ------------------------------------LPHLITLN--ALPPIALTK-----------FMEPLLEIEL----   195
UGT73P5    ------------------------------------LPHNIEITSLQLEEWLRTR--NEFSD----YLNVIYESEG----   211
UGT73P7    ------------------------------------LPHSIQMTTLQLPDWMRNK--SFATG----YFEEMFESEE----   209
UGT73P4    ------------------------------------LPHNIQITSLQLQEGVMTR--NELSD----YFDAIYESEG----   211
UGT73B15   ------------------------------------LPGEITLTKMQLPQVPQHD--KVFAQ----LLEESNESEL----   207
UGT73AC1   ------------------------------------LPDRIEMTKSQVP-IFMRN--TSQIP------DRIKQLDE----   194
UGT73B14   ------------------------------------LPGNIKMTKLQLPSIFTQN--DTMTQNTAKLFAAIRESEK----   215
UGT73P9    ------------------------------------LPHTIEMTPFQLADWIRVK--NSATS----VFDVIFESEK----   213
UGT74X1    -------------------------------QNEISLPLLPT-LEIGDMPSFFLPKG--QNQVLLDLMVGQFSNID----   198
UGT74K2    -------------------------------KSDYLLPGLPK-LHQSDLPSFLYKYG--SYPGYFDIVVNQFANIG----   202
UGT75L7    --------------------------------VSIKLPGLPL-LSARDLPSFLLDTCPSSYALMLSFFEEQFKELDVE--   203
UGT75L8    -------------------------------TCNIELPGLSFSLKSRDLPSFLQASN--IYTFILSSMKEQFRILDEE--   206
UGT76J2    ------------------------SRLEEP---------VVDLPPLKVKDLPVFQ--SQEPEAFYKLVCRFVDECK----   189
UGT78K3    ----------------------------------LDILPGLSNMRVGDLPQDVTDGGED-EPLFSKTLALLGKVLP----   209
UGT78K4    ----------------------------------LDFLPGLSVLRVEDLPHDILMTGTEKENVLTRALDSLSKILP----   207
UGT78K5    ----------------------------------LDFLPGLSVLRVEDLPHDTLMKGTDKENDLTRALASLSKILP----   207
UGT78G2    ----------------------------------VDLIPGFPELKVCDLPEGVID---DTDGPFATMLHKMGLELP----   213
UGT79B21   -----------------------------------SFP--PSAIKLQPHEARGLATSTVNGYGKDISFMERQLIAF----   206
UGT79B22   -----------------------------------SFP--PSAIKLQPHEARGLATSTVKGYGKDISFMQRQLIAF----   206
UGT79B27   -----------------------------------GYP--DSSIKLFQHEAKSFAAKRTEYFGSNVLFYDRQSIAL----   207
UGT79A5    -----------------------------------GYP--KNNIFLKTFEAMDFMFL-FQKFTEDITGYERALQSL----   208
UGT79B26   -----------------------------------GYP--DSSIKLHSHELQFLASSRKLVFGSGVLLYDRLHFGT----   206
UGT79B23   -----------------------------------GFP--DTCINLHSHELRFLVSSRKLEFGSGILLYDRVDLGA----   206
UGT79B25   -----------------------------------GFP--DSCINLHSHELRFLVSFKKLEFGSGVLFFDRLESGS----   204
UGT79B24   -----------------------------------GFP--GSCIKFHSYELRYLASIGNVVFGSGVLLMYRLDTGT----   206
UGT80B4    --------------------------------GPTEISIQRKQLKTIIDSLLPACTAPDSETGIPFTAQAIIANPPAYGH   258
UGT82A3    -------------------------------QHEGKINFVPELPVVSTNDLPWLIGTIGARKERFKFWMRTLERSI----   199
UGT83G4    KGALFWPASATCLVSFNSMESLVEEGIIDSQNGLPKKEEIQLSTNLPMMEAAAMPWYNLHNAFFFLHMMKEMQNMN----   358
UGT83G3    ----------------------------------TKQQEIQISPDIPMIDTTNLPWHG-ADKIFFDYIVQEMQTIN----   204
UGT84F2    -------------------------------EPYIDVQLSSVVLKYNEIPDFLHPFS--SYPFLRALILEQIKNLS----   210
UGT84F3    -------------------------------EPYIDVQLCSLLLKHNEIPDLLHPFN--NYPFLGTLILEQIKNLS----   210
UGT84H1    -------------------------------NETVQLPGLPI-LEVRDLPTFILPSYP---LHFKEVVENSFKDLE----   192
UGT85H3    ------------------------ESYLRNGFLEAKVEWIPGLKNFRLKDIVAFIRTTNPNDIMLEFLIEMADRVN----   227
UGT85H5    ------------------------ESYLTNGYLDTKVDWIPGLRNFRLKDLPDFIRTTDPNDLMIEFVIEVADRFH----   225
UGT85H4    ------------------------ESYLTNGYLETKVDWIPGLKNFRLKDINDFIRTRDPNDIMLGFFSAVADRIP----   227
UGT85H6    ------------------------KSYLTNGYLETKVDCIPGLKNFRLKDLPDYVRITDPNDSLVEFIIESIDRAP----   227
UGT85H7    ------------------------KSYLTNGYLETKVDCIPGLKNFRLKDLPNYIRITDPNDSLVEFVIEKMDRAH----   227
UGT85J2    ------------------------ASYLTNGHLDTIIDWIPGVKNITLRDLPGIYHTIDPNDLLLNFVVEQIEVAS----   225
UGT85K8    ------------------------EKFIVDGTLETNLDWVIGMKDMRLKDLPSFIRVTDLNDIMFDFLRSESKNCL----   223
UGT85K9    ------------------------ENFVADGTLDTSLDWISGIKDMRLKDLPSFMRVTDLNDIMFNFMGSEAQSCL----   222
UGT85X1    ------------------------EKNLCDGYLETEVDWIPAMRGARLKDLPTFFRTTNSSDTMFNYNKDTVNNAM----   225
UGT87E3    -------------------------------NGDKRVDYIPG-ISSTRLADFPLNDDSSKSKRMMQMSLKGFKWIH----   206
UGT87H3    ---------------------------------DDYGEHIQG-ISSSQLADLRVMLHQN-DLRFLQLGLECLSTVP----   205
UGT87H2    ---------------------------------DEKVENILG-VSSLYIQDIRTVLRKT-DDRALQLALECIFNVQ----   205
UGT88E4    ------------------------------LHMPLQIPGLPKNISTDDYPDEAKD----PETKAYKVLLDSAKTVR----   210
UGT88E5    ------------------------------LHMPLQIPGLPKNISTDDYPDEAKD----PETKAYKVLLDSAKTMR----   209
UGT88E6    ------------------------------LHMPLQIPGLPMNISTDDYPDEAKD----PEIKVYHVLLDSAKTMR----   210
UGT88E11   ------------------------------FHMHPKIPGLPM-ISIEDMPEEVKD----RENKGYKVFLDIARSMK----   206
UGT88E9    ------------------------------IHTPIDLPGLPR-LSVDDYPNEAKD----PSSQSYHILLESAKSLR----   206
UGT88E10   ------------------------------IHTPIELPGLPR-LFVDDWPEEAVD----PTSPSYQMLLECAKSMR----   210
UGT88E7    ------------------------------IDTPIEIPGLPT-LFVDDYPDDGKD----PTSPGYQILLECTKAFR----   205
UGT88E8    ------------------------------IDTPIELPGLPT-LFVDDYPDDGKD----PTSPGYQTLLECAKVFR----   205
UGT88F9    ------------------------------AGVQIHAPGNAA-LNATLMPDPVLD----RGDPAYWEMLYFCEHLS----   204
UGT89F1    ------------------------------------LPNSPVYPWWQISHLFRDNR-GEPDW---EIIKNDFLLNL----   197
UGT89D5    ------------------------------------IHGMPSFPRDHLPSLFRRYIESDPES---EFIKESFVSNS----   207
UGT89B5    ------------------------------------IPSSPKYPWWQVSPMYRSYVAGDPDS---DKLRDWFLCNI----   218
UGT91H7    -----------------------------------KWIPFPTKIGLRSYEVKKLLEDVKVNETGASPVFD-LNKTN----   217
UGT91H8    -----------------------------------KFVPFETTIHLKPYEIIRAFTALK-DESGGMAGFD-LNKAY----   219
UGT91M1    -----------------------------------EWVTFPSSVAYTRNEAIAFSAIVHQKNASEVSGIERLVKVI----   210
UGT92A6    ------------------------------------FPEARLIQRNQLPNNILQADGTDPWS---IFQHKNLSDWV----   212
UGT92G3    ------------------------------------FPQNYRFHISQMHRYLRQADGSDSWS---KFFPPQIALSM----   222
UGT93A5    ----------------------------------------PPLQTSHILQFPSLEGCFIPQIIDFIAAQKEFLKLN----   207
UGT95B1    --------------------------------EDMAVTYSDLKRRHHDHHPPPPPRGLGPRKMGPPKLGEQPSWLDEI--   139
UGT71T2    ------------------------------SETEFEIPCFKNPLPRLVLPNMVLNWK--TEEDGYSWVSYHARRYR----   195
UGT72AB2   ------------------------------AYETLNIPGSVIPYHIKDIPDPLLCE---RSSDVYKLFLEVCTEFS----   156
UGT73C16   ------------------------------------IPDKIQVNKQQIP-ETLS---DELKD----FGEQMQDAEI----   187
UGT73P6    ------------------------------------LPHNIQITSLQLQEGVRTR--NEFSD----YFDAVYESEG----   191
UGT73P8    ------------------------------------LPDELMMTRLQLPDWLRSH--NQYTD----LMKVIKESER----   185
UGT74X2    -------------------------------KENISLPMLPQ-LQYRDMPSFFHTYE--KDPTFLHLCLDQFSNMD----   176
UGT74Z1    -------------------------------SMTISIQGLPL-LDIKDTPSFVNDPG--FYPAYYELVMNQFSNIH----   153
           .......330.......340.......350.......360.......370.......380.......390.......400
```

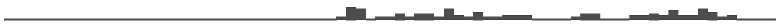

```
UGT708B1   ----NLDGVFANTFEALESYSLETLNSGKVVKNLP-----PVYAVGPFVSFEFEKESQQ-----------------TALT   262
UGT71S1    ----KADGIIVNSFEELESYAVQAFLNDRDLASL------PIYPVGPILNPESETKSTVD---------------FDDVI   258
UGT71S2    ----KANGIIVNSFEELEPYAVRSFLNDIDLAAI------PIYPVGPILNPDSKTKATAD---------------FDDVI   259
UGT71S3    ----KASGIIVNSFHELEPHAVRSFLEDPTLRDL------PIYPVGPILNP----KSNVD---------------SDDVI   249
UGT71G2    ----DTKGIIVNTFSDLEQYAIDALYDHDEKIP-------PIYAVGPLIDLKSQTNPNLDQ------------AQHDLIL   267
UGT71G3    ----NTKGIIVNTFSELEKYAIDALYDHYDRTP-------PIYAVGPLIDLKSQPNPNLHQ------------AQRDLIL   263
UGT71T1    ----ETKGIIVNTLQELEPHALQSLHNDLQLP--------PVYSIGPIVDHVGPVQWDPNP------------AQYDYIM   249
UGT72V1    ----ESDAVLVNTWDELQQREVKALTN-GELSGVL--K-VPVFAVGPLVRQPE-SETGQL------------KKP-VII   261
UGT72Y1    ----LSDGFFVNSFVEFEEEAVKALKEVSKEKKP------LVFCVGPIIQKGS-IFGEEN-------------GLGLECL   258
UGT72B17   ----LAHGIIENSFVELEPEVIKLLQINEPGRP-------LVYPVGPLVNEDSSRTGVDG-------------VEIDKCL   261
UGT72W1    ----TADGILMNTWHDLEPKTIKAVSEQGILGRFT--K-GSVYSVGPLVRTVE-PELKRE-------------ENGNLIL   261
UGT72Y2    ----LPDGVFVNSFVELEEEAVKALKEESKEKKP------MVFCVGPIIQKGS-IFGEEN-------------GLGLECL   258
UGT72B18   ----EADGIIENSFVELEPGPIKELQKEEPGKP-------PVYPVGPLVNKDVAQTGSN-----------------SECL   255
UGT72AB1   ----LFDGVIINTFTDLEQDVIRVLQER--EKL-------CVYPIGPIIRKES---NN-E-------------TNMEVCL   257
UGT72Z1    ----LADGFLVNSFMKMEENTLEALEEQNREKHNG-----SVFFVGPIIQNGTNNESKGS-------------DSDLECM   263
UGT72AA1   ----LCDGILVNSFVEFESKAVKTMMEKSINGDKP-----HVYMVGPIVQKNCDNTQ-----------------NGKECL   254
UGT72L3    ----LVDGVIINSFLEMEIGVIRALVEEGSGNP-------VVYPVGPIIQQDT-QQG-----------------HDLECL   254
UGT72L5    ----FADGVLVNSFLEMEMGPIKALTEEGSGNP-------AVYPIGPIIQTGT-KSGSDV-------------GNGKECL   263
UGT72X1    ----FFDGILCNSFLELESGAIKALEEKGHHENGK--K-ISIFPVGPITQKGS-SASSND-------------VDEFECL   262
UGT72L4    ----FVDGIFVNSFLEMEMGPIKTLTQEGSGNP-------VVYPVGPIIQQDT-QHG-----------------NNIECS   261
UGT73K2    ----GSHGVIVNSFAELD-EGYTEYYENLTGRKVWHVG-----PTSLMIKT-TLEKTDNI---------SNGSSTKHKCL   262
UGT73AA1   ----QAYGVVVNSFEELE-DGYIEEYKKVTGHKVWCVG-----PVSLSNKD-YLDKAQRGS--------NNLIDNANEYV   282
UGT73AB1   ----CSHGIVVNSFEELE-QGCAMEYEKVMNKRVWCIG-----PLCLSNKE-SLDKFERG---------NKSSIEEKQCL   274
UGT73C15   ----GSYGIIMNSFEELE-PAYAREYKKVRNEKVWCIG-----PVSLSNTN-YLDKVQRGNN-------NKVSIGEWKHL   277
UGT73F5    ----KSNGIVVNNFVELDGEEYIKHYEKTTGHKAWHLG-----PASLIRKT-DQEKAERG---------EESVVSVHECL   258
UGT73F7    ----KSHGFIINSFVELDGEEYVEYYEKTMTHKAWHLG-----PASLVRRT-TQEKADRG---------EKSTVSVEKCL   251
UGT73F6    ----KSYGLIVNNFSELDGEEYIEHYEKTTGHRAWHIG-----PVSLICRT-TEEKAERG---------QTSAVSVHECM   256
UGT73P5    ----RSYGTLYNSFHELE-SDYEQLYKSTMGIKAWSVG-----PVSTWVNKDDVEKARRG--------HSKDLAVESELL   273
UGT73P7    ----RSYGTICNSFHELE-SDYEKLCKTIMGIKCWSIG-----PVSAWANKDDDEKKANRGH-----MEINNIGNEQEWL   274
UGT73P4    ----RSYGTLYNSFHELE-SDYEQLYKNTMGIKAWSVG-----PVSTWINKGDVQKR-----------HNEDLVVESKLL   270
UGT73B15   ----KSYGVIANSFYELE-PVYADHYRNELGRKAWHLG-----PVSLCN-RETEEKACRG---------RESSIDEHECL   267
UGT73AC1   ----KSFGTVINSFYDFE-PAYADYTRNVLGIKAWLVG-----PVSLCN-RSVEDKKERG---------KQPTMDEQSCL   254
UGT73B14   ----KSYGVIVNSFYELE-HVYADYYRDVLGIKAWHIG-----PLSIHNKKDKEITSYRG---------KEDSIDKHECL   276
UGT73P9    ----RSYGTIYNSFHELE-SDYEKLGRTTIGIKSWSVG-----PVSAFTNNDDEKKD-----------NKGHIGKEAEWL   272
UGT74X1    ----KADWILCNSLYDIEKKVVDWTMKIWPK--F--------RSIGPSIPSMFLDK--RLKDD-EAYGATQFKSN-EECM   260
UGT74K2    ----KADWILANTIYELEPEVVDWLVKIWP---L--------KTIGPSVPSMLLDK--RLKDD-KEYGVSISNPNTEVCI   264
UGT75L7    ---TNPTTILVNSFEALEPDALR-AVEN-LN--M--------ISIGPLIPSAFLDG--KDPTD-NSFGGHIFQP-SNNCV   264
UGT75L8    ---TNP-IVLVNTVDEFELESVR-AIDDKIK--M--------IPIGPLIPSAYLDG--KDLTD-TSFGGDVIRVDSEDYI   268
UGT76J2    ----RSSGVIWNTFEEFESSALAKLRQDFSIPMY---------PIGPFHKYLLPSS-----------TSTSLLTPDKSCI   245
UGT78K3    ----QAKAVVMNFFEELDPPLFVEDMRSKLKCLL--------YVGFLTFSLPLPPL-------------PPSESDETGCL   264
UGT78K4    ----EAKTVVVSFFEELDLPLFVEDIRTKLKSML--------YV--PLFNLKPKTT-------------PQNEIDESGCI   260
UGT78K5    ----EAKAVVVSFFEELDLPLFVEDIRTKLQSML--------YV--PLFNLKPKTT-------------PQNQIDESGCI   260
UGT78G2    ----RATAIAINSFATIHP-LIENDLNSKFKLLL--------NVG----PFMLTTP-------------QPLISDEQGCL   263
UGT79B21   ---TSCDAIVFKTCREMEGLYCDYLERQMRKQV---------FLAGPVFPNPPTSTLKE------------------KWV   256
UGT79B22   ---TSCDAIVFKTCREMEGLYCDYLERQMRKQV---------FLAGPVFPKPPTFTLEE------------------KWV   256
UGT79B27   ---NEADALGYRTCREIEGPYLDYIQKVFNKPV---------LASGPVILEKPNSCLEE------------------KGT   257
UGT79A5    ---SECSYVVFKSCKEMEGPYIDYIEKQFGKQV---------LSVGPLVPEPPMDVLEE------------------KWL   258
UGT79B26   ---SLADAIGFKGCKEIDGPYAQYLGTVYGKPS---------LLSGPLLPESPLTTLDE------------------KWV   256
UGT79B23   ---RLADVIAFKGCKEIDGPYVEYLGSVFGKSV---------LLSGPILPEPPMSTLEE------------------KWV   256
UGT79B25   ---KLSDAIAFKGCKEIDGIYADYIEMVLRKPV---------FLSGPLLPEPTLSSLEE------------------KWV   254
UGT79B24   ---SLADAIGYKGCKEIDGPYVEYLRTIYGKPV---------LLSGPLLPEPTLSSLEE------------------KWV   256
UGT80B4    VHIAEALGVPIHIFFTMPWTPTYAFPHPLARVPQGAGYWLSYIIVDLLIWWGMRGIINSFRKRTLKLPPIAYFSMYRGSI   338
UGT82A3    ----KLKWILVNSFPNETKVGLQPSSSNSQHVLP----------IGPICRPHELTKTLS------------FWKEDLSCL   253
UGT83G4    ----LGEWWLCNTSMDLEAEAISLSPKFL--------------PIGPLMGNEHNIM-------------GSLWQEDETCL   407
UGT83G3    ----LAEWWLCNTSYNLEPGSISISPKFL--------------PIGPLIENNNNNK-------------SSFWKEDTTCI   253
UGT84F2    ----KVLYILVDSYEELEHEFIDYLSKKSIF--I--------KPVGP----LFNNP--KAKDA-SNIPSDFVKSNDCDTI   269
UGT84F3    ----KALCVLVDTYEELEHDFIDYISNKSVV--I--------RPIGP----LFNNP--KIKCA-SNIRGDFVKSDDCNII   269
UGT84H1    ----KVKWILGASFFEIEKEIVESMDSLTP---I--------YPIGPLVSPFLLGE--KDTND-LSLDMWNAEDS---CI   251
UGT85H3    ----RESTIVLNTFNELESDVIDALSSMFPSLY----------PIGPLPSLLNQTP--QNH---LASLGCNLWKEDTKCL   288
UGT85H5    ----KASAIVFNTSNELESDIMNVLDSMFPSLYT--------IHIGPFASFLNQSP--QND---LTSLDSNLWKEDTKCL   288
UGT85H4    ----RDTTIVLSTSNELESDVINALSSMFPSLY----------PIGPLPSLLNQTP--HNH---LTSLSSNLWKEDTKCL   288
UGT85H6    ----RAAAIIFNTSNELENDAMNALSSMFPSIY----------AIGPLSSYLNESL--QNH---LVSLSSNLWKEDTKCL   288
UGT85H7    ----RAAAIIFNTSNKLENDAMNALSSMFPSIY----------AIGPLSSFLNESL--PNH---LVSLSSNLWKEDSKCL   288
UGT85J2    ----KASAIILPTFDALESDVLNALSTMFPKLY----------SIGPLEFFLDQIS--DNR---FESFHCNLWKEEFECL   286
UGT85K8    ----SSSTIIINTFEELESEALETLKAQNPNIY----------SIGPLHMLGRHFPEKENG---FKASGSSFWKNDTECI   286
UGT85K9    ----RSSTIIINTFEELECEALETLRAQNPNIY----------SIGPLHMLGRHFPEKENG---FKASGSSFWKNDTECI   285
UGT85X1    ----KAKGIILNTFQELEAEVLDAIRIKYPHLY----------PIGPLSMLHKKFSISKNNNQ-LESIDFNLWKEDVKCM   290
UGT87E3    ----KAQYLLFTSIYELEPQAIDIIKSKLSLP-I--------YTIGPTIPYFSLNK--KNNLK-TN-------AYHS-YI   262
UGT87H3    ----KADCLIVNTIQELEAEEIKSLKTLLNFP-I--------YPI--AFPYFKHET--SHSVT-YS-------DSKPDYI   260
UGT87H2    ----KANYLLLTTVQELEPETIDSLKAILPFQ-V--------YPIGPAIPYLELED--KNSPK-TD-------HSHDYIL   262
UGT88E4    ----ECDGIIVNTFDAIEEKTIKSLKEGLFVPDG--IT-PPIFCIGPLITASYGEDENG-------------------CL   264
UGT88E5    ----ECDGIIVNTFDAIEEKSIKSLNEGLFVPDG--IT-PPIFCIGPLITASYGEDENG-------------------CL   263
UGT88E6    ----ECDGIIVNTFDAIEEKAIKSLNEGLFVPDG--IS-PQIFCIGPLITTPYGGDEN----------------------   261
UGT88E11   ----ESDGVIINTFDAIEERVLKALKGGLCLPEG--TT-PPLFCIGPMISPLCKVEDERG----------------SV-CL   263
UGT88E9    ----ESDGIIVNTFDELERKAIKALRDGLCVPEG--IT-PMIFCIGPVLSSLYE-DENI-------------------CM   259
UGT88E10   ----ESDGIFVNSFDAIERKVIKAMRDGLCVPDG--TT-PLIFCIGPVISALYEKDENR-------------------CM   264
UGT88E7    ----ESDGIIVNTFEAFESKAIKALKDGSCVPDG--TS-PLIFCIGPVISTLYDTDEHQ-------------------CM   259
UGT88E8    ----ESDGVIINTFDAFESKAIKALRDGSCVPDG--TS-PLIFCIGPVISTLYDTDEHQ-------------------CM   259
UGT88F9    ----MAKGIVVNTFCELEDVAVKAVEDGACFPDLKRTP-PSVYCIGPLIADAQQSDEARH---------------IKDCL   264
UGT89F1    ----DAWGVVFNSFVDLETDYFDHLKKELGHERVWTVG-----PVLPLESGPEERGGSST-------------VPCNDLT   255
UGT89D5    ----LSWGFVFNTFRALEGPYLDQLEAQFGNRRVFAVG-----PLGYSRVKSDQDEGS-------------------EVL   259
UGT89B5    ----QSWGLIVNSFDELEKPYLDYLKTELDHNRVWAVGL--LLPVDNFSAMAIQRGGSSS-------------ILVNDVV   279
UGT91H7    ---SGCDMFVIRSSRDLEPEWLDFLGEFYNKPV---------IPVGLLPPSMQISDFDEVDN----------SPDWIQIK   275
UGT91H8    ---SSCDLFLLRTSRELEGEWLDYISDRYEVPV---------VPVGLLPPSMQIRDDEEEEN----------DPDWVKIK   277
UGT91M1    ---GAAKAVICCSCYEIEGEYLHLYNKLVQKPV---------IPIGLLPVEMPQREIFEESR----------SE--SKIF   266
UGT92A6    ----NSDGILFNSVSEFD-SVGLSYFTRKLQIPVWSIG-----PVVLST----GTGSR----------GKGGGINPKTCL   268
UGT92G3    ----KSDGWICNTVEKIE-TFGLQHLRKYLELPVWSVG-----PLLPPSSALKGLNSKYRA-------GKESGIALEECI   285
UGT93A5    ------DGNIYNTSRAIESTYMELIEKITCSKKH--------WAVGPFNPLTIEKKDSKG---------------RHSCI   258
UGT95B1    ---RETIGLMINTCDDLERPFIDYIANHVGKPVWG---------VGPLLPEQYWKSFGSVIHDRDFRSNRLSSVTEEEVV   207
UGT71T2    ----EAKGIVVNTLEELEPYALQSLNNDLQLP--------PVYPIGPILDLVGPVQWNPNP------------VQYNYIM   251
UGT72AB2   ----LVDGVIINTFTDLEPEVIRVLQEK--KKP-------FVYPVGPIIRKES---NNNK-------------DNILMCL   207
UGT73C16   ----KSYGVIINTFEELE-KSYVEDYKKERNGKVWCVG-----PVSLCNKDGTLDKAQRG---------NMATISENNCL   248
UGT73P6    ----RSYGTLYNSFHELE-SDYEQLYKNTMGIKAWSVG-----PVSTWINKGDVQKR-----------HNEDLVVESKLL   250
UGT73P8    ----KSFGSVFNSFYELE-SDYFDHYKRVMGTKSWGLG-----PVSLWVNQDDSDKAARG-Y-----SKEEEEEKEEGWL   249
UGT74X2    ----KADWILCNSFYELHQEVADWTMNIWPN--F--------RTIGPSIPSMFLDK--RIKYD-EDYGAPQFKSE-EYCM   238
UGT74Z1    ----KADVILVNSFYKLEDKVVDSMSKLCP---I--------LTIGPTVPSFYLDK--EIPND-KDNDLNLFQLD-SSAI   214
           .......410.......420.......430.......440.......450.......460.......470.......480
```

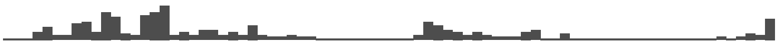

```
                                             G
                                           . ↓
UGT708B1    KWLDGQPIGS--VVYVCFG--SRTALGRDQMREIGNGLIRSG-YKFLWVVKDKIVDKEEEIG--------------LDEI 323
UGT71S1     KWLDDQTPSS--VVFLCFG--SRGSFDEDQVMEIAHAIENSG-AHFLWSLRKPPPKGVRAAPTD--YPLS-----ELKSI 326
UGT71S2     KWLDAQPPSS--VVFICFG--SRGSFEKNQVMEIARAIENTG-VRFLWSLRKPPPKGFTIAPID--YHLS-----DLTSI 327
UGT71S3     KWLDDQPPSS--VVFLCFG--SMGTFDEEQVREIALAIERSG-VRFLWSLRKPQPQGTMVPPSD--YTLS-----QMLEV 317
UGT71G2     KFLNEQPDKS--VVFLCFG--SMGSFVLSQTREIALGLKKSG-VRFLWAMRSP-PTSNNEG-----------------KS 324
UGT71G3     KWLDEQPHKS--VVFLCFG--SMGSFGSSQTREIALGLKHSG-VRFLWAMRSP-PTSNNEE-----------------KI 320
UGT71T1     KWLDMQPLAS--VVFLCFG--SMGSLEAKQVEQIAIGLERVG-VRFLWALREP-PKTRLEGPRN--YTS-------HEDV 314
UGT72V1     QWLDKQPRES--VIFVSFG--SGGTVSYEQMKEIAFGLELSE-QRFIWVVRASTGGGVDTAFFTTGSSSFNDELDEIRKH 336
UGT72Y1     KWLEKQEPKS--VLFVSFG--SGGTLSQEQFNELAYGLELSG-QKFIWIVKEPNGVAN-ASYFGGEIE-------DPLNF 325
UGT72B17    SWLDEQPRGS--VIFVCFG--SGGTLSSAQMNELAIGLETSE-QRFLWVVRCPKDKVANVSNFIADSN------VDPFDF 330
UGT72W1     SWLDRQPAES--VIYLSFG--SGGTMSERQMRELAYGLELSQ-QRFVWVVRRPIEGDASAAFFERVDG------SAVVDY 330
UGT72Y2     KWLEKQEPKS--VLYVSFG--SGGTLSQEQFNELAYGLELSG-QKFLWVVREPSGVAN-ASYFGGEIK-------DPLKF 325
UGT72B18    KWLDGQPHGS--VLFVSFG--SGGTLTSNQIIELALGLEMSE-QRFLWVVRSPNDKVANASYFSADTQ------ADPFDF 324
UGT72AB1    RWLENQPPSS--VLFVSFG--SGGTLSYQQLHELAFGLEISG-CKFLWVVRVPSKNSS-SAYFSKQND-------DPLEY 324
UGT72Z1     KWLENQIPNS--VLYVSFG--SGGTLSQEQLNELAFGLELSG-QNFLWVLRAPSDTSN-EAYLGVKNN------DDPLNF 331
UGT72AA1    SWLDEQSQSS--VVFVSFG--SGGTISQKQMNELAYGLELSS-QKFLWVLREPKDIAS-ANYFGVSSIQ-----EDPLSF 323
UGT72L3     AWLDKQQPCS--VLFVSFG--SGGTLSQEQIFELALGLELSD-HRFLWVMRAPSNLAN-AAYLSGGKDG-----VDPLQF 323
UGT72L5     TWLDKQKPCS--VLYVSFG--SGGTLSQEQTVELALGLELSN-HRFLWVVRAPNNSAN-AAYFSTQDDD-----VDPLKF 332
UGT72X1     KWLKNQPQNS--VLYVSFG--SGGTLSLRQINELAYGLELSD-QRFLWVLRAPSDSVS-ADYFEDAN-------VDPLKF 329
UGT72L4     AWLDKQKPRS--VLFVSFG--SGGTLSQEQIFELALGLELSN-HKFLWVVRAPSSRSKSAAYLSAQNDG-----IDPSEF 331
UGT73K2     TWLDTK-EPSS-VVYISFG--SLCSLSNDQLLELAKGIEASK-HQFLWVVHRKGD---DDDDDN-------------ENW 321
UGT73AA1    KWLDSW-PQNS-VIYICLG--SLNRVTPKQMIEIGLGLEASN-RPFIWVVRKA-YRWGELEK----------------WI 340
UGT73AB1    EWLNFR-ETRS-VIYVCLG--SLCRLVSSQLIEIGLGLEASN-RPFIWVVKTNGENYLELEK----------------WL 333
UGT73C15    KWLDSK-EPKS-VIYACLG--SLCNVTPPQLIEIGLALEATK-RPFIWVIREG-NQLEALEK----------------WI 335
UGT73F5     SWLNSK-RVNS-VIYIGFG--SLCHFPDKQLYEIACGIEASG-YEFVWVVPEKKGKEYESEEEK-------------EKW 320
UGT73F7     AWLNSK-RDKS-VIYISFG--TICYFPDKQLYEIASAIEASG-YEFIWVVPEKRGKENESEVEK-------------ENW 313
UGT73F6     SWLNSK-QPNS-VIYICFG--SLCHFSDNQLYEIASAIKASE-HQYIWVVPEKKG----NEDEN-------------EKW 314
UGT73P5     NWLNSK-ENES-VLYVSFG--SLTRLSHAQIVELAYGLENSS-QSFIWVIRKKDKDEDGEG------------------ 329
UGT73P7     NWLNSK-PNDS-VLYVSFG--SLTRLPHLHLVEIAHGLENSG-HNFIWVVRKKDRDEGGNG------------------ 330
UGT73P4     NWLNSK-PDDS-VLYVSFG--SLTKLPHAQLVELAHGLENSR-HNFIWVVRRKD--EDGES------------------ 324
UGT73B15    KWLQSK-QPNS-VVYVCFG--SMTVFGDAQLKEIAMGLEASE-VPFIWVVKKGAKSEGEK----------------SEW 325
UGT73AC1    NWLNSK-KPNS-VLYVSFG--SLARLHTKQIKEIAYGLEASD-QSFIWVVGKILNSTDKEETCG------------ENW 316
UGT73B14    KWLDTK-EKHS-VVYLCFG--STTHFLNSQLKEIAVGLEASG-KNFIWVVNK-KKEDGE------------------EW 331
UGT73P9     NWLNSK-ENES-VLYVSFG--SLTRLSHAQIVELAYGLENSS-QNFIWVIRKKDKDEDGES------------------ 328
UGT74X1     EWLNDKPKGS--VVYVSFG--SMGTLDEEQIQEIAYGLRDSE-SYFLWVVRAA-----------------------EEIK 312
UGT74K2     KWLNEKPKGS--VVYVSFG--SMAGLSEDQTIELAYGLKDSD-TFFLWVVRDC-----------------------EQVK 316
UGT75L7     EWLDSKQENS--VVYVSFG--SLCVLPKKQMEEIARALLDCG-FPFLWVIKEK----------------------EEELN 317
UGT75L8     EWLDSKDESS--VVYVSFG--SFSVLPKKQMEEFARALLDSG-LNFLWVIREKKVDEK-------------K-EDDELS 328
UGT76J2     SWLDKQEPNS--VVYVSFG--SIVAISEAEFLEIAWGLADSN-QPFLWAIRPGVIRG----------------SEWLEP 303
UGT78K3     SWLDKQKDRS--VVYVSFG--TVVTPPPNELIAVAEALEESG-FPFLWSLKDHLK----------------------GL 316
UGT78K4     SFLEVQKAKSLQVVYVSFGT-TVVEPPEHEIVALAEALEEYG-FPFIWSLKENLR-----------------------NL 315
UGT78K5     SFLEVQKAKSLQVVYVSFGT-TVVEPPQHEIVALAEALEEYG-FPFIWSLKENLR-----------------------NL 315
UGT78G2     EWLNQHKNFS--VVYISFGS-TIIPPP-HELIALAESLEECG-FPFIWALRGDPK-----------------------EK 315
UGT79B21    TWLGRFKPKT--VIFCAFG--SECILKSNQFKELLLGFELTK-IPFIAALKLP------------------IGAETIKSA 313
UGT79B22    TWLGGFKPKT--VIFCAFG--SECILKSNQFKELLLGFELTK-IPFLAALKPP------------------IGAETIKSA 313
UGT79B27    YGFNRFEKNS--VVYCCFG--SECTLKPDQFTELVLGLELTG-KPFFAALKPP------------------FGFATVEEA 314
UGT79A5     KWLDNFPAKT--VIFCSFG--SETYLNDDQISELATGLELTG-LPFILVLNFPSN---------------LCAKTELERT 318
UGT79B26    SWLKGFKHGS--VVYCAYG--SEGPLQENQFKELLLGLELTG-FPFVAALKPP------------------VGFESIEEA 313
UGT79B23    SWLNGFKQGS--VVYCAYG--SESALEHNQFKELLLGLELTG-FPFLAALKPP------------------VGFESIEEA 313
UGT79B25    SWLNGFKHGS--VVYCAYG--TESALEHNQFQELLLGLELTG-FPFLVALKPP------------------IGFESIEEA 311
UGT79B24    SWLKGFKLGS--VVYCAYG--SESALEHNQFKELLLGLELTG-FPFLVALKPP------------------IGFESIEEA 313
UGT80B4     SHLPTAYMWSPHVVPKPSDWGSLVDVVGYCFLSLASKYQPRE-DFVRWIKKGPPPLYFGFGSMPLEDPKR------TTNV 411
UGT82A3     KWLTNQKANS--VVYVSFGS-WVNPMGESKLKNLALALEASK-RPFIWVLRSTWR-----------------------EG 306
UGT83G4     EWLDQYPPKS--IIYVSFG--SLISIGPNQFIELAHGLDLLK-RPYLWVVRKDKG---------------------DNEV 461
UGT83G3     DWLDKQAPRS--VIYVSFG--SLAVIDQNQFNELALGLDLLN-KPFLWIVRPNN----------------------DNIV 306
UGT84F2     EWLNSKSPDS--VVYISFG--TIVYLPQEQVDEIAYGLLSSK-VSFLWVLKQPLKELG----------------LKPHV 327
UGT84F3     EWLNSKPLSS--VVYISFG--TIVSLPQEQVNEIAYGLLNSKQVSFLWVLKVKTPPN--------------------EIV 325
UGT84H1     DWLDNRPNSS--VIYISFG--SIIVFSEKQMENIIIALKNSN-KDFLWVFKPANNGGS--------------EKEDSTLE 312
UGT85H3     EWLESKEPRS--IVYVNFG--SITVMTHEQVLEFAWGLANSK-KPFLWIIRPDLV-------------------IGGSVV 344
UGT85H5     EWLETNEPGS--VVYVNFG--SITVMSQEKLLEFAWGLANSK-KPFLWIIRPDLV-------------------IGGSVI 344
UGT85H4     EWLESKEPRS--IVYVNFG--SITVMTHEQVLEFAWGLANSK-QPFLWIIRPDLV-------------------IGGSVV 344
UGT85H6     EWLESKEPKS--VVYVNFG--SMTVMTLEKLLEFAWGLANSK-KPFLWIIRPDLV-------------------IGGSVV 344
UGT85H7     EWLESKEPKS--VVYVNFG--SMTVMTSEKLLEFAWGLANSK-QPFLWIIRPDLV-------------------IGGSVV 344
UGT85J2     KWLDSQEPNS--VLYVNFG--SVIVMRYNQLVELAWGLANSK-KKFLWVIRPDLV-------------------EGETLL 342
UGT85K8     KWLDKWEACS--VLYINYG--SITVMTDHHLNEFAFGIANSK-VPFLWIMRPDVV-------------------MGESFN 342
UGT85K9     KWLDKWEACS--VLYINYG--SITVMTDHHLNEFAFGIANSK-VPFLWIMRPDVV-------------------IGESFN 341
UGT85X1     KWLDERDKGS--VVYVNFG--SLVIMNAKQLREFAWGLSNSK-YNFLWIIRPNLVD------------------CGGEVI 347
UGT87E3     EWLDSQPNGS--VLYIAQG--SFSSASSAQIDEIAIALCESN-VRFLWVARNET-------------------------- 311
UGT87H3     NWLDSQPAMS--VLYISLG--SFLSVSPNQMNEIVSALNTSQ-IPYFWVALEEV-------------------------- 309
UGT87H2     KWLDSQPSES--VLYISLG--SFLSVSNAQMDEIVEALNNSE-IRYLYMARGET-------------------------- 311
UGT88E4     SWLDSQPSQS--VVFLSFG--SMGRFSKAQLNAIASGLEKSE-QRFLWVVRSELE-----------LDEL-----SLDEL 323
UGT88E5     SWLDSQPSQS--VVFLSFG--SMGRFSKAQLNAIAFGLEKSE-QRFLWVVRSELE-----------LDEL-----SLDEL 322
UGT88E6     ---------------------SMGRFSKAQLNEFAFGLEKSE-QRFLWVVRSELE-----------LDEL-----SLNEL 303
UGT88E11    SWLDSQPSQS--VVLLSFG--SMGRFSQTQLHEIAFGLEKSE-QRFLWIVRSELD-----------LEEL-----SLKEL 322
UGT88E9     SWLDSQPSQS--VVLLSFG--SMGRFSKAQLNEIALGLEKSK-QRFLWIVRSELD-----------LDEL-----SLNEL 318
UGT88E10    RWLDSQPSQS--VVLLSFG--SMGRFSKAQLNEIAIGLEKSK-QRFLWIVRTKLD-----------LEEL-----SLDEL 323
UGT88E7     SWLDLQPRQS--VVLLSFG--SMGRFSKAQLNEIALGLEKSK-QRFLWVVRSELD-----------LDDL-----SLDEL 318
UGT88E8     SWLDLQPCQS--VVLLSFG--SMGRFSKAQLNEIALGLEKSK-QIFLWVVRSELD-----------LDEL-----SLDEL 318
UGT88F9     SWLDKQPSRS--VVFLCFG--SRGSFTVTQLKEIAEGLERSE-HRFLWVVKRPIQDQLGVKQVHDTTNDF-----ELGSV 334
UGT89F1     AWLDAR-DDGS-VVYVCFG--SRTFLTTAQMEILTSALELSG-VQFILSVRGPD---VRHVAQD-------------CSG 314
UGT89D5     RWLDRLDEEGS-VLYVCFG--SQKVMRKEQMEALAFGLERSG-TRFVWVVKEAS--TAEQVEKG-------------YAL 320
UGT89B5     SWLDKR-QDRK-VVYVCFG--SLTILTKDQTDAIARGLLKSG-VHFIWSIKEGVRKNNENDKEA-------------NCG 341
UGT91H7     EWLDTQKGSS--VVYIAFG--AEVKLSQENINELALGIELSG-LPFFWVLRDV------------------KNGSIE--- 329
UGT91H8     DWLDTRESSS--VVYIGFG--SELKLSQQDLTELAYGIELSG-LPFFWALKNL------------------KEGTLE--- 331
UGT91M1     EWLDKQETKS--VVFVGFG--SECKLSKEQVFEIAYGLEVSK-LSFLWALRKP------------------NWSCNDQDF 323
UGT92A6     EWLDAK-PSKS-VLFICFG--SMNTISDKQMMQLGTALERSG-RNFVWVVRPPIGFDINSEFKA-------------EEW 330
UGT92G3     EWLDLK-DEKS-VLYISFG--SQNTISASQMMALAEGLEESE-KTFIWVIRPPFGFDMNGEFK--------------EEW 346
UGT93A5     EWLDKKEPNS--VIYVSFG--TTTTLVEEQIQQIAIGLEQSK-KNFIWVLRDADKGDIFEGN------------EVKRHE 321
UGT95B1     QWLDSKQRGS--VLYVSFG--SEVGPTMEEYTELAQALELCE-QSFIWVVQPGSGRPGPPHMGLRGKPGS-SKPETEEGY 281
UGT71T2     EWLDMQPVAS--VVFLCFG--SMGSLEVKQVEQIAFGLEQAG-VRFLWALREP-PKAQLEDPRD--YVS-------HEDV 316
UGT72AB2    KWLENQPPSS--VIYVCFG--SGGTLSHEQFNELAFGLELSG-CKFLWVVRVPSNDVS-SAYFVGKKE-------DPLEY 274
UGT73C16    KWLDLH-QPKS-VIYVCLG--SLCNLIPSQLIELALALEATK-KPFIWVIREGIHKIEELEK----------------WI 307
UGT73P6     NWLNSK-PDDS-VLYVSFG--SLTRLPHAQLVELAHGLENSR-HNFIWVVRRKD--EDEGS------------------ 304
UGT73P8     KWLNSK-PECS-VLYVSFG--SMNKFPFLQLVEIAHALEDSG-HNFIWVVRKNEENEESDV------------------ 305
UGT74X2     EWLNDKPKGS--VVYVSFG--SLASLNEEQIEEVACGLKDCE-NYFLWVVIPS-----------------------QETK 290
UGT74Z1     NWLNTKPEGS--VIYVSFG--SMVCFSIEQMKEIALGLIGSG-LNFLWVVPNM-----------------------EGKN 266
            .......490.......500.......510.......520.......530.......540.......550.......560
```

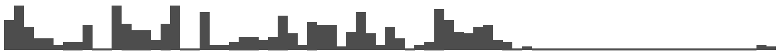

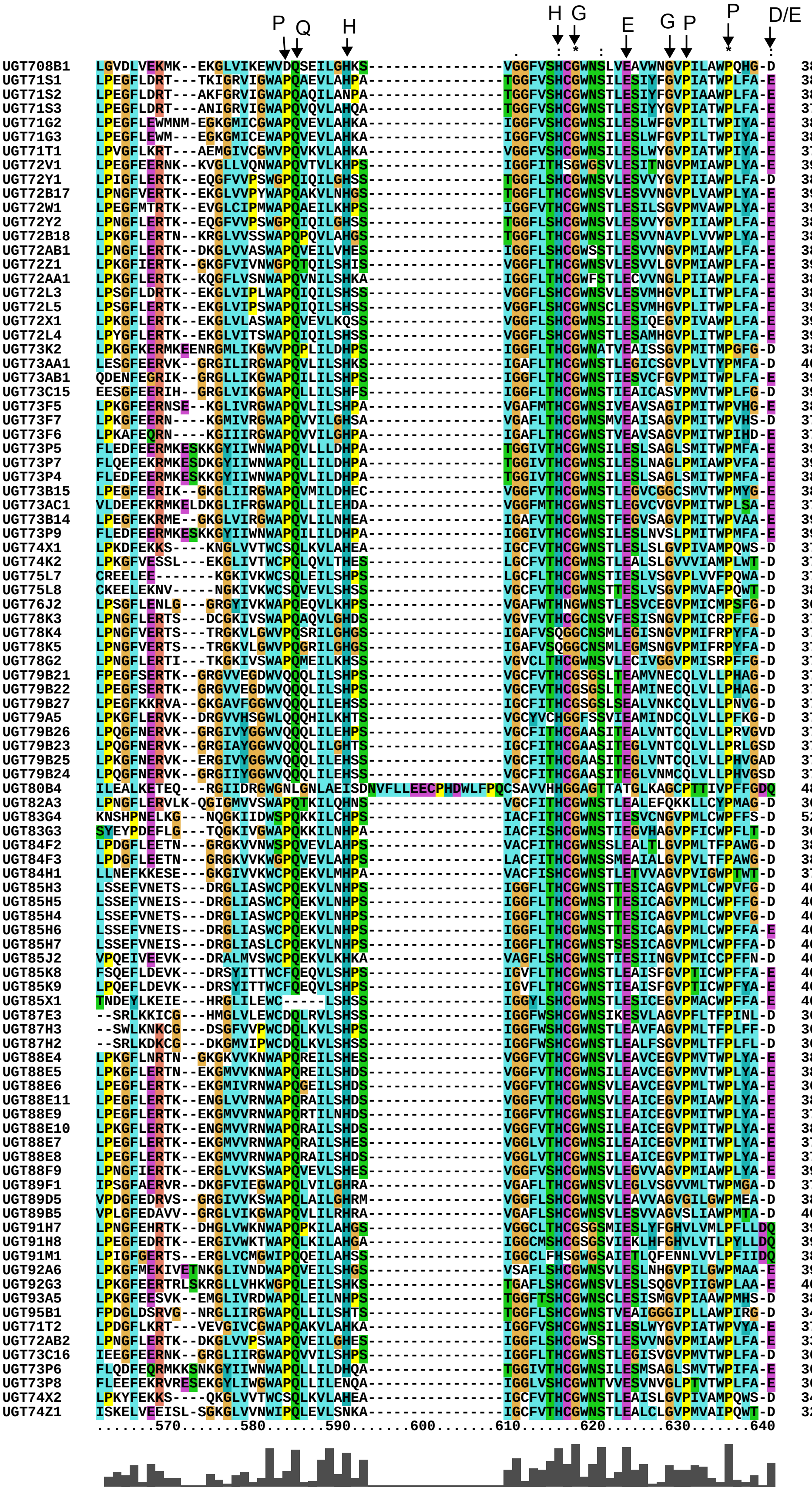
P Q H
H G E G P P D/E
UGT708B1 384
UGT71S1 386
UGT71S2 387
UGT71S3 377
UGT71G2 386
UGT71G3 380
UGT71T1 374
UGT72V1 397
UGT72Y1 386
UGT72B17 391
UGT72W1 391
UGT72Y2 386
UGT72B18 385
UGT72AB1 385
UGT72Z1 392
UGT72AA1 384
UGT72L3 384
UGT72L5 393
UGT72X1 390
UGT72L4 392
UGT73K2 384
UGT73AA1 401
UGT73AB1 394
UGT73C15 396
UGT73F5 381
UGT73F7 372
UGT73F6 373
UGT73P5 392
UGT73P7 393
UGT73P4 387
UGT73B15 386
UGT73AC1 379
UGT73B14 392
UGT73P9 391
UGT74X1 371
UGT74K2 376
UGT75L7 373
UGT75L8 386
UGT76J2 363
UGT78K3 376
UGT78K4 375
UGT78K5 375
UGT78G2 375
UGT79B21 374
UGT79B22 374
UGT79B27 375
UGT79A5 379
UGT79B26 375
UGT79B23 375
UGT79B25 373
UGT79B24 375
UGT80B4 488
UGT82A3 368
UGT83G4 521
UGT83G3 366
UGT84F2 387
UGT84F3 385
UGT84H1 372
UGT85H3 404
UGT85H5 404
UGT85H4 404
UGT85H6 404
UGT85H7 404
UGT85J2 402
UGT85K8 402
UGT85K9 401
UGT85X1 402
UGT87E3 369
UGT87H3 367
UGT87H2 369
UGT88E4 384
UGT88E5 383
UGT88E6 364
UGT88E11 383
UGT88E9 379
UGT88E10 384
UGT88E7 379
UGT88E8 379
UGT88F9 395
UGT89F1 375
UGT89D5 381
UGT89B5 402
UGT91H7 391
UGT91H8 393
UGT91M1 385
UGT92A6 393
UGT92G3 409
UGT93A5 382
UGT95B1 342
UGT71T2 376
UGT72AB2 335
UGT73C16 368
UGT73P6 367
UGT73P8 368
UGT74X2 349
UGT74Z1 328
.......570.......580.......590.......600.......610.......620.......630.......640

```
            Q
            ↓
UGT708B1   QKINAKLVEIGGWGIWNKNWGWSG----------ELVVKGEEIGDAIQEMMEN------KLLKLKAMHLKEEGRKATSVG   448
UGT71S1    QQTNAFELVCELKMGVEIALDYRV----ELDGGANYIVTADKIERGIKSVLE------DGEIRKKVKEMSKKSRKTLLES   456
UGT71S2    QQTNAFVLVSELKIAVEIALDYRV----EFNGEPNYLVMADKIERGIKRVLDK-----DGEERKNLKEISEKSRKTLLED   458
UGT71S3    QQTNAFELVRELKIAVEIALDYRL----EFDIGRNYLLDADKIERGIRGVLDK-----DGEVRKKVKEMSQKSRNVLLEG   448
UGT71G2    QQLNAFRMVKEFGLGVELRMDYR---------NGSDVVLAEEIEKGLKHLMEK-----DNVVQKKLQEM---ARNAVVDG   449
UGT71G3    QQLNAFRMIKEFGLAVELRLDYR---------KGSDVLVAEEIEKGLKTLMDK-----DNVVHKKLQEMKEIARKAVVNG   446
UGT71T1    QQMNAFQMVKELGLAVEIRMDYR--------RGGDLVVQAEEVENGISTLMNG-----SDEIRRKVKDMSEKCRVALMEN   441
UGT72V1    QRMNAALLVEELGVAVRTTVVP-----------GKNVVGREEIASLVRNVFLLEQNGKRNHVRERVREIKISAEKALSQG   466
UGT72Y1    QGVNAAMLSDGVKVALRPEVN------------DNGLVERNEIDKVVRELM---EGEKGVEIRKRMEHLKNAAAVAINEM   451
UGT72B17   QKMNAVLVSEEVKVAMRPKVS------------ENGLVERDEIANVVKRLM---EGEEGKKLRNKMMDLKEAASIALKEN   456
UGT72W1    QKMNAAMLSEKIGVAAR-VVAE-----------EDGIVXXXXXXKLRR--------------------------------   427
UGT72Y2    QGMNAVMLCDGLKVALRAKVN------------ENGLIERDEIAKVVRELL---EGEEGVEICKRMEHLKSVAADAIDEM   451
UGT72B18   QKMNAVILTEDVKVALRPNVG------------ENGLVERQEIASVVKCLM---EGEEGKKLRYQMKDLKDAAVKTLGEN   450
UGT72AB1   QKMNAKELVDVLKVGVRAKVDD-----------ENGIVKRDEVVKAIKGIM---EGDESLEIRKRIKELSVGAANALSEH   451
UGT72Z1    QKLNAVLLCDGLKVAIRPKIN------------ENGLVEREEIVKVIKGVM---LDGEENGIRERIGELRDGAVDALKDN   457
UGT72AA1   QRMIATILADELKVAIRPKVDH-----------ESGIVEKGEISNVLKRLM---VEDEGIKICERMKFLQYAAAGAIEVD   450
UGT72L3    QRMNAVVLSEGLKVGVRPRVN------------ENGIVEREEIVKVIKCLM---EGEEGGTMRDRMKELKNAANNAIKED   449
UGT72L5    QRMNAALLSEGLKVGVRPRVN------------ENGIVERVEIVKVIKCLM---EEEEGRNLCNNMKELKDAAINALKEN   458
UGT72X1    QAMNAVMLSDGLEVAIRLKFE------------DDEIVEKEKIAKVVKCLM---EGEEGKGIRERMKVLKDGAAKALKDD   455
UGT72L4    QRMNAVLLNEGLKVGLRPKVN------------ENGIVEKIEVAEVIKCFM---KDEK---LRNNMKKLKEAATNALKEN   454
UGT73K2    QYYNEKLVTEVHRIGVEVGAAEWSMSPYDAKK---TVVSWERIEKGVKSLMDGDGGGNEIR-KRAKDMKEKAWKAVQEG-   459
UGT73AA1   QFYNEKLVEKVTETGVRLGAEIAVHFGDEDEFGDGFQVNRVNVKKAIEKVMGDGEEKNEMR-ERARKYADMAKKAIEEG-   479
UGT73AB1   QFLNEKLIVEVLKIGVRIGVEVPIRFGDEKKV--GVLVKKSRVVEAIEMCMEGGEE-GEMRRNRVKEHGKMATKALEVDQ   471
UGT73C15   QFLNESLIVEILKVGVKIGVRSPMKWGEEEES--GVLVKKEDVEKGIERLMDETNE-SEERRKRIRELGEMAKKAVEKG-   472
UGT73F5    QFYNEKLITQVRGIGVEVGAGEWSNSGYGERE---KLVSGESIEKALRRLMDGGDDAQEIR-RRAREFGDKAKEAAQEG-   456
UGT73F7    QFYNEKLITQVRGIGVEIGVEEWITTAFRDME---KLVGRDSIEKTMRRLMDDSDEAVEIR-RKAQGFAKLARHAVGEG-   447
UGT73F6    QFYNEKLITQVRGIGVEVGVEEWSIIGFMERK---KLVGRDIIEKAVRRLMDGGIEADEIR-QCAQEYAVKAKRAVQQG-   448
UGT73P5    QFYNEKLLVDVSKIGVPVGSKVNKFWASVGED---AVVKREEIAKAVALLMGSEEESIEMR-RRARKIGDAAKKSIEEG-   467
UGT73P7    QFYNEKLVTEILKIGVPVGVKENKLWASFGEE---AIVRSEEIVKAVEILMGSGQDSKEMR-MRAKMLGDAAKRTIEEG-   468
UGT73P4    QFYNEKLLVDVLKIGVPVGSKVNKFWASVGED---AVVKREEIAKAVVLLMGSEEESIEMR-RRARKIGDAAKKSIEEG-   462
UGT73B15   QFYNAKFLSDVVKIGVGVGVQT---WIGMGGG---EVVKKDVIEKAVKRIMV-GDEAEEMR-NRAKEFGNMAKRAVEVG-   457
UGT73AC1   QFINEKLVTDVLKIGVQVGSKE---WGTWDRER-KELVGREKVEFAVKKLMRKSEETEEMR-RRVKQIAENAKSAVEEG-   453
UGT73B14   QFYNEKLVTEVLKIGVPVGVKK---WVGLFG----DSIQCDLVEKAVKRVME-GEEAEEMR-NKAKLFAEMAKKAGEEG-   462
UGT73P9    QFYNEKLLVDVLKIGVPVGAKVNKFWLNTVEE---EVVKREEIVKAVEILMGNGQEGKKMR-MRARKFGDAAKRTIEKG-   466
UGT74X1    QRTNAKFIVDVWKMGIRVP------------IDEKEIVRGDKLKNCILEIME---GEKGKEIKSNATQWKSLAVGAFGEE   436
UGT74K2    QVTNAKLIADVWKTGVKAF------------ADEKEIVRRETVKNCIKEIIE---TEKGNELKKNAMKWMSIAKDSVDEG   441
UGT75L7    QMTNAKLVEDVWKIGVRVDHK----------MNQDGIVIGEDIRNCLEVVMGS--GGKCEELRENSKKWKELSREAVKEG   441
UGT75L8    QSTNAKLIEDVWKCGVRMDNN----------RDEEGIVKADEIRRCLELVIGG--GEKGEELKRNAEKWKSLGREAVKEG   454
UGT76J2    QKINAKYVSDVWKVGVQLQN----------------KLETGEIEKAIRKLML---GDEANEIRENIMNLKEKANLCLKEG   424
UGT78K3    HGMTGRMVEDVWEIGMRIEGG---------------VFTKNGLVKSLNQILV---REEGNKMREKAQKVKRTVLDAAGPQ   438
UGT78K4    QGINARLAVDVWEVGVIIEGR---------------VFSKNGLLKSLDLILV---QQEGKRFRENALKMKKILEEANGPK   437
UGT78K5    QGINARLAVDVWEVGVIIEGR---------------VFSKNGLLKSLDLILG---QEEGKGIRENALKMKKIIEEAYGPK   437
UGT78G2    QKLNARMLESVWEIGVGVDHG---------------VLTKESTVKALKLIMS---SEKGGVMRQNIVKLKESALKAVEQN   437
UGT79B21   QFINARIMSGDLKVGVEVEKCKE-----------NGLFTREAVCKAVMDVMDN-ESELGHMVRTNHAKWREFLLSKGLEN   442
UGT79B22   QFINARIMSGDLKVGVEVEKCKE-----------NGLFTRKAVCKAVMDVMDN-ESELSHMVRTNHAKWREFLLSKGLEN   442
UGT79B27   QILNARMMGNNLKVGVEVVK-DE-----------NGFFTKESVCEAVKIVMDD-ENEISKEVRGNHAKIREMLLNKDLES   442
UGT79A5    QFFNSKLIAKDLEAGIEVNRNEE-----------DGFFHKEDILKAVKIIMVEDEKEPGKSIRENHMKWRKFILNKEVQD   448
UGT79B26   HIMNARMMSTKLKVGVEVEKGDE-----------DGLFTKESVCNAVNIVMDE-GNEIGREVKANHIQLRKFLLSENLES   443
UGT79B23   HIMNARMMSAKLKVGVEIEKGEE-----------DGLFTKESVCKAVNIVMDE-ENELGREVRANHAKLRNFLLCNNLES   443
UGT79B25   YILNARMMSAKLKVGVEVEKGEE-----------DGLFTKESVCKAVNIVMDE-GNEIGREVRANHAKMRNFLLSENLES   441
UGT79B24   HIMNARMMSAKLKVGVEVEKGEE-----------DGLFTKESVCKAVNTVMDE-ENEVGREVRANHAKLRNILLSNNLKS   443
UGT80B4    FFWGDRIHQKELGPAPIPIYELNVENLSNAIKFMLQPEVKSRAMEVAKLIENEDGVAAAVDAFHRHLPEELPLPTPSHVE   568
UGT82A3    QFVNCVYIVEIWKVGLRLNG-----------------LSQNDVEEGIAMLMED----------NEMDTRLITLYQRIMGI   421
UGT83G4    QLMNKTYICDVWKVGLGFEKD------------ENGLITKEEIKKKVDELLE---DE---EIKERCLKLMEMVVKNKGEG   583
UGT83G3    QFLNKSYICDVWKNGLALEKD------------ENGFISKEEIKRKVEQVLD---DD---DIKEMCLKIKEMTINNMVEG   428
UGT84F2    QVTNAKFLVDVFGVGIRLGYG----------MAANKLVTRDEVKKCLLEATS---GEKAEELKQNAIKWKKAAEEAVAVG   454
UGT84F3    QVTNAKFLVDVFGVGIRLGYS----------RAENKLVSRDEVKKCLLEVMA---GEKAEELKKNAIKWKKAAEAAVAVG   452
UGT84H1    QPTNATLIENVFKNGVKVS------------YGEDGVASAEEIERCIWEVMD---GPNASEIKQRAVEIKESARNALKEG   437
UGT85H3    QPTNCRFICNEWEIGLEIDT----------------NVKREEVEKLVNELIV---GEKGKKMREKAMELKNKAMEDTRVG   465
UGT85H5    QPTNCRFICNEWEIGLEIDT----------------DVKREEVEKLINELMV---GEKGKKMKEKAMELKKKAEEDTRPY   465
UGT85H4    QPTNCRFICNEWEIGLEIDT----------------NVKREEVEKLVNELIV---GEKGKKMREKAMELKNKAMEDTRVG   465
UGT85H6    QTANCRYICKEWEIGLEIDT----------------NVKREEVEKLVNELMV---GEKGKKMRQKSIELKK-VEKDTRLG   464
UGT85H7    QTANCRYICKEWEIGIEIDT----------------NVKREEVEKLVNELMV---GEKGKKMRQKSIELKKEVEKDTRLG   465
UGT85J2    QILNCKYICSEWNFGMKMDS---------------ENVTRDEVEKLVVELMD---GEKGKEMKNKAIEWKKMAEEATNIN   464
UGT85K8    QQTNCRYLCNSWKMGMEIN---------------H-DVKREEITELVMEMMK---GDKGKEMRLKSIEWKKKAKIATEFG   463
UGT85K9    QQTNCRYLCNSWKMGMEIN---------------H-DVKREEITELVMEMMK---GDKGKEMKQKSLEWKKKAKNATELG   462
UGT85X1    QQTNCFYACNRWGIGMEIDSD-------------S-DVSREQVEGLVKELMG---GEKGKEMKKKCVEWKHKVEVATSLG   465
UGT87E3    QPFNSKMIVEDWKVGLRVKED----------FKGDILVKKCEIVRMVCKFMDL-DSDFTRDIRETSRKVKNICLDAIGNG   438
UGT87H3    QVPNSRKILEVWRNGLELKKSV---------LGSEELITQELILEVIRKLMDY-GSVKGKEIRERAQEVKGICDQAVAKG   437
UGT87H2    QFPNSSQIVDEWKNGWKVSK-----------LESEVILAKEDIEELVKRFMDV-ESQEGKKIRDRARDLKFMCHKAIGEG   437
UGT88E4    QNLNKVILVEEMKVALKLNESK------------DGFVSENELGVRVKELMNS---NKGEEIRQKISVMKISAKKAKEEG   449
UGT88E5    QNLNKVILVEEMKVALKLNGSK------------DGFVSENELGVRVKELMNS---NKGEEIRQKISVMKISAKKAKEEG   448
UGT88E6    QKLNKVILVEEMKVALELNDSK------------DGFVSGNKLEYRVKELMNS---DKGKEIRKRIFEMKISAKKTKEEN   429
UGT88E11   QNLNKVFLVDEMKMAIKVNKSQ------------DGRVSGIELGERVKELMES---DSGKEIRERILKMKISAKEARVGS   448
UGT88E9    QKLNRLILVQELKIALKLNESE------------DGFVNGTELAERVMELMES---DKGKEIREKILKMKISAKEAIGGG   444
UGT88E10   QKLNRLILVQEGKIALKLNQST------------NRFVSGTELGERVMELMES---NKGKEIRDNILKMKISAKKARMEG   449
UGT88E7    QNLNRVILVKETKIALKLNESA------------DKFVSASELGERVIELMES---DKGKNIRERILKMKISANEARGGN   444
UGT88E8    QNLNRVILVKETKIALKLNESA------------DKFVSASELGERVIELMES---DKGKNIRERILKMKISANEARGGS   444
UGT88F9    QHVNKNVMVEDMKVAVGVEQREG-----------DRFVSGEEVEKRVRELMES---ERGSEIREKSLKFKDMARDALGEF   461
UGT89F1    QYTNAKLLVDQLGVAVRVAEGE----------------EKVPEANEFAKRIKASL-GKTKERVRAEELKDAALGSIKKS-   437
UGT89D5    QYVNARLLVEDMGVAVRVCEGA----------------DSVPDPDQLGRVISAVMSGDSSQKRRAKLMKEEAVGAVSKD-   444
UGT89B5    QFVDATLVVDELKVGKKVCEGG----------------ESVPDSDELGRVLAESVSGKGEEMCRALKLQRAAADAVREG-   465
UGT91H7    ALYSRVMQ--EKKVGIEIVRNEQ----------DGSFTRNSVAKALRFAMVD---EEGSAYRKNAKEMGKKYSNKDLHN   455
UGT91H8    CLFSRVLV--EKEVAIEVPRSEQ----------DGSFTRDSVAHTLRLAIVD---EEGSAYRDNAKEMGKVFRSKDLHN   457
UGT91M1    PLNARLLV--DKGLAIEVKRYE-----------DGRFSRNEIAKSLRQAMVL---VEGKELRVKTREAAVIVGNLKLHQ   448
UGT92A6    QFFNCKMLEEEIGVCVEVARGKS------------CEVKWEDIVEKIEMVMSERSESGVKIRENACKIRDMIRNAVKYED   461
UGT92G3    QGYNAKMLVEEMGVSVELTRTVE------------SVISKEDVKRVIGIVMDQEGK-GKEMKEKANEIAVHMREATIERG   476
UGT93A5    QPRNSVLIAQVLKVGLVVKEWDKR----------HELVTALDIENVVRRLLET---KEGDEMRQKALNLKNDIHKSMDEG   449
UGT95B1    QHYDAKLVVGHLRVGYMVSDDLS------------KKVTKDDIVEGIQRLMSD------EEMKKNAEVVSAKFRNGFPR-   403
UGT71T2    QQMNAFQMVRELGLAVEIRVDYS--------KGRDLVR-AEEVENGVSTLMNG-----SDEIRKKVKDMSEKCRVALMEN   442
UGT72AB2   QRMNARELVDVLKVGVRAKVDD-----------ENGIVKRDEVVKAIKRIK---EGDESLEIKNRIKELSVSAANALREH   401
UGT73C16   QFLNGKLVTQVLRVGVSLGVEVPMKLGEEDKK--GVYVKKENIKEAICMVVDEGEEESKKRRERAKKLSEMAKKAVEKD-   445
UGT73P6    QFYNEKLLVDVLKIGVPVGSKVNKFWSSVGED---AVVKREKIAKAVALVMGSEVESREMR-RRIRELGDAAKKTTEEG-   442
UGT73P8    HFFNEKLVVDVLRIGVSVGAKEWRNWNEFGS----EVVKREDIGNAIGLMMNGGEEEIGMR-KRAKELSVAAKKAVQVG-   442
UGT74X2    QTTNAKFIVDVWKIGIRAP------------IDEKQIVRKDKLKDCILEIME---GEKGKEIKNNATQWKSLAVRAFDEE   414
UGT74Z1    QPMNAKFVEDVWKVGIRVL------------VYENGIVTREEIESCIREVME---SEHGREMRINAKKWRELAIEAVSLG   393
           .......650.......660.......670.......680.......690.......700.......710.......720
```

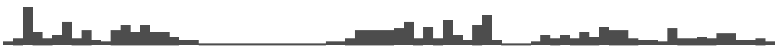

```
UGT708B1   GDCEVTIQKLIEKWKNN-----------------   465
UGT71S1    GSSYTYLGRLIDLIIV------------------   472
UGT71S2    GG-LPHLFRPFD----------------------   469
UGT71S3    GSSYTYLGQLIDYITNQV----------------   466
UGT71G2    GSSFISVGKLIQNMIGSN----------------   467
UGT71G3    GSSFISVGKLIDNMIGSNSL--------------   466
UGT71T1    GSSYTNLLSFIHQLTK------------------   457
UGT72V1    GSSYTALSEVANMIVHKHRFY-------------   487
UGT72Y1    GPSTKALSEVADVWKGI-----------------   468
UGT72B17   GSSTKKICELALKWKGTQTLSNV-----------   479
UGT72W1    ----------------------------------   427
UGT72Y2    GSSTKTLSEVADIWKDI-----------------   468
UGT72B18   GTSTNYISKLALKWSNKSNVTN------------   472
UGT72AB1   GSSMKALSDLVVKLHDY-----------------   468
UGT72Z1    GSSTRAILEFGNCLKRFGRNI-------------   478
UGT72AA1   GSSTTTLSQLVTKWTNLEGCNES-----------   473
UGT72L3    GSSIKTLSQLALKLRNLA----------------   467
UGT72L5    GPSTKTIYQLTLKWRNLV----------------   476
UGT72X1    GSSIQTLSYLANQWENFGGI--------------   475
UGT72L4    GSSIKILSQLTLKWRNLAKETNFKECFTEIVVF-   487
UGT73K2    GSSQNCLTALVDYLQSVVGPRQ----SINN----   485
UGT73AA1   GSSYINMRNLIEDIMHFK----------------   497
UGT73AB1   GSSHFNISCLIQDIMDYQSTNN------------   493
UGT73C15   GSSHSNFTLFIQDIMQNNKDIMPKSFANGNGNSK   506
UGT73F5    GSSYKNLTVLIDDLKRLRDCN-----LQASKC--   483
UGT73F7    GSSHQNLTNLIDEIKLLRDNKGSKFCALTSLR--   479
UGT73F6    GSSHKNLMALIDDLKRLRRYK-----PLDS----   473
UGT73P5    GSSYNNLMQLIDELKSLKISRGLEKTN-------   494
UGT73P7    GHSYNNLIQLIDELKSLKKSKALAVKED------   496
UGT73P4    GSSYNNLMQLIDELKSLKISRGLEKTN-------   489
UGT73B15   GSSYNDFNSLIQDLRSRAH---------------   476
UGT73AC1   GTSYADIDAFIQELKACRFTSQV-----------   476
UGT73B14   GSSYLKLNALIEELGSLSQHQPILQD--------   488
UGT73P9    GYSYNNLIQLLDELTSLKIARELEKSRLDN----   496
UGT74X1    GISQKNIMEFVTSMSNVVNGLTN-----------   459
UGT74K2    GRSDQNIAEFVAVLNQY-----------------   458
UGT75L7    GSSENNLRGFLDLVGLNHDVDVSLCVAENA----   471
UGT75L8    GSSDKNLKSFLHHIGSI-----------------   471
UGT76J2    GSSYSFLDCLVSEILSLKSSTSVCLKSH------   452
UGT78K3    GKAAQDFKTLVELVSSS-----------------   455
UGT78K4    GRATHDFKKLVELVSSS-----------------   454
UGT78K5    GRATKDFKKLMELVSSS-----------------   454
UGT78G2    GTSTKNFTTLIQIVTS------------------   453
UGT79B21   -SYVDNLVQKLDSLLKS-----------------   458
UGT79B22   -SYVDDLVQKLDSLLKS-----------------   458
UGT79B27   -SYIDTFCKKLQEIVQEIINGVFM----------   465
UGT79A5    -KFIKDLVAQLKSLA-------------------   462
UGT79B26   -SCVDNFCQKLRHLL-------------------   457
UGT79B23   -SSVDSFCQELYDLL-------------------   457
UGT79B25   -SCVDSFCRKLYDLL-------------------   455
UGT79B24   -SCVDSFCQELYDLV-------------------   457
UGT80B4    EDNLSPLEWFFDQLAKWCCVPCGGV---------   593
UGT82A3    NNGALLFKSFVEQLKKLNS---------------   440
UGT83G4    ---DNNLNKFINWAKE------------------   596
UGT83G3    GQSSNNLQKFISWVN-------------------   443
UGT84F2    GSSDRNLDAFLKDIKKRGAFNIQKI---------   479
UGT84F3    GSSDWHLNAFIEDIKKRDP---------------   471
UGT84H1    GSSSNNFNKFIGDLLAKN----------------   455
UGT85H3    GCSYMNLDKVINEVLLKHN---------------   484
UGT85H5    GSSYMNLDKLIKEVLVKQKLDLSC----------   489
UGT85H4    GCSYMNLDKVINEVLLKHN---------------   484
UGT85H6    GSSYMNLDKVINEVLLKQNQT-------------   485
UGT85H7    GSSYMNLDKVINEVLLKQNRT-------------   486
UGT85J2    GSSSLNLDKLVSEVLLFKSLG-------------   485
UGT85K8    GSSYNNFYNLIKKVIHHNAI--------------   483
UGT85K9    GSSYNNFNNLIKKVIHHKAI--------------   482
UGT85X1    GSSYNNFQSLVLQLKKIKTEQRNF----------   489
UGT87E3    GSADTDLNAFIADIVHFTNA--------------   458
UGT87H3    GSSNTNLNLFIEDFLCVQGH--------------   457
UGT87H2    GSSYTNLDAFISDISFISRCLE------------   459
UGT88E4    GSSLVDLNKLVQLWNKKK----------------   467
UGT88E5    GSSLVDLNKLVQLWNKKK----------------   466
UGT88E6    GSSIIALNKLTRLWNGNKDNI-------------   450
UGT88E11   GSSLVDMKRLGDSWREHASWDSSSPNSPLAC---   479
UGT88E9    GSSLVDLKKLGDSWKEHDSWNNLSPNSPFLFR--   476
UGT88E10   GSSVVDLKRFGDSKGEYDSWNNYH-QIPLPF---   479
UGT88E7    GSSLVDLKRLKDSWKERDSYNELSPNSPFLIRE-   477
UGT88E8    GSSLVDLKRLGDSWKELDSCNKLSPNSPFLIY--   476
UGT88F9    GSSTKALANLVQTLNGINH---------------   480
UGT89F1    GSSQKQLDALVKELNEVKNHQ-------------   458
UGT89D5    GVSFKELNELIQALKQLGVKEGS-----------   467
UGT89B5    GSSDNNLRCLMEQLVLQ-----------------   482
UGT91H7    -QYIENFISSLYKHRHV-----------------   471
UGT91H8    -HYVDDLIAALHKYRVPSNSIN------------   478
UGT91M1    DHYIAEFVQFLKDGIKKRI---------------   467
UGT92A6    GFKGSSVNGIDEFLAAAALSKKEPQQCQ------   489
UGT92G3    EEKGSSLRAMDDFVRTIL----------------   494
UGT93A5    GVSRKEMDSFIDHITR------------------   465
UGT95B1    -SSVAALDAFKDCINQRSV---------------   421
UGT71T2    GSSYTDLLSLIHELTK------------------   458
UGT72AB2   GSSKKALSDLALRWHI------------------   417
UGT73C16   GSSHLNITLLIQDIMQHQSSNKIET---------   470
UGT73P6    GSSYNNLMQLIDELKSLKLSRGLEEQ--------   468
UGT73P8    GSSYNNMVELIQELKSIKLAKVQA----------   466
UGT74X2    GSSQKNIMEFVTSLFHLQ-AIDK-----------   436
UGT74Z1    GSSDNNINEFVNSLNRS-----------------   410
           .......730.......740.......750....
```

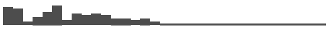

Supplement: Figure S2 — Multiple sequence alignment of 96 chickpea UGTs. The important conserved residues of PF00201 pfam family are marked with an arrow. This and the following sequence alignments are generated using ClustalX [58]. (PDF) [file pone.0109715.s002.pdf]
