# Supplementary material for: Genome-Wide Identification and Tissue-Specific Expression Analysis of UDP-Glycosyltransferases Genes Confirm Their Abundance in Cicer arietinum (Chickpea) Genome
Source: PLoS One. 2014 Oct 7;9(10):e109715. doi: 10.1371/journal.pone.0109715 (PMC4188811; doi:10.1371/journal.pone.0109715)

Figure S3 Multiple sequence alignment of four chickpea UGTs [Ca_06794, Ca_06153 (by MEME-MAST), Ca_27131 and Ca_19130] identified by HMM search

```
                                             *   :              :      .:  . :
Ca_27131        -----------MTNGYLETTIDWIPGIKEIRLKDIP----------SFIRTTDPNDLMLDFLSVECQRSQKASAIILN--      57
Ca_06153        MVLPATNSATGNGSEAQRQITKLEPALILIELVNSRMPTSEFHHPLSRVRQPIGYKLSYQIVDALIWLGIHDLVNEFRKK      80
Ca_06794        ------MEGVEVEQPLKLYFIPYLASGHMIPLCDIATLFASLLQHVTIITTPFNAQILRKSLSSADPSFVRIHTVDFPSQ      74
Ca_19130        --------------------------MCQIQNKDV-------------IANDDSENLFRKAIVITALG-----FILCN--      34
                1.......10........20........30........40........50........60........70........80
```

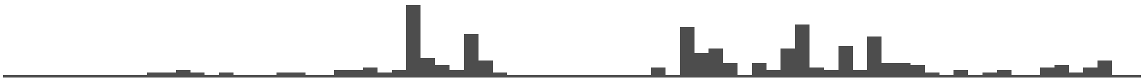

```
                            .       :                                               * .:
Ca_27131        -------TFDAIEHDVLEAFSSTNLPPVYSIGP---------------LNFLLKDVTDNELDTIGSNLWKEETECLEWL-     114
Ca_06153        K----LKLGAMTHHLRGSYTSPPEMPYGYIWSPNLVPKPKDWGPNIDIVGFCFLDLASNYEPPKPLVDWLEEGKNPIYVG     156
Ca_06794        QVGLPYGVESISHTTDLAASTKIYRGAMLLHEP---------------IRSFIEKNPPDCIVADFMYPWVHDIANTLRF-     138
Ca_19130        -------VGPIDECSTLEALSLG-------------------------------------VAVIAMPIWTDQ--------      62
                ........90.......100.......110.......120.......130.......140.......150.......160
```

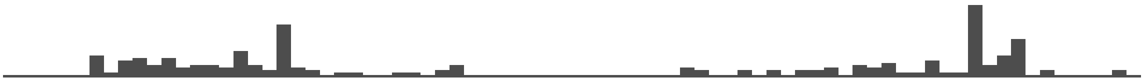

```
Ca_27131        ----ESKEPNSVVYVNFGSITVMTSEQMVEFAWGLANSKIPFLWVIRPDLVAGEKAVLPLEFLEETKDRGLLSS---WCP     187
Ca_06153        FGSLPLQEPEKMTRIIVQALQQTGQRGIINKGWGGLGNLAELKNSKSVYLLNNCPHDWLFPRCTAVVHHGGAGTTAAGLR     236
Ca_06794        ----PYLSFNGFSLFTVSLMESIRTNPSPDSDSDSDSASYEFPNFPHRITMCAKPPKIFTGFMDSLLEKELKSTGLIVNN     214
Ca_19130        ----------------------ITNAKFIVDVWKIG--------------------------------------------      76
                .......170.......180.......190.......200.......210.......220.......230.......240
```

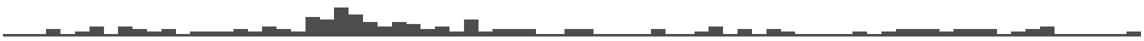

```
                         :   :          *       .   .                                       :.
Ca_27131        QEEVLGHPSIGEQQTNCRFCCNEWGIGLEIEDAKRDKIEG----------------------------------LVRELM     233
Ca_06153        AECPTTIVPFFGDQPFWGERVHARGVGPAPIPVEDFTLERLVDAIRFMLN----------------------PEVKRRAV     294
Ca_06794        FAELDGEECIEHYKKITGHKAWHLGPVSLIRRTDQEKAERGKESVVSVHECLSWLNSKRVNSVIYISFGSLCRFPDKQLY     294
Ca_19130        ------VKAIGDDEK---------GI------VRRETIVD----------------------------------FIRKVM     101
                .......250.......260.......270.......280.......290.......300.......310.......320
```

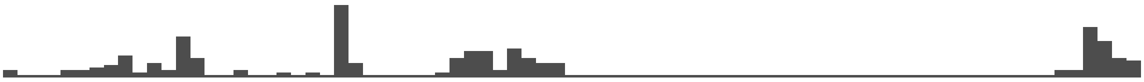

```
                :   . : .   .       .        .  :
Ca_27131        DGEKGKEMKEKALRLKELAKDATSGPYGSSFVNFDNLIRNVLL--------------     276
Ca_06153        ELANAMKNEDGVVGAVNAFYKHYPRQRPDTETEPKQVALVNRHFSIRGCFGCYNSSG     351
Ca_06794        EIACGVEASGYEFVWVVPEKKGXXXXTKAKKRKKSGCRRDSKIETVRRV--------     343
Ca_19130        ESEKGNELEKNCIKWKNLANNSVD-EGGSSDGNIVNL--------------------     137
                .......330.......340.......350.......360.......370.......
```

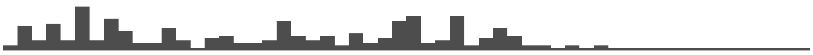

Supplement: Figure S3 — Multiple sequence alignment of four chickpea UGTs [Ca_06794, Ca_06153 (by MEME-MAST), Ca_27131 and Ca_19130] identified by HMM search. (PDF) [file pone.0109715.s003.pdf]
