# Supplementary material for: Genome-Wide Identification and Tissue-Specific Expression Analysis of UDP-Glycosyltransferases Genes Confirm Their Abundance in Cicer arietinum (Chickpea) Genome
Source: PLoS One. 2014 Oct 7;9(10):e109715. doi: 10.1371/journal.pone.0109715 (PMC4188811; doi:10.1371/journal.pone.0109715)
Supplement: Figure S5 — Multiple sequence alignment of chickpea UGTs with experimentally validated UGT proteins. Regions marked in boxes are important for acceptor specificity. (PDF) [file pone.0109715.s005.pdf]

Figure S5 Multiple sequence alignment of chickpea UGTs with experimentally validated UGT proteins. Regions marked in boxes are important for acceptor specificity

Group A1

Regions: Region N1, Region N2

```
UGT78K3            ------MTISSDHIKHVAVYVFPFGSHPVPLLNLVLKLA-HASP-NTLFSFIGTQQSNKPLFSKP------NIPNNIKPF   66
UGT78K4            ------MSNSNENKKHIAVFPFPFSSHLLPLLTLTINLA-HESP-NSHFSFISTQKTNQTLFSKT------NIPKNIKHY   66
UGT78K5            ------MSNSNENKKHIAVFPFPFSSHLLPLLTLTIKLA-HESP-NSHFSFIATEKTNQTLFSKT------NIPNNIKHY   66
UGT78G2            MMPNLLKDETNVEKLHVAILAFPFGTHASPLLSLVRKIA-GEVP-KVTFSFFTTTASNATLFSP-----NNDCLPNIKHY   73
Anthocyanidin_3G2  ---------MTTSQLHIAFLAFPFGTHATPLLTLVQKIS-PFLPSSTIFSFFNTSSSNSSIFS------KVPNQENIKIY   64
Anthocyanidin_3G3  -------MSQTTTNPHVAVLAFPFSTHAAPLLAVVRRLA-AAAP-HAVFSFFSTSQSNASIFHDS----MHTMQCNIKSY   67
Anthocyanidin_3G4  ---------MSPVSHVAVLAFPFGTHAAPLLTLVNRLA-ASAP-DIIFSFFSTSSSITTIFSPTN---LISIGSNIKPY   65
Anthocyanidin_3G5  ---MAPADGESSPPPHVAVVAFPFSSHAAVLLSIARALAAAAAPSGATLSFLSTASSLAQLRKASSASAGHGLPGNLRFV   77
Anthocyanidin_3G6  ---MAPADGESSPPPHVAVVAFPFSSHAAVLLSIARALAAAAAPSGATLSFLSTASSLAQLRKASSASAGHGLPGNLRFV   77
Anthocyanidin_3G7  ---MAPADGESSPPPHVAVVAFPFSSHAAVLLSIARALAAAAAPSGATLSFLSTASSLAQLRKASSASAGHGLPGNLRFV   77
Anthocyanidin_3G8  ---MAP------PPPHIAVVAFPFSSHAAVLFSFARALAAAAP-AGTSLSFLTTADNAAQLRKAG------ALPGNLRFV   64
Flavonoid_3G3      -MSTFKNEMNGNNLLHVAVLAFPFGTHAAPLLSLVKKIA-TEAP-KVTFSFFCTTTTNDTLFSR-----SNEFLPNIKYY   72
Kaempferol_3G      ----------MSNYHVAVLAFPFATHAGLLLGLVQRLA-NALP-NVTFTFFNTSKSNSSLFTTP----HDN---NIKPF   60
                   1.......10........20........30........40........50........60........70........80
```

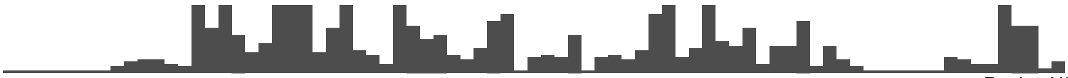

Regions: Region N3, Region N4, Region N5

```
UGT78K3            SIDDGVPKGHVLG-SNPTEKLNLFLQAGHQNLQKGIDLAVAYTK-QRVTCIISDAFVV-PSFFLAQKLNVPWIPIWPPLS  143
UGT78K4            NVSDGIPKGDEL--LPGSEVTFYLQTGPENFQNGIDLAVAESK-KPITCIIADAFVT-PTFDVAKTLNVPWIPVWIPMS  141
UGT78K5            NVSDGIPKGHEL--HPGSEVNFYLQTGPENFQNGIDLAVAESK-KPITCIIADAFVT-PTFDVAKTLNVPWIPVWIPMS  141
UGT78G2            NVDDGLPEGYVPS-GHPLEPIFLFIKAMPENFKSVMDEAVAETG-KDITCLVTDAFYW-FAADLAQQINAKWVPLWTAGP  150
Anthocyanidin_3G2  NVWDGVKEGNDTP-FG-LEAIKLFIQST-LLISKITEEAEEETG-VKFSCIFSDAFLWCFLVKLPKKMNAPGVAYWTGGS  140
Anthocyanidin_3G3  DISDGVPEGYVFA-GRPQEDIELFTRAAPESFRQGMVMAVAETG-RPVSCLVADAFIW-FAADMAAEMGLAWLPFWTAGP  144
Anthocyanidin_3G4  AVWDGSPEGFVFS-GNPREPIEYFLNAAPDNFDKAMKKAVEDTG-VNISCLLTDAFLW-FAADFSEKIGVPWIPVWTAAS  142
Anthocyanidin_3G5  EVPDGAPAAEETVPVP-RQMQLFMEAAEAGGVKAWLEAARAAAGGARVTCVVGDAFVWPAADAAASAG-APWVPVWTAAS  155
Anthocyanidin_3G6  EVPDGAPAAEESVPVP-RQMQLFMEAAEAGGVKAWLEAARAAAGGARVTCVVGDAFVWPAADAAASAG-APWVPVWTAAS  155
Anthocyanidin_3G7  EVPDGAPAAEETVPVP-RQMQLFMEAAEAGGVKAWLEAARAAAGGARVTCVVGDAFVWPAADAAASAG-APWVPVWTAAS  155
Anthocyanidin_3G8  EVPDGVPPGETSCLSPPRRMDLFMAAAEAGGVRVGLEAACASAGGARVSCVVGDAFVW-TADAASAAG-APWVAVWTAAS  142
Flavonoid_3G3      NVHDGLPKGYVSS-GNPREPIFLFIKAMQENFKHVIDEAVAETG-KNITCLVTDAFFW-FGADLAEEMHAKWVPLWTAGP  149
Kaempferol_3G      NISDGVPEGYVVGKGGIEALIGLFFKSAKENIQNAMAAAVEESG-KKITCVMADAFMW-FSGEIAEELSVGWIPLWTSAA  138
                   .........90........100.......110.......120.......130.......140.......150.......160
```

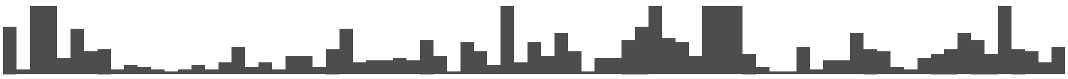

Regions: Region N6

```
UGT78K3            CSLSAHFYTDLIRQNIAENN---EKDRVLDILPGLS-NMRVGDLPQDVTDGGED-EPLFSKTLALLGKVLPQ-AKAVVMN  217
UGT78K4            CSLSVHFHGDIIRAHCSVN----DVNRTLDFLPGLS-VLRVEDLPHDILMTGTEKENVLTRALDSLSKILPE-AKTVVVS  215
UGT78K5            CSLSVHFHGDIIRTHCSVN----DGNRTLDFLPGLS-VLRVEDLPHDTLMKGTDKENDLTRALASLSKILPE-AKAVVVS  215
UGT78G2            HALLTHIYTDLLRH--ACKQVH--DVQNVDLIPGFP-ELKVCDLP-EGVIDDTD--GPFATMLHKMGLELPR-ATAIAIN  221
Anthocyanidin_3G2  CSLAVHLYTDLIRS---------NKETSLKIPGFSSTLSINDIPPEVTAEDLE--GPMSSMLYNMALNLHK-ADAVVLN  207
Anthocyanidin_3G3  NSLSTHVYIDEIREKIGVSGIQGREDELLNFIPGMS-KVRFRDLQEGIVFGNLN--SLFSRMLHRMGQVLPK-ATAVFIN  220
Anthocyanidin_3G4  CSLCLHVYTDEIRSRFAEFDIAEKAEKTIDFIPGLS-AISFSDLPEELIMEDSQ--SIFALTLHNMGLKLHK-ATAVAVN  218
Anthocyanidin_3G5  CALLAHIRTDALREDVGDQAAN-RVDGLLISHPGLA-SYRVRDLPDGVVSGDFN--YVINLLVHRMGQCLPRSAAAVALN  231
Anthocyanidin_3G6  CALLAHIRTDALREDVGDQAAN-RVDEPLISHPGLA-SYRVRDLPDGVVSGDFN--YVINLLVHRMGQCLPRSAAAVALN  231
Anthocyanidin_3G7  CALLAHIRTDSLREDVGDQAAN-RVDEPLISHPGLA-SYRVRDLPDGVVSGDFN--YVISLLVHRMGQCLPRSAAAVALN  231
Anthocyanidin_3G8  CALLAHLRTDALRRDVGDQAAS-RADELLVAHAGLG-GYRVRDLPDGVVSGDFN--YVISLLVHRQAQRLPKAATAVALN  218
Flavonoid_3G3      HSLLTHVYTDLIREKTGSKEVH--DVKSIDVLPGFP-ELKASDLP-EGVIKDID--VPFATMLHKMGLELPR-ANAVAIN  222
Kaempferol_3G      GSLSVHVYTDLIRENVEAQGIAGREDEILTFIPGFA-ELRLGSLPSGVVSGDLE--SPFSVMLHKMGKTIGK-ATALPVN  214
                   .......170.......180.......190.......200.......210.......220.......230.......240
```

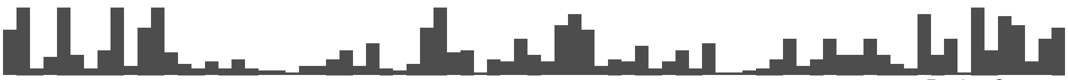

Regions: Region C1

```
UGT78K3            FFEELD-PPLFVEDMR-SKLKCLLYVGFLTFSLPL-----PPLPPSESDETGCLSWLDKQKDRS--VVYVSFG-TVVTPP  287
UGT78K4            FFEELD-LPLFVEDIR-TKLKSMLYV--PLFNLKP-----KTTPQNEIDESGCISFLEVQKAKSLQVVYVSFGTTVVEPP  286
UGT78K5            FFEELD-LPLFVEDIR-TKLQSMLYV--PLFNLKP-----KTTPQNQIDESGCISFLEVQKAKSLQVVYVSFGTTVVEPP  286
UGT78G2            SFATIH--PLIENDLN-SKFKLLLNVG--PFMLTT-----PQP--LISDEQGCLEWLNQHKNFS--VVYISFG-STIIPP  286
Anthocyanidin_3G2  SFQELDRDPLINKDLQ-KNLQKVFNIG--PL-VLQ-----SSR--KL-DESGCIQWLDKQKEKS--VVYLSFG-TVTTLP  272
Anthocyanidin_3G3  SFEELD--DSLTNDLK-SKLKTYLNIG--PFNLIT-----PPP--VVPNTTGCLQWLKERKPTS--VVYISFG-TVTTPP  285
Anthocyanidin_3G4  SFEEID--PIITNHLRSTNQLNILNIG--PLQTLS-----SSI--PP-EDNECLKWLQTQKESS--VVYLSFG-TVINPP  283
Anthocyanidin_3G5  TFPGLD-PPDVTAALA-EILPNCVPFG--PYHLLL-AEDDADT-AAPADPHGCLAWLGRQPARG--VAYVSFG-TVACPR  302
Anthocyanidin_3G6  TFPGLD-PPDVTAALA-EILPNCVPFG--PYHLLL-AEDDADT-AAPADPHGCLAWLGRQPARG--VAYVSFG-TVACPR  302
Anthocyanidin_3G7  TFPGLD-PPDVTAALA-EILPNCVPFG--PYHLLL-AEDDADT-AAPADPHGCLAWLGRQPARG--VAYVSFG-TVACPR  302
Anthocyanidin_3G8  TFPGLD-PPDLIAALA-AELPNCLPLG--PYHLLPGAEPTADTNEAPADPHGCLAWLDRRPARS--VAYVSFG-TNATAR  291
Flavonoid_3G3      SFATIH--PLIENELN-SKFKLLLNVG--PFNLTT-----PQR--KVSDEHGCLEWLDQHENSS--VVYISFG-SVVTPP  287
Kaempferol_3G      SFEELD--PPIVEDLK-SKFNNFLNVG--PFNLTT-----PPPSANITDEYGCIAWLDKQEPGS--VAYIGFG-TVATPP  281
                   .......250.......260.......270.......280.......290.......300.......310.......320
```

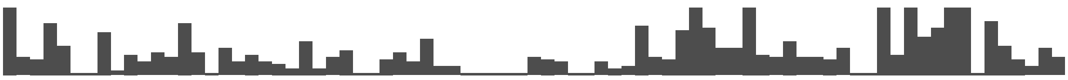

```
                   *:  ::  **    **:*:::        *  .*::::      *  :: *.**  :*  :  ::*.  :.:  *   *   * :
UGT78K3           PNELIAVAEALEESGFPFLWSLKDHLKGLLPNGFLERTS--DCGKIVSWAPQAQVLGHDSVGVFVTHCGCNSVFESISNG   365
UGT78K4           EHEIVALAEALEEYGFPFIWSLKENLRNLLPNGFVERTS--TRGKVLGWVPQSRILGHGSIGAFVSQGGCNSMLEGISNG   364
UGT78K5           QHEIVALAEALEEYGFPFIWSLKENLRNLLPNGFVERTS--TRGKVLGWVPQGRILGHGSIGAFVSQGGCNSMLEGMSNG   364
UGT78G2           PHELIALAESLEECGFPFIWALRGDPKEKLPNGFLERTI--TKGKIVSWAPQMEILKHSSVGVCLTHCGWNSVLECIVGG   364
Anthocyanidin_3G2 PNEIGSIAEALETKKTPFIWSLRNNGVKNLPKGFLERTK--EFGKIVSWAPQLEILAHKSVGVFVTHCGWNSILEGISFG   350
Anthocyanidin_3G3 PAEVVALSEALEASRVPFIWSLRDKARVHLPEGFLEKTR--GYGMVVPWAPQAEVLAHEAVGAFVTHCGWNSLWESVAGG   363
Anthocyanidin_3G4 PNEMAALASTLESRKIPFLWSLRDEARKHLPENFIDRTS--TFGKIVSWAPQLHVLENPAIGVFVTHCGWNSTLESIFCR   361
Anthocyanidin_3G5 PDELRELAAGLEDSGAPFLWSLREDSWPHLPPGFLDRAAGTGSGLVVPWAPQVAVLRHPSVGAFVTHAGWASVLEGLSSG   382
Anthocyanidin_3G6 PDELRELAAGLEASGAPFLWSLREDSWTLLPPGFLDRAAGTGSGLVVPWAPQVAVLRHPSVGAFVTHAGWASVLEGVSSG   382
Anthocyanidin_3G7 PDELRELAAGLEASAAPFLWSLREDSWTLLPPGFLDRAAGTGSGLVVPWAPQVAVLRHPSVGAFVTHAGWASVLEGVSSG   382
Anthocyanidin_3G8 PDELQELAAGLEASGAPFLWSLR-GVVAAAPRGFLERAP----GLVVPWAPQVGVLRHAAVGAFVTHAGWASVMEGVSSG   366
Flavonoid_3G3     PHELTALAESLEECGFPFIWSFRGDPKEKLPKGFLERTK--TKGKIVAWAPQVEILKHSSVGVFLTHSGWNSVLECIVGG   365
Kaempferol_3G     PNELKAMAEALEESKTPFLWSLKDLFKSFFPEGFLERTS--EYGKIVSWAPQVQVLSHGSVGVFINHCGWNSVLESIAAG   359
                  .......330.......340.......350.......360.......370.......380.......390.......400
```

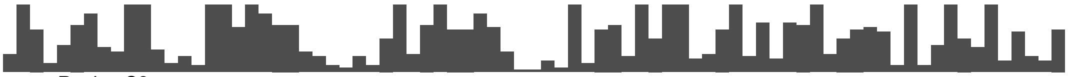

Region C2

```
                  **:   **:*.*: :.        *   .*  ..    ::        .   :  :.  .  ::  .     :      *          .
UGT78K3           VPMICRPFFGDHGMTGRMVEDVWEIGMRIEGGVFTKNGLVKSLNQILVREE-GNKMREKAQKVKRTVLDAAGPQGKAAQD   444
UGT78K4           VPMIFRPYFADQGINARLAVDVWEVGVIIEGRVFSKNGLLKSLDLILVQQE-GKRFRENALKMKKILEEANGPKGRATHD   443
UGT78K5           VPMIFRPYFADQGINARLAVDVWEVGVIIEGRVFSKNGLLKSLDLILGQEE-GKGIRENALKMKKIIEEAYGPKGRATKD   443
UGT78G2           VPMISRPFFGDQKLNARMLESVWEIGVGVDHGVLTKESTVKALKLIMSSEK-GGVMRQNIVKLKESALKAVEQNGTSTKN   443
Anthocyanidin_3G2 VPMICRPFFGDQKLNSRMVESVWEIGLQIEGGIFTKSGIISALDTFFNEEK-GKILRENVEGLKEKALEAVNQMMEVQQK   429
Anthocyanidin_3G3 VPLICRPFFGDQRLNGRMVEDVLEIGVRIEGGVFTKSGLMSCFDQILSQEK-GKKLRENLRALRETADRAVGPKGSSTEN   442
Anthocyanidin_3G4 VPVIGRPFFGDQKVNARMVEDVWKIGVGVKGGVFTEDETTRVLELVLFSDK-GKEMRQNVGRLKEKAKDAVKANGSSTRN   440
Anthocyanidin_3G5 VPMACRPFFGDQRMNARSVAHVWGFGAAFEG-AMTSAGVATAVEELLRGEE-GARMRARAKELQALVAEAFGPGGECRKN   460
Anthocyanidin_3G6 VPMACRPFFGDQRMNARSVAHVWGFGAAFEG-AMTSAGVAAAVEELLRGEE-GARMRARAKVLQALVAEAFGPGGECRKN   460
Anthocyanidin_3G7 VPMACRPFFGDQRMNARSVAHVWGFGAAFEG-AMTSAGVAAAVEELLRGEE-GAGMRARAKELQALVAEAFGPGGECRKN   460
Anthocyanidin_3G8 VPMACRPFFGDQTMNARSVASVWGFGTAFDG-PMTRGAVANAVATLLRGED-GERMRAKAQELQAMVGKAFEPDGGCRKN   444
Flavonoid_3G3     VPMISRPFFGDQGLNTILTESVLEIGVGVDNGVLTKESIKKALELTMSSEK-GGIMRQKIVKLKESAFKAVEQNGTSAMD   444
Kaempferol_3G     VPVICRPFFGDHQLNAWMVEKVWKIGVKIEGGVFTKDGTMLALDLVLSKDKRNTELKQQIGMYKELALNAVGPSGSSAEN   439
                  .......410.......420.......430.......440.......450.......460.......470.......480
```

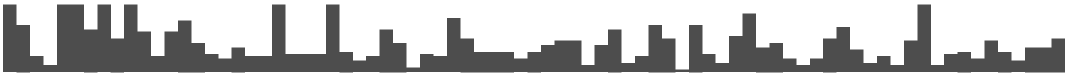

```
                  :  :
UGT78K3           FKTLVELVSSS---   455
UGT78K4           FKKLVELVSSS---   454
UGT78K5           FKKLMELVSSS---   454
UGT78G2           FTTLIQIVTS----   453
Anthocyanidin_3G2 ISRF----------   433
Anthocyanidin_3G3 FITLVDLVSKPKDV   456
Anthocyanidin_3G4 FESLLAAFNKLDS-   453
Anthocyanidin_3G5 FDRFVEIVCRA---   471
Anthocyanidin_3G6 FDRFVEIVCRA---   471
Anthocyanidin_3G7 FDRFVEIVCRA---   471
Anthocyanidin_3G8 FDEFVEIVCRV---   455
Flavonoid_3G3     FTTLIQIVTS----   454
Kaempferol_3G     FKKLVDIITSCN--   451
                  .......490....
```

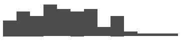

Group A2

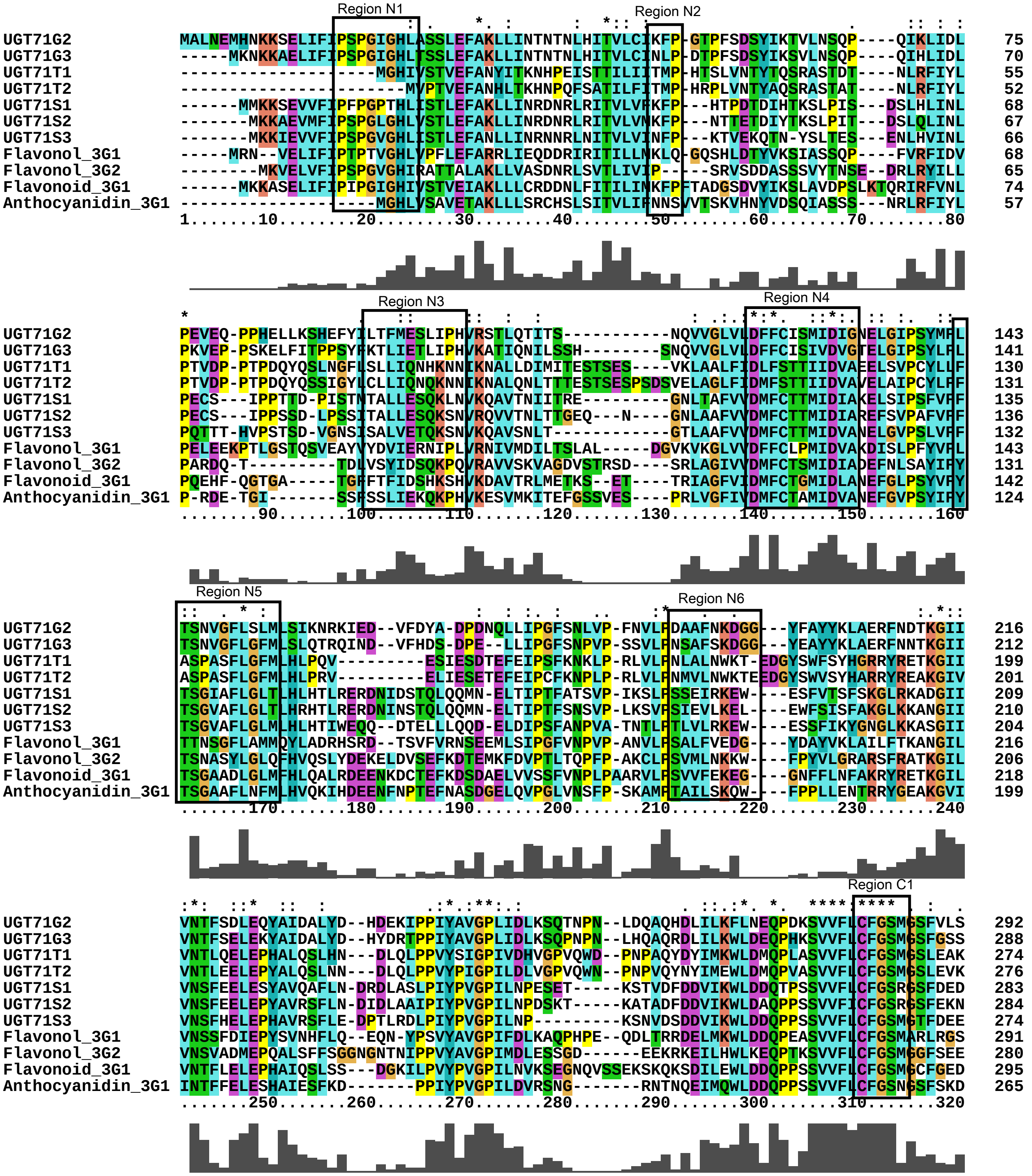
Region N1
Region N2
UGT71G2 MALNEMHNKKSELIFIPSPGIGHLASSLEFAKLLINTNTNLHITVLCIKFP-GTPFSDSYIKTVLNSQP----QIKLIDL 75
UGT71G3 -----MKNKKAELIFIPSPGIGHLTSSLEFAKLLINTNTNLHITVLCINLP-DTPFSDSYIKSVLNSQP----QIHLIDL 70
UGT71T1 --------------------MGHIVSTVEFANYITKNHPEISTTILIITMP-HTSLVNTYTQSRASTDT----NLRFIYL 55
UGT71T2 -----------------------MVPTVEFANHLTKHNPQFSATILFITMP-HRPLVNTYAQSRASTAT----NLRFIYL 52
UGT71S1 ------MMKKSEVVFIPFPGPTHLISTLEFAKLLINRDNRLRITVLVFKFP---HTPDTDIHTKSLPIS---DSLHLINL 68
UGT71S2 -------MKKAEVMFIPSPGLGHLVSTLEFAKLLINRDNRLRITVLVNKFP---NTTETDIYTKSLPIT---DSLQLINL 67
UGT71S3 -------MKKIEVVFIPSPGVGHLISTLEFANLLINRNNRLNITVLVINFP---KTVEKQTN-YSLTES---ENLHVINL 66
Flavonol_3G1 -----MRN--VELIFIPTPTVGHLVPFLEFARRLIEQDDRIRITILLNKLQ-GQSHLDTYVKSIASSQP----FVRFIDV 68
Flavonol_3G2 --------MKVELVFIPSPGVGHIRATTALAKLLVASDNRLSVTLIVIP-----SRVSDDASSSVYTNSE--DRLRYILL 65
Flavonoid_3G1 ------MKKASELIFIPIPGIGHIVSTVEIAKLLLCRDDNLFITILINKFPFTADGSDVYIKSLAVDPSLKTQRIRFVNL 74
Anthocyanidin_3G1 --------------------MGHLVSAVETAKLLLSRCHSLSITVLIFNNSVVTSKVHNYVDSQIASSS---NRLRFIYL 57
Region N3
Region N4
UGT71G2 PEVEQ-PPHELLKSHEFYILTFMESLIPHVRSTLQTITS-----------NQVVGLVLDFFCISMIDIGNELGIPSYMFL 143
UGT71G3 PKVEP-PSKELFITPPSYFKTLIETLIPHVKATIQNILSSH--------SNQVVGLVLDFFCISIVDVGTELGIPSYLFL 141
UGT71T1 PTVDP-PTPDQYQSLNGFLSLLIQNHKNNIKNALLDIMITESTSES----VKLAALFIDLFSTTIIDVAEELSVPCYLLF 130
UGT71T2 PTVDP-PTPDQYQSSIGYLCLLIQNQKNNIKNALQNLTTTESTSESPSDSVELAGLFIDMFSTTIIDVAVELAIPCYLFF 131
UGT71S1 PECS---IPPTTD-PISTNTALLESQKLNVKQAVTNIITRE---------GNLTAFVVDMFCTTMIDIAKELSIPSFVFF 135
UGT71S2 PECS---IPPSSD-LPSSITALLESQKSNVRQVVTNLTTGEQ---N----GNLAAFVVDMFCTTMIDIAREFSVPAFVFF 136
UGT71S3 PQTTT-HVPSTSD-VGNSISALVETQKSNVKQAVSNLT------------GTLAAFVVDMFCTTMIDVANELGVPSLVFF 132
Flavonol_3G1 PELEEKPTLGSTQSVEAYVYDVIERNIPLVRNIVMDILTSLAL-----DGVKVKGLVVDFFCLPMIDVAKDISLPFYVFL 143
Flavonol_3G2 PARDQ-T---------TDLVSYIDSQKPQVRAVVSKVAGDVSTRSD----SRLAGIVVDMFCTSMIDIADEFNLSAYIFY 131
Flavonoid_3G1 PQEHF-QGTGA-----TGFFTFIDSHKSHVKDAVTRLMETKS--ET----TRIAGFVIDMFCTGMIDLANEFGLPSYVFY 142
Anthocyanidin_3G1 P-RDE-TGI-------SSFSSLIEKQKPHVKESVMKITEFGSSVES----PRLVGFIVDMFCTAMIDVANEFGVPSYIFY 124
Region N5
Region N6
UGT71G2 TSNVGFLSLMLSIKNRKIED--VFDYA-DPDNQLLIPGFSNLVP-FNVLPDAAFNKDGG---YFAYYKLAERFNDTKGII 216
UGT71G3 TSNVGFLGFMLSLQTRQIND--VFHDS-DPE--LLIPGFSNPVP-SSVLPNSAFSKDGG---YEAYYKLAERFNNTKGII 212
UGT71T1 ASPASFLGFMLHLPQV---------ESIESDTEFEIPSFKNKLP-RLVLPNLALNWKT-EDGYSWFSYHGRRYRETKGII 199
UGT71T2 ASPASFLGFMLHLPRV---------ELIESETEFEIPCFKNPLP-RLVLPNMVLNWKTEEDGYSWVSYHARRYREAKGIV 201
UGT71S1 TSGIAFLGLTLHLHTLRERDNIDSTQLQQMN-ELTIPTFATSVP-IKSLPSSEIRKEW----ESFVTSFSKGLRKADGII 209
UGT71S2 TSGVAFLGLTLHRHTLRERDNINSTQLQQMN-ELTIPTFSNSVP-LKSVPSIEVLKEL----EWFSISFAKGLKKANGII 210
UGT71S3 TSGVAFLGLMLHLHTIWEQQ--DTELLLQQD-ELDIPSFANPVA-TNTLPTLVLRKEW----ESSFIKYGNGLKKASGII 204
Flavonol_3G1 TTNSGFLAMMQYLADRHSRD--TSVFVRNSEEMLSIPGFVNPVP-ANVLPSALFVEDG----YDAYVKLAILFTKANGIL 216
Flavonol_3G2 TSNASYLGLQFHVQSLYDEKELDVSEFKDTEMKFDVPTLTQPFP-AKCLPSVMLNKKW----FPYVLGRARSFRATKGIL 206
Flavonoid_3G1 TSGAADLGLMFHLQALRDEENKDCTEFKDSDAELVVSSFVNPLPAARVLPSVVFEKEG----GNFFLNFAKRYRETKGIL 218
Anthocyanidin_3G1 TSGAAFLNFMLHVQKIHDEENFNPTEFNASDGELQVPGLVNSFP-SKAMPTAILSKQW----FPPLLENTRRYGEAKGVI 199
Region C1
UGT71G2 VNTFSDLEQYAIDALYD--HDEKIPPIYAVGPLIDLKSQTNPN--LDQAQHDLILKFLNEQPDKSVVFLCFGSMGSFVLS 292
UGT71G3 VNTFSELEKYAIDALYD--HYDRTPPIYAVGPLIDLKSQPNPN--LHQAQRDLILKWLDEQPHKSVVFLCFGSMGSFGSS 288
UGT71T1 VNTLQELEPHALQSLHN---DLQLPPVYSIGPIVDHVGPVQWD--PNPAQYDYIMKWLDMQPLASVVFLCFGSMGSLEAK 274
UGT71T2 VNTLEELEPYALQSLNN---DLQLPPVYPIGPILDLVGPVQWN--PNPVQYNYIMEWLDMQPVASVVFLCFGSMGSLEVK 276
UGT71S1 VNSFEELESYAVQAFLN-DRDLASLPIYPVGPILNPESET-----KSTVDFDDVIKWLDDQTPSSVVFLCFGSRGSFDED 283
UGT71S2 VNSFEELEPYAVRSFLN-DIDLAAIPIYPVGPILNPDSKT-----KATADFDDVIKWLDAQPPSSVVFICFGSRGSFEKN 284
UGT71S3 VNSFHELEPHAVRSFLE-DPTLRDLPIYPVGPILNP---------KSNVDSDDVIKWLDDQPPSSVVFLCFGSMGTFDEE 274
Flavonol_3G1 VNSSFDIEPYSVNHFLQ--EQN-YPSVYAVGPIFDLKAQPHPE--QDLTRRDELMKWLDDQPEASVVFLCFGSMARLRGS 291
Flavonol_3G2 VNSVADMEPQALSFFSGGNGNTNIPPVYAVGPIMDLESSGD------EEKRKEILHWLKEQPTKSVVFLCFGSMGGFSEE 280
Flavonoid_3G1 VNTFLELEPHAIQSLSS---DGKILPVYPVGPILNVKSEGNQVSSEKSKQKSDILEWLDDQPPSSVVFLCFGSMGCFGED 295
Anthocyanidin_3G1 INTFFELESHAIESFKD-------PPIYPVGPILDVRSNG-------RNTNQEIMQWLDDQPPSSVVFLCFGSNGSFSKD 265

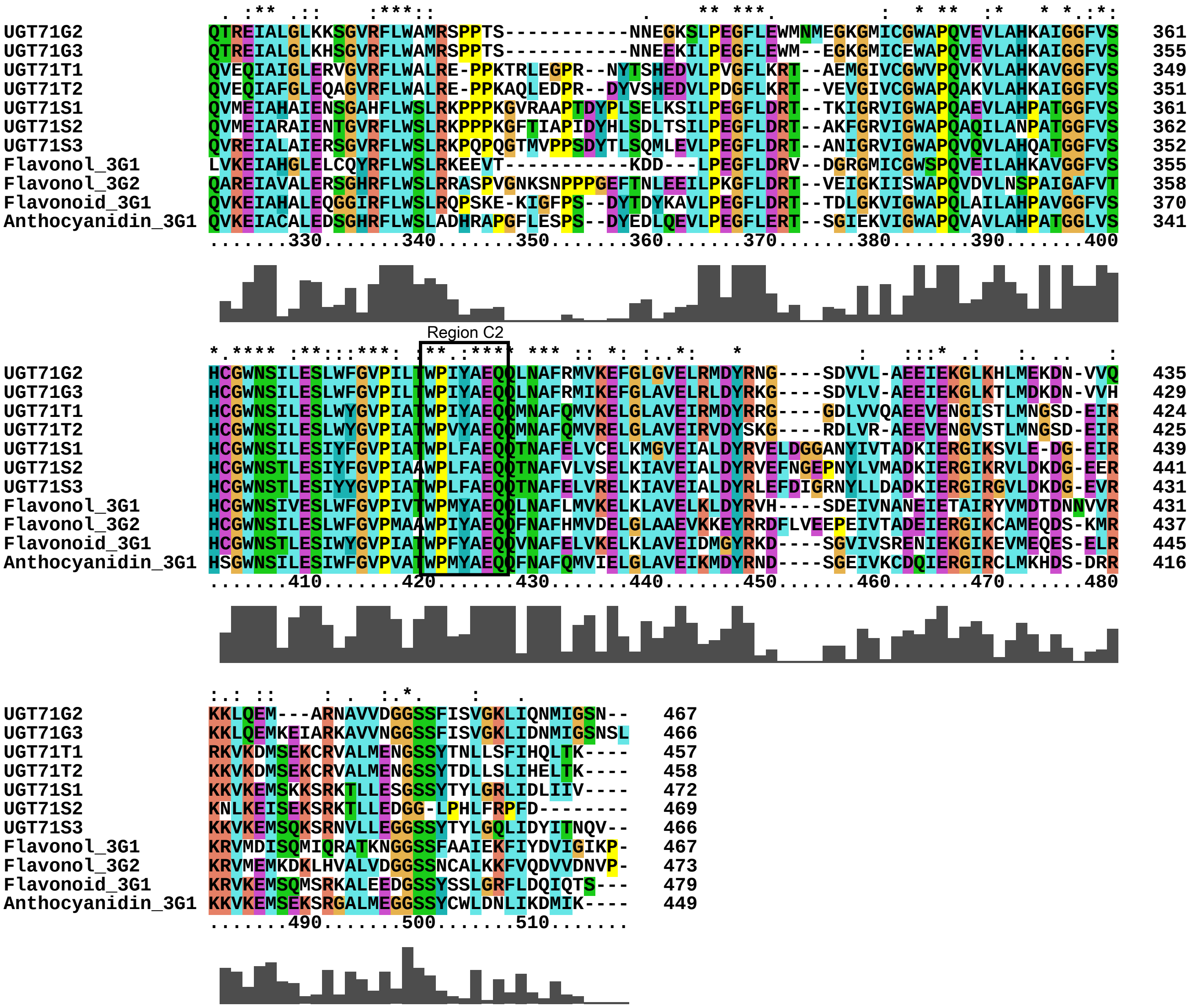

. :** .::   :***::   .   ** ***.   : * ** :*   * *.:*:
UGT71G2 QTREIALGLKKSGVRFLWAMRSPPTS-----------NNEGKSLPEGFLEWMNMEGKGMICGWAPQVEVLAHKAIGGFVS 361
UGT71G3 QTREIALGLKHSGVRFLWAMRSPPTS-----------NNEEKILPEGFLEWM--EGKGMICEWAPQVEVLAHKAIGGFVS 355
UGT71T1 QVEQIAIGLERVGVRFLWALRE-PPKTRLEGPR--NYTSHEDVLPVGFLKRT--AEMGIVCGWVPQVKVLAHKAVGGFVS 349
UGT71T2 QVEQIAFGLEQAGVRFLWALRE-PPKAQLEDPR--DYVSHEDVLPDGFLKRT--VEVGIVCGWAPQAKVLAHKAIGGFVS 351
UGT71S1 QVMEIAHAIENSGAHFLWSLRKPPPKGVRAAPTDYPLSELKSILPEGFLDRT--TKIGRVIGWAPQAEVLAHPATGGFVS 361
UGT71S2 QVMEIARAIENTGVRFLWSLRKPPPKGFTIAPIDYHLSDLTSILPEGFLDRT--AKFGRVIGWAPQAQILANPATGGFVS 362
UGT71S3 QVREIALAIERSGVRFLWSLRKPQPQGTMVPPSDYTLSQMLEVLPEGFLDRT--ANIGRVIGWAPQVQVLAHQATGGFVS 352
Flavonol_3G1 LVKEIAHGLELCQYRFLWSLRKEEVT-----------KDD---LPEGFLDRV--DGRGMICGWSPQVEILAHKAVGGFVS 355
Flavonol_3G2 QAREIAVALERSGHRFLWSLRRASPVGNKSNPPPGEFTNLEEILPKGFLDRT--VEIGKIISWAPQVDVLNSPAIGAFVT 358
Flavonoid_3G1 QVKEIAHALEQGGIRFLWSLRQPSKE-KIGFPS--DYTDYKAVLPEGFLDRT--TDLGKVIGWAPQLAILAHPAVGGFVS 370
Anthocyanidin_3G1 QVKEIACALEDSGHRFLWSLADHRAPGFLESPS--DYEDLQEVLPEGFLERT--SGIEKVIGWAPQVAVLAHPATGGLVS 341
.......330.......340.......350.......360.......370.......380.......390.......400
Region C2
*.**** :**:::***: :** .:**** *** :: *: :..*:   *   :   :::* .:   :. ..   :
UGT71G2 HCGWNSILESLWFGVPILTWPIYAEQQLNAFRMVKEFGLGVELRMDYRNG----SDVVL-AEEIEKGLKHLMEKDN-VVQ 435
UGT71G3 HCGWNSILESLWFGVPILTWPIYAEQQLNAFRMIKEFGLAVELRLDYRKG----SDVLV-AEEIEKGLKTLMDKDN-VVH 429
UGT71T1 HCGWNSILESLWYGVPIATWPIYAEQQMNAFQMVKELGLAVEIRMDYRRG----GDLVVQAEEVENGISTLMNGSD-EIR 424
UGT71T2 HCGWNSILESLWYGVPIATWPVYAEQQMNAFQMVRELGLAVEIRVDYSKG----RDLVR-AEEVENGVSTLMNGSD-EIR 425
UGT71S1 HCGWNSILESIYFGVPIATWPLFAEQQTNAFELVCELKMGVEIALDYRVELDGGANYIVTADKIERGIKSVLE-DG-EIR 439
UGT71S2 HCGWNSTLESIYFGVPIAAWPLFAEQQTNAFVLVSELKIAVEIALDYRVEFNGEPNYLVMADKIERGIKRVLDKDG-EER 441
UGT71S3 HCGWNSTLESIYYGVPIATWPLFAEQQTNAFELVRELKIAVEIALDYRLEFDIGRNYLLDADKIERGIRGVLDKDG-EVR 431
Flavonol_3G1 HCGWNSIVESLWFGVPIVTWPMYAEQQLNAFLMVKELKLAVELKLDYRVH----SDEIVNANEIETAIRYVMDTDNNVVR 431
Flavonol_3G2 HCGWNSILESLWFGVPMAAWPIYAEQQFNAFHMVDELGLAAEVKKEYRRDFLVEEPEIVTADEIERGIKCAMEQDS-KMR 437
Flavonoid_3G1 HCGWNSTLESIWYGVPIATWPFYAEQQVNAFELVKELKLAVEIDMGYRKD----SGVIVSRENIEKGIKEVMEQES-ELR 445
Anthocyanidin_3G1 HSGWNSILESIWFGVPVATWPMYAEQQFNAFQMVIELGLAVEIKMDYRND----SGEIVKCDQIERGIRCLMKHDS-DRR 416
.......410.......420.......430.......440.......450.......460.......470.......480
:.: ::   : . :.*.   :   .
UGT71G2 KKLQEM---ARNAVVDGGSSFISVGKLIQNMIGSN-- 467
UGT71G3 KKLQEMKEIARKAVVNGGSSFISVGKLIDNMIGSNSL 466
UGT71T1 RKVKDMSEKCRVALMENGSSYTNLLSFIHQLTK---- 457
UGT71T2 KKVKDMSEKCRVALMENGSSYTDLLSLIHELTK---- 458
UGT71S1 KKVKEMSKKSRKTLLESGSSYTYLGRLIDLIIV---- 472
UGT71S2 KNLKEISEKSRKTLLEDGG-LPHLFRPFD-------- 469
UGT71S3 KKVKEMSQKSRNVLLEGGSSYTYLGQLIDYITNQV-- 466
Flavonol_3G1 KRVMDISQMIQRATKNGGSSFAAIEKFIYDVIGIKP- 467
Flavonol_3G2 KRVMEMKDKLHVALVDGGSSNCALKKFVQDVVDNVP- 473
Flavonoid_3G1 KRVKEMSQMSRKALEEDGSSYSSLGRFLDQIQTS--- 479
Anthocyanidin_3G1 KKVKEMSEKSRGALMEGGSSYCWLDNLIKDMIK---- 449
.......490.......500.......510.......

Group A4

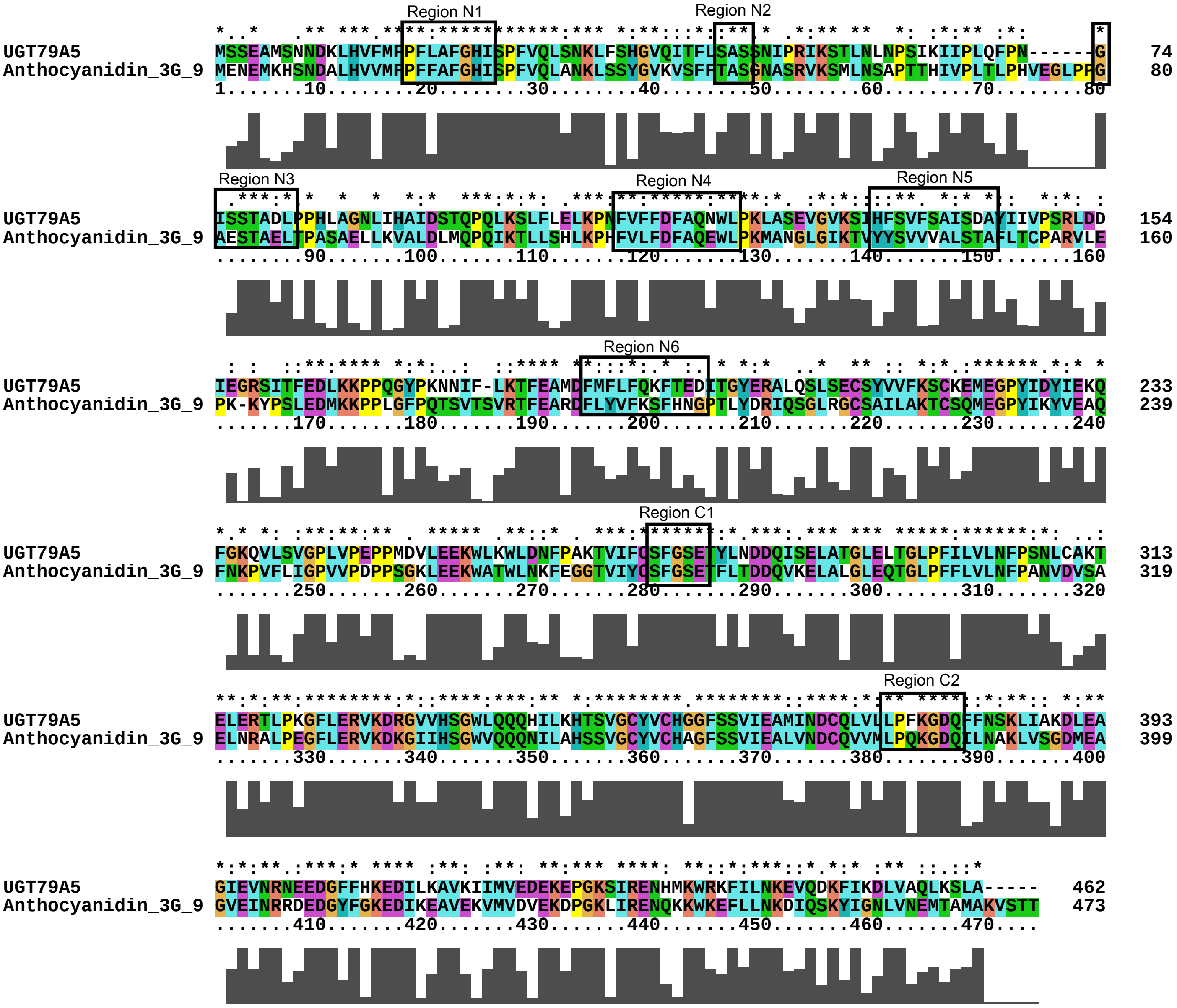
Region N1
Region N2
Region N3
Region N4
Region N5
Region N6
Region C1
Region C2
UGT79A5
Anthocyanidin_3G_9
74
80
154
160
233
239
313
319
393
399
462
473

Group B

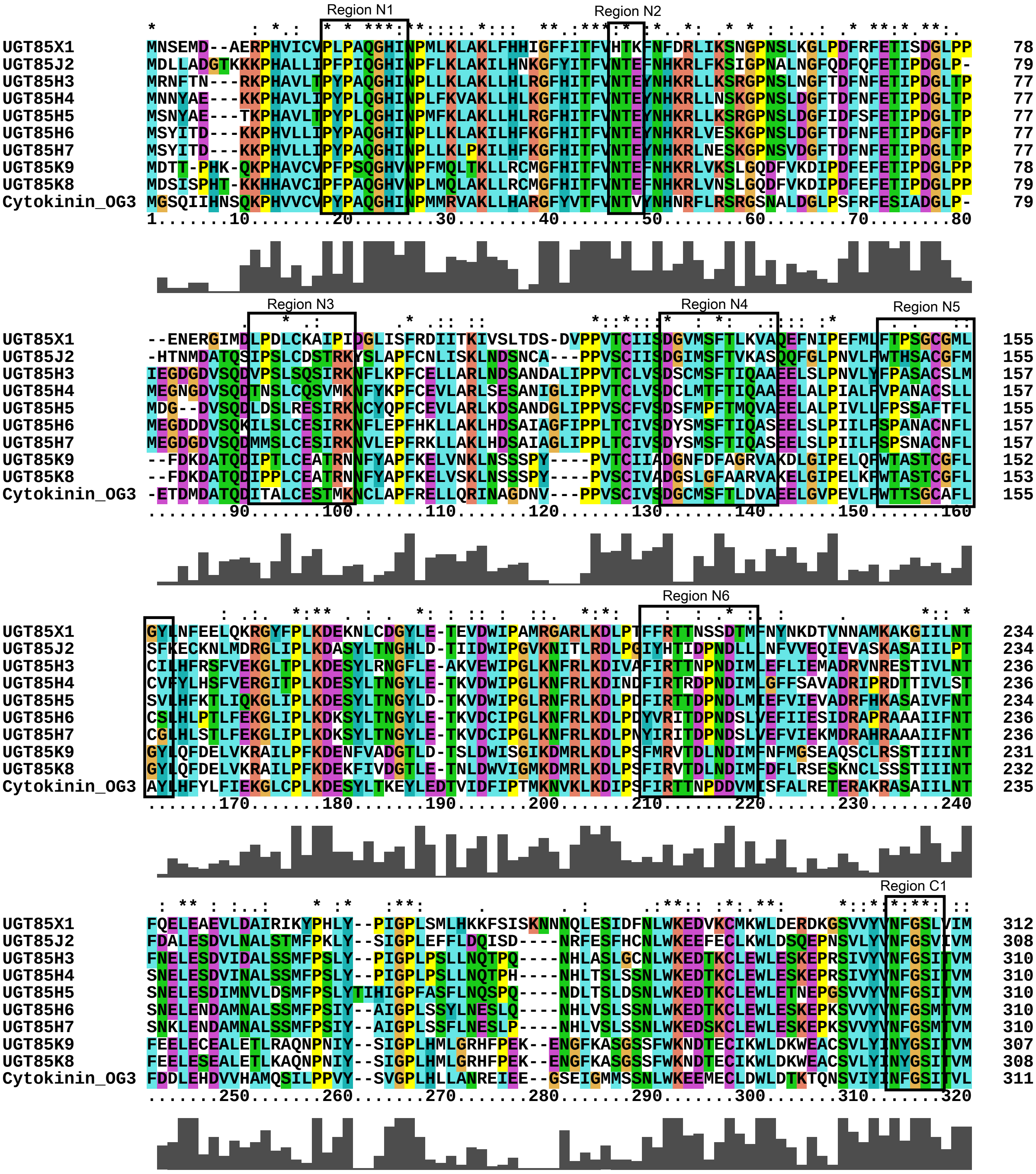
Region N1
Region N2
Region N3
Region N4
Region N5
Region N6
Region C1
UGT85X1
UGT85J2
UGT85H3
UGT85H4
UGT85H5
UGT85H6
UGT85H7
UGT85K9
UGT85K8
Cytokinin_OG3

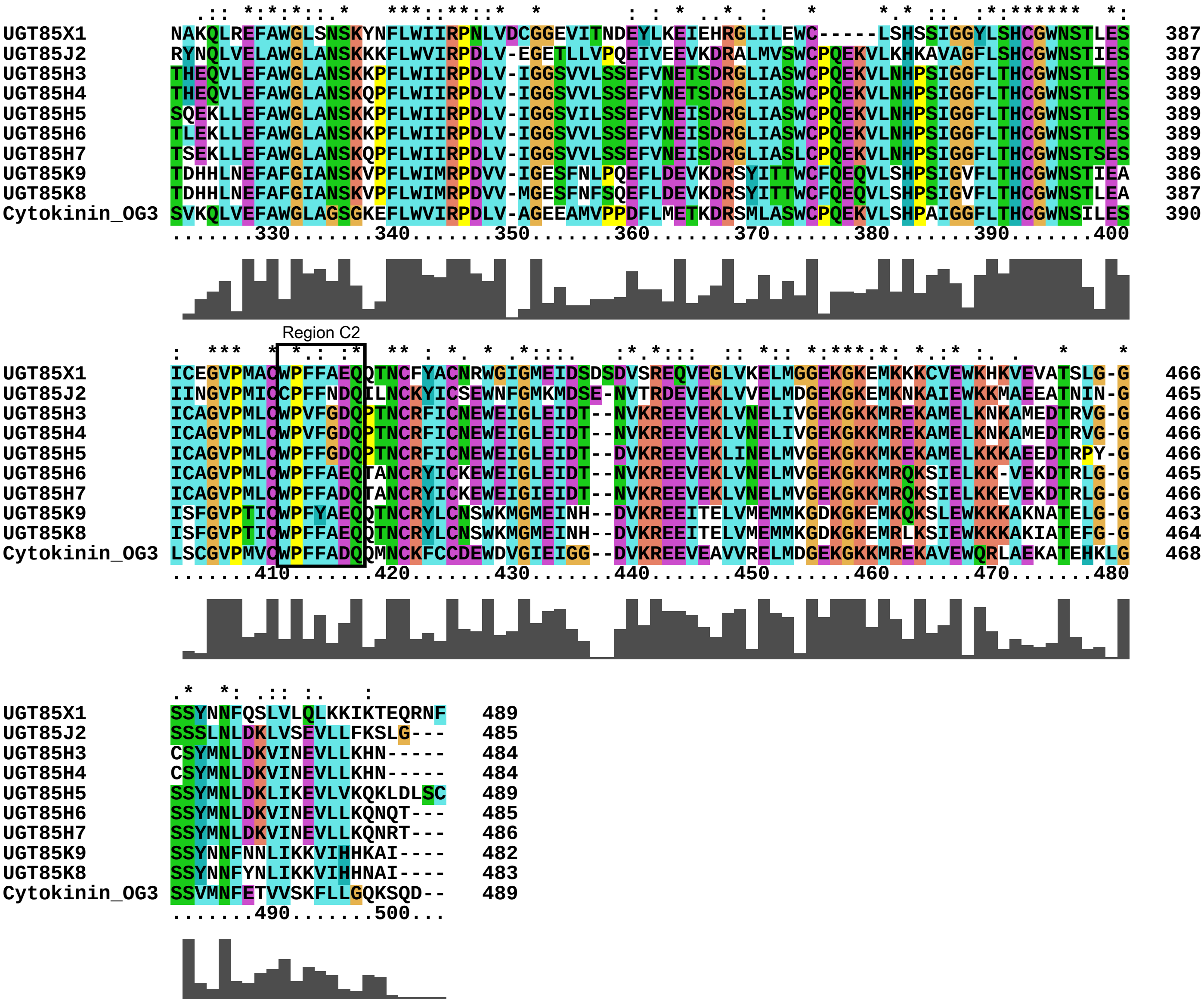
.:: *:*:*::.* ***::**::* * : : * ..*. : * *.* ::. :*:****** *:
UGT85X1 NAKQLREFAWGLSNSKYNFLWIIRPNLVDCGGEVITNDEYLKEIEHRGLILEWC-----LSHSSIGGYLSHCGWNSTLES 387
UGT85J2 RYNQLVELAWGLANSKKKFLWVIRPDLV-EGETLLVPQEIVEEVKDRALMVSWCPQEKVLKHKAVAGFLSHCGWNSTIES 387
UGT85H3 THEQVLEFAWGLANSKKPFLWIIRPDLV-IGGSVVLSSEFVNETSDRGLIASWCPQEKVLNHPSIGGFLTHCGWNSTTES 389
UGT85H4 THEQVLEFAWGLANSKQPFLWIIRPDLV-IGGSVVLSSEFVNETSDRGLIASWCPQEKVLNHPSIGGFLTHCGWNSTTES 389
UGT85H5 SQEKLLEFAWGLANSKKPFLWIIRPDLV-IGGSVILSSEFVNEISDRGLIASWCPQEKVLNHPSIGGFLTHCGWNSTTES 389
UGT85H6 TLEKLLEFAWGLANSKKPFLWIIRPDLV-IGGSVVLSSEFVNEISDRGLIASWCPQEKVLNHPSIGGFLTHCGWNSTTES 389
UGT85H7 TSEKLLEFAWGLANSKQPFLWIIRPDLV-IGGSVVLSSEFVNEISDRGLIASLCPQEKVLNHPSIGGFLTHCGWNSTSES 389
UGT85K9 TDHHLNEFAFGIANSKVPFLWIMRPDVV-IGESFNLPQEFLDEVKDRSYITTWCFQEQVLSHPSIGVFLTHCGWNSTIEA 386
UGT85K8 TDHHLNEFAFGIANSKVPFLWIMRPDVV-MGESFNFSQEFLDEVKDRSYITTWCFQEQVLSHPSIGVFLTHCGWNSTLEA 387
Cytokinin_OG3 SVKQLVEFAWGLAGSGKEFLWVIRPDLV-AGEEAMVPPDFLMETKDRSMLASWCPQEKVLSHPAIGGFLTHCGWNSILES 390
.......330.......340.......350.......360.......370.......380.......390.......400
Region C2
: *** * *.: :* ** : *. * .*:::. :*.*::: :: *:: *:***:*: *.:* :. . * *
UGT85X1 ICEGVPMACWPFFAEQQTNCFYACNRWGIGMEIDSDSDVSREQVEGLVKELMGGEKGKEMKKKCVEWKHKVEVATSLG-G 466
UGT85J2 IINGVPMICCPFFNDQILNCKYICSEWNFGMKMDSE-NVTRDEVEKLVVELMDGEKGKEMKNKAIEWKKMAEEATNIN-G 465
UGT85H3 ICAGVPMLCWPVFGDQPTNCRFICNEWEIGLEIDT--NVKREEVEKLVNELIVGEKGKKMREKAMELKNKAMEDTRVG-G 466
UGT85H4 ICAGVPMLCWPVFGDQPTNCRFICNEWEIGLEIDT--NVKREEVEKLVNELIVGEKGKKMREKAMELKNKAMEDTRVG-G 466
UGT85H5 ICAGVPMLCWPFFGDQPTNCRFICNEWEIGLEIDT--DVKREEVEKLINELMVGEKGKKMKEKAMELKKKAEEDTRPY-G 466
UGT85H6 ICAGVPMLCWPFFAEQTANCRYICKEWEIGLEIDT--NVKREEVEKLVNELMVGEKGKKMRQKSIELKK-VEKDTRLG-G 465
UGT85H7 ICAGVPMLCWPFFADQTANCRYICKEWEIGIEIDT--NVKREEVEKLVNELMVGEKGKKMRQKSIELKKEVEKDTRLG-G 466
UGT85K9 ISFGVPTICWPFYAEQQTNCRYLCNSWKMGMEINH--DVKREEITELVMEMMKGDKGKEMKQKSLEWKKKAKNATELG-G 463
UGT85K8 ISFGVPTICWPFFAEQQTNCRYLCNSWKMGMEINH--DVKREEITELVMEMMKGDKGKEMRLKSIEWKKKAKIATEFG-G 464
Cytokinin_OG3 LSCGVPMVCWPFFADQQMNCKFCCDEWDVGIEIGG--DVKREEVEAVVRELMDGEKGKKMREKAVEWQRLAEKATEHKLG 468
.......410.......420.......430.......440.......450.......460.......470.......480
.* *: .:: :. :
UGT85X1 SSYNNFQSLVLQLKKIKTEQRNF 489
UGT85J2 SSSLNLDKLVSEVLLFKSLG--- 485
UGT85H3 CSYMNLDKVINEVLLKHN----- 484
UGT85H4 CSYMNLDKVINEVLLKHN----- 484
UGT85H5 SSYMNLDKLIKEVLVKQKLDLSC 489
UGT85H6 SSYMNLDKVINEVLLKQNQT--- 485
UGT85H7 SSYMNLDKVINEVLLKQNRT--- 486
UGT85K9 SSYNNFNNLIKKVIHHKAI---- 482
UGT85K8 SSYNNFYNLIKKVIHHNAI---- 483
Cytokinin_OG3 SSVMNFETVVSKFLLGQKSQD-- 489
.......490.......500...

Group C

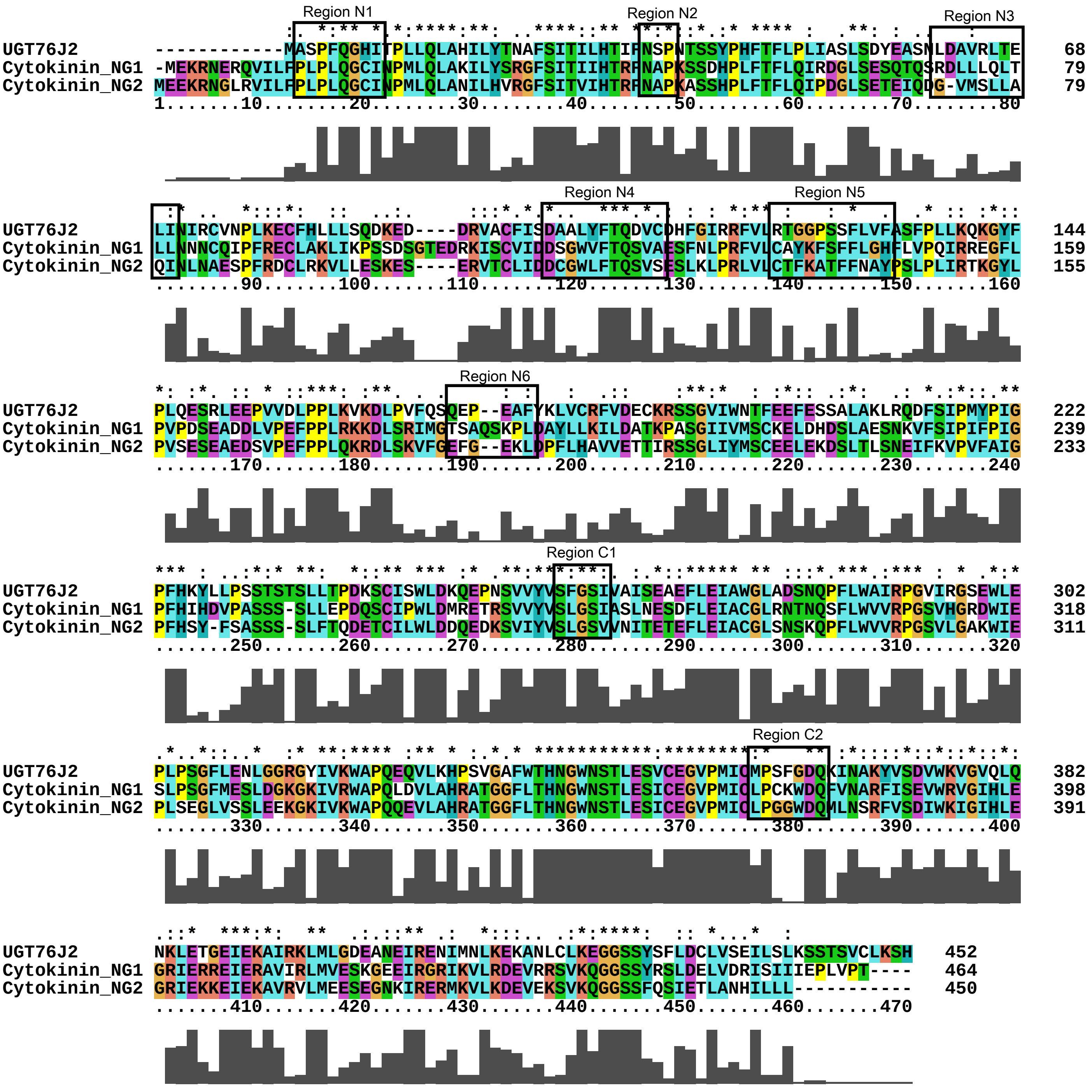

Region N1
Region N2
Region N3
UGT76J2 ------------MASPFQGHITPLLQLAHILYTNAFSITILHTIFNSPNTSSYPHFTFLPLIASLSDYEASNLDAVRLTE 68
Cytokinin_NG1 -MEKRNERQVILFPLPLQGCINPMLQLAKILYSRGFSITIIHTRFNAPKSSDHPLFTFLQIRDGLSESQTQSRDLLLQLT 79
Cytokinin_NG2 MEEKRNGLRVILFPLPLQGCINPMLQLANILHVRGFSITVIHTRFNAPKASSHPLFTFLQIPDGLSETEIQDG-VMSLLA 79
Region N4
Region N5
UGT76J2 LINIRCVNPLKECFHLLLSQDKED----DRVACFISDAALYFTQDVCDHFGIRRFVLRTGGPSSFLVFASFPLLKQKGYF 144
Cytokinin_NG1 LLNNNCQIPFRECLAKLIKPSSDSGTEDRKISCVIDDSGWVFTQSVAESFNLPRFVLCAYKFSFFLGHFLVPQIRREGFL 159
Cytokinin_NG2 QINLNAESPFRDCLRKVLLESKES----ERVTCLIDDCGWLFTQSVSESLKLPRLVLCTFKATFFNAYPSLPLIRTKGYL 155
Region N6
UGT76J2 PLQESRLEEPVVDLPPLKVKDLPVFQSQEP--EAFYKLVCRFVDECKRSSGVIWNTFEEFESSALAKLRQDFSIPMYPIG 222
Cytokinin_NG1 PVPDSEADDLVPEFPPLRKKDLSRIMGTSAQSKPLDAYLLKILDATKPASGIIVMSCKELDHDSLAESNKVFSIPIFPIG 239
Cytokinin_NG2 PVSESEAEDSVPEFPPLQKRDLSKVFGEFG--EKLDPFLHAVVETTIRSSGLIYMSCEELEKDSLTLSNEIFKVPVFAIG 233
Region C1
UGT76J2 PFHKYLLPSSTSTSLLTPDKSCISWLDKQEPNSVVYVSFGSIVAISEAEFLEIAWGLADSNQPFLWAIRPGVIRGSEWLE 302
Cytokinin_NG1 PFHIHDVPASSS-SLLEPDQSCIPWLDMRETRSVVYVSLGSIASLNESDFLEIACGLRNTNQSFLWVVRPGSVHGRDWIE 318
Cytokinin_NG2 PFHSY-FSASSS-SLFTQDETCILWLDDQEDKSVIYVSLGSVVNITETEFLEIACGLSNSKQPFLWVVRPGSVLGAKWIE 311
Region C2
UGT76J2 PLPSGFLENLGGRGYIVKWAPQEQVLKHPSVGAFWTHNGWNSTLESVCEGVPMICMPSFGDQKINAKYVSDVWKVGVQLQ 382
Cytokinin_NG1 SLPSGFMESLDGKGKIVRWAPQLDVLAHRATGGFLTHNGWNSTLESICEGVPMICLPCKWDQFVNARFISEVWRVGIHLE 398
Cytokinin_NG2 PLSEGLVSSLEEKGKIVKWAPQQEVLAHRATGGFLTHNGWNSTLESICEGVPMICLPGGWDQMLNSRFVSDIWKIGIHLE 391
UGT76J2 NKLETGEIEKAIRKLMLGDEANEIRENIMNLKEKANLCLKEGGSSYSFLDCLVSEILSLKSSTSVCLKSH 452
Cytokinin_NG1 GRIERREIERAVIRLMVESKGEEIRGRIKVLRDEVRRSVKQGGSSYRSLDELVDRISIIIEPLVPT---- 464
Cytokinin_NG2 GRIEKKEIEKAVRVLMEESEGNKIRERMKVLKDEVEKSVKQGGSSFQSIETLANHILLL----------- 450

Group D

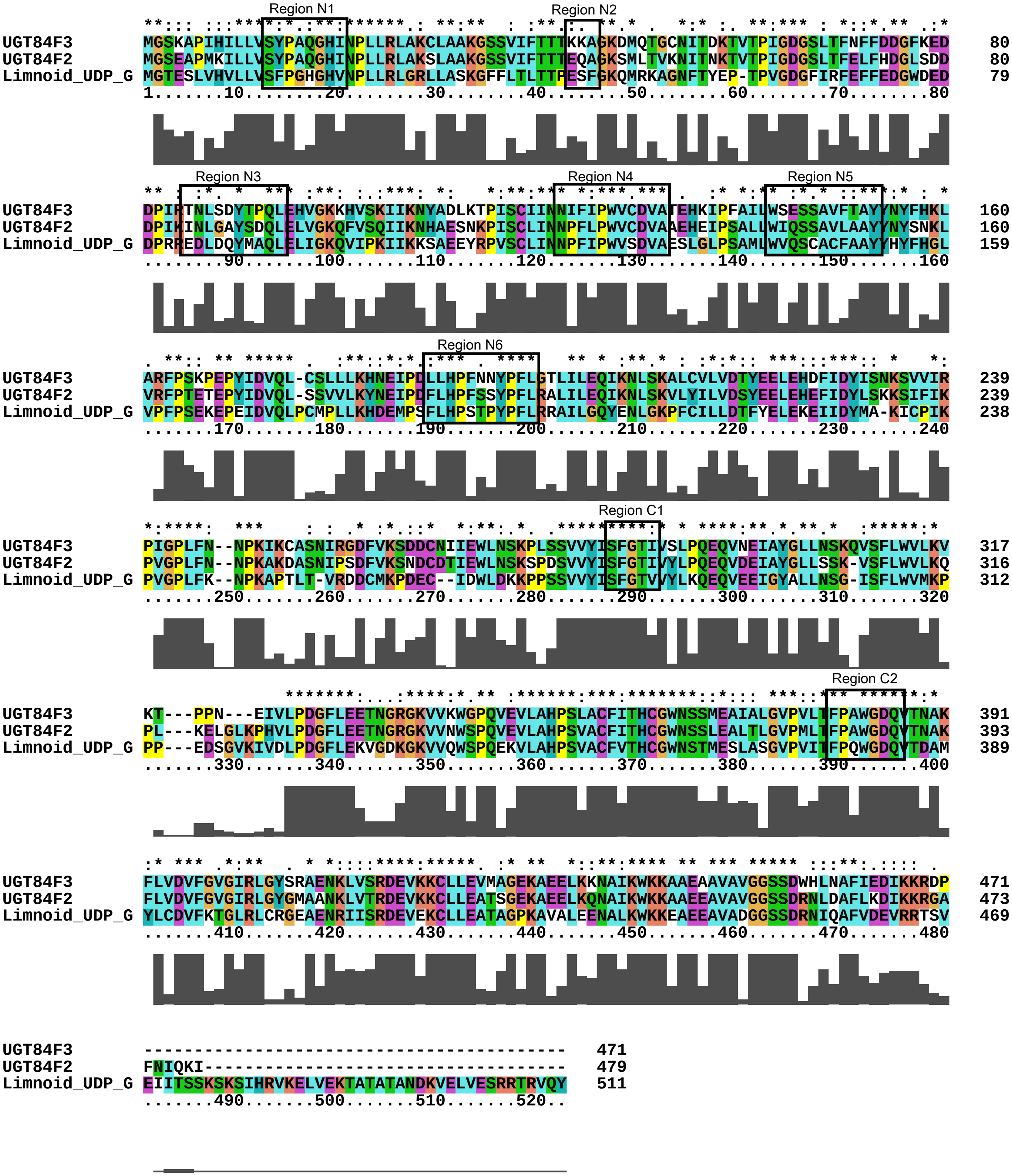
Region N1
Region N2
UGT84F3 MGSKAPIHILLVSYPAQGHINPLLRLAKCLAAKGSSVIFTTTKKAGKDMQTGCNITDKTVTPIGDGSLTFNFFDDGFKED 80
UGT84F2 MGSEAPMKILLVSYPAQGHINPLLRLAKSLAAKGSSVIFTTTEQAGKSMLTVKNITNKTVTPIGDGSLTFELFHDGLSDD 80
Limnoid_UDP_G MGTESLVHVLLVSFPGHGHVNPLLRLGRLLASKGFFLTLTTPESFGKQMRKAGNFTYEP-TPVGDGFIRFEFFEDGWDED 79
Region N3
Region N4
Region N5
UGT84F3 DPIRTNLSDYTPQLEHVGKKHVSKIIKNYADLKTPISCIINNIFIPWVCDVATEHKIPFAILWSESSAVFTAYYNYFHKL 160
UGT84F2 DPIKINLGAYSDQLELVGKQFVSQIIKNHAESNKPISCLINNPFLPWVCDVAAEHEIPSALLWIQSSAVLAAYYNYSNKL 160
Limnoid_UDP_G DPRREDLDQYMAQLELIGKQVIPKIIKKSAEEYRPVSCLINNPFIPWVSDVAESLGLPSAMLWVQSCACFAAYYHYFHGL 159
Region N6
UGT84F3 ARFPSKPEPYIDVQL-CSLLLKHNEIPDLLHPFNNYPFLGTLILEQIKNLSKALCVLVDTYEELEHDFIDYISNKSVVIR 239
UGT84F2 VRFPTETEPYIDVQL-SSVVLKYNEIPDFLHPFSSYPFLRALILEQIKNLSKVLYILVDSYEELEHEFIDYLSKKSIFIK 239
Limnoid_UDP_G VPFPSEKEPEIDVQLPCMPLLKHDEMPSFLHPSTPYPFLRRAILGQYENLGKPFCILLDTFYELEKEIIDYMA-KICPIK 238
Region C1
UGT84F3 PIGPLFN--NPKIKCASNIRGDFVKSDDCNIIEWLNSKPLSSVVYISFGTIVSLPQEQVNEIAYGLLNSKQVSFLWVLKV 317
UGT84F2 PVGPLFN--NPKAKDASNIPSDFVKSNDCDTIEWLNSKSPDSVVYISFGTIVYLPQEQVDEIAYGLLSSK-VSFLWVLKQ 316
Limnoid_UDP_G PVGPLFK--NPKAPTLT-VRDDCMKPDEC--IDWLDKKPPSSVVYISFGTVVYLKQEQVEEIGYALLNSG-ISFLWVMKP 312
Region C2
UGT84F3 KT---PPN---EIVLPDGFLEETNGRGKVVKWGPQVEVLAHPSLACFITHCGWNSSMEAIALGVPVLTFPAWGDQVTNAK 391
UGT84F2 PL---KELGLKPHVLPDGFLEETNGRGKVVNWSPQVEVLAHPSVACFITHCGWNSSLEALTLGVPMLTFPAWGDQVTNAK 393
Limnoid_UDP_G PP---EDSGVKIVDLPDGFLEKVGDKGKVVQWSPQEKVLAHPSVACFVTHCGWNSTMESLASGVPVITFPQWGDQVTDAM 389
UGT84F3 FLVDVFGVGIRLGYSRAENKLVSRDEVKKCLLEVMAGEKAEELKKNAIKWKKAAEAAVAVGGSSDWHLNAFIEDIKKRDP 471
UGT84F2 FLVDVFGVGIRLGYGMAANKLVTRDEVKKCLLEATSGEKAEELKQNAIKWKKAAEEAVAVGGSSDRNLDAFLKDIKKRGA 473
Limnoid_UDP_G YLCDVFKTGLRLCRGEAENRIISRDEVEKCLLEATAGPKAVALEENALKWKKEAEEAVADGGSSDRNIQAFVDEVRRTSV 469
UGT84F3 -- 471
UGT84F2 FNIQKI------------------------------------ 479
Limnoid_UDP_G EIITSSKSKSIHRVKELVEKTATATANDKVELVESRRTRVQY 511

Group E

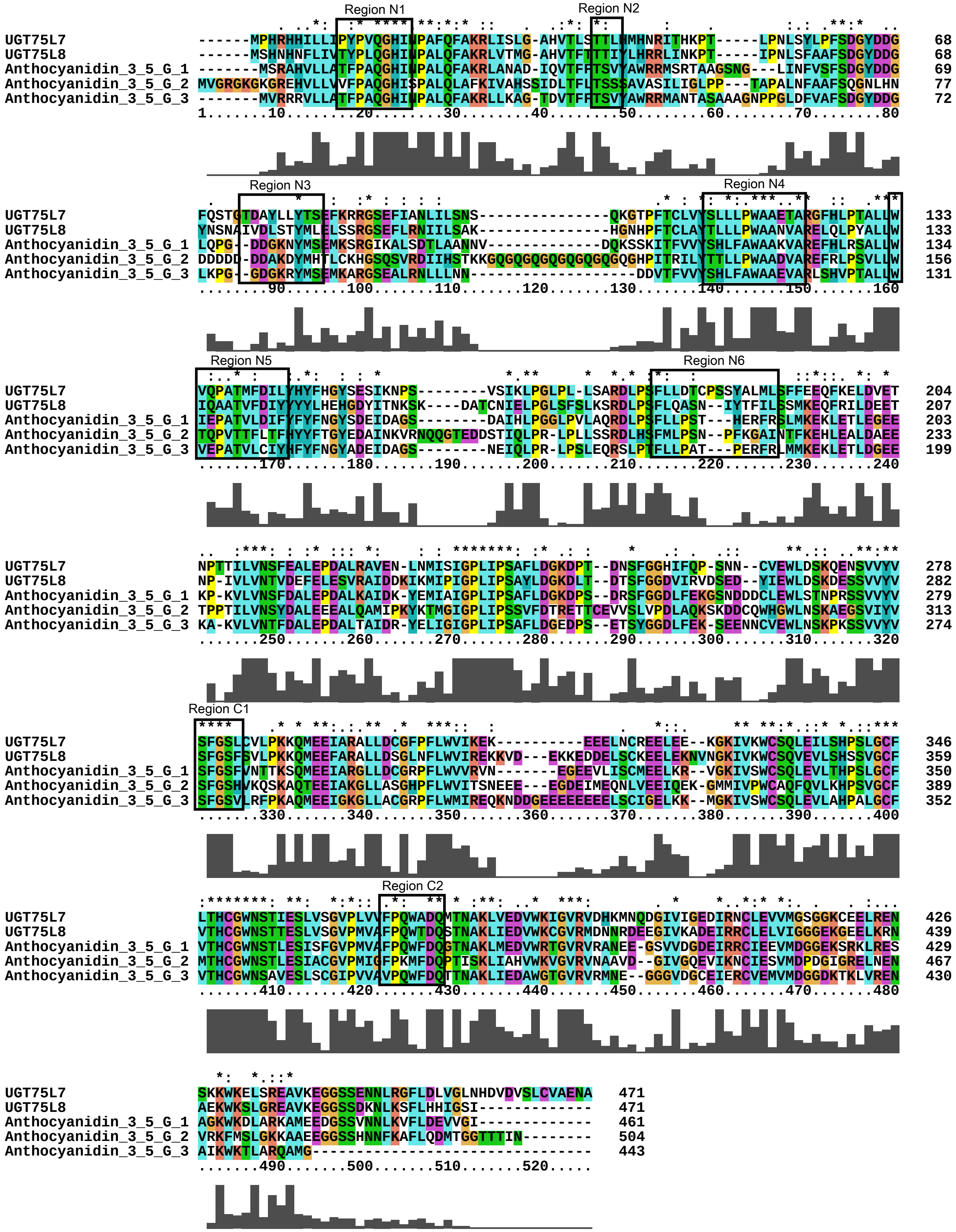

Region N1
Region N2
UGT75L7 ------MPHRHHILLIPYPVQGHINPAFQFAKRLISLG-AHVTLSTTLHMHNRITHKPT-----LPNLSYLPFSDGYDDG 68
UGT75L8 ------MSHNHNFLIVTYPLQGHINPALQFAKRLVTMG-AHVTFTTTIYLHRRLINKPT-----IPNLSFAAFSDGYDDG 68
Anthocyanidin_3_5_G_1 -------MSRAHVLLATFPAQGHINPALQFAKRLANAD-IQVTFFTSVYAWRRMSRTAAGSNG---LINFVSFSDGYDDG 69
Anthocyanidin_3_5_G_2 MVGRGKGKGREHVLLVVFPAQGHISPALQLAFKIVAHSSIDLTFLTSSSAVASILIGLPP---TAPALNFAAFSQGNLHN 77
Anthocyanidin_3_5_G_3 -------MVRRRVLLATFPAQGHINPALQFAKRLLKAG-TDVTFFTSVYAWRRMANTASAAAGNPPGLDFVAFSDGYDDG 72
Region N3
Region N4
UGT75L7 FQSTGTDAYLLYTSEFKRRGSEFIANLILSNS--------------QKGTPFTCLVYSLLLPWAAETARGFHLPTALLW 133
UGT75L8 YNSNAIVDLSTYMLELSSRGSEFLRNIILSAK--------------HGNHPFTCLAYTLLLPWAANVARELQLPYALLW 133
Anthocyanidin_3_5_G_1 LQPG--DDGKNYMSEMKSRGIKALSDTLAANNV------------DQKSSKITFVVYSHLFAWAAKVAREFHLRSALLW 134
Anthocyanidin_3_5_G_2 DDDDD-DDAKDYMHTLCKHGSQSVRDIIHSTKKGQGQGQGQGQGQGQGQGHPITRILYTTLLPWAADVAREFRLPSVLLW 156
Anthocyanidin_3_5_G_3 LKPG--GDGKRYMSEMKARGSEALRNLLLNN-----------------DDVTFVVYSHLFAWAAEVARLSHVPTALLW 131
Region N5
Region N6
UGT75L7 VQPATMFDILYHYFHGYSESIKNPS--------VSIKLPGLPL-LSARDLPSFLLDTCPSSYALMLSFFEEQFKELDVET 204
UGT75L8 IQAATVFDIYYYYLHEHGDYITNKSK----DATCNIELPGLSFSLKSRDLPSFLQASN--IYTFILSSMKEQFRILDEET 207
Anthocyanidin_3_5_G_1 IEPATVLDIFYFYFNGYSDEIDAGS--------DAIHLPGGLPVLAQRDLPSFLLPST---HERFRSLMKEKLETLEGEE 203
Anthocyanidin_3_5_G_2 TQPVTTFLTFHYYFTGYEDAINKVRNQQGTEDDSTIQLPR-LPLLSSRDLHSFMLPSN--PFKGAINTFKEHLEALDAEE 233
Anthocyanidin_3_5_G_3 VEPATVLCIYHFYFNGYADEIDAGS--------NEIQLPR-LPSLEQRSLPTFLLPAT---PERFRLMMKEKLETLDGEE 199
UGT75L7 NPTTILVNSFEALEPDALRAVEN-LNMISIGPLIPSAFLDGKDPT--DNSFGGHIFQP-SNN--CVEWLDSKQENSVVYV 278
UGT75L8 NP-IVLVNTVDEFELESVRAIDDKIKMIPIGPLIPSAYLDGKDLT--DTSFGGDVIRVDSED--YIEWLDSKDESSVVYV 282
Anthocyanidin_3_5_G_1 KP-KVLVNSFDALEPDALKAIDK-YEMIAIGPLIPSAFLDGKDPS--DRSFGGDLFEKGSNDDDCLEWLSTNPRSSVVYV 279
Anthocyanidin_3_5_G_2 TPPTILVNSYDALEEEALQAMIPKYKTMGIGPLIPSSVFDTRETTCEVVSLVPDLAQKSKDDCQWHGWLNSKAEGSVIYV 313
Anthocyanidin_3_5_G_3 KA-KVLVNTFDALEPDALTAIDR-YELIGIGPLIPSAFLDGEDPS--ETSYGGDLFEK-SEENNCVEWLNSKPKSSVVYV 274
Region C1
UGT75L7 SFGSLCVLPKKQMEEIARALLDCGFPFLWVIKEK----------EEELNCREELEE--KGKIVKWCSQLEILSHPSLGCF 346
UGT75L8 SFGSFSVLPKKQMEEFARALLDSGLNFLWVIREKKVD---EKKEDDELSCKEELEKNVNGKIVKWCSQVEVLSHSSVGCF 359
Anthocyanidin_3_5_G_1 SFGSFVNTTKSQMEEIARGLLDCGRPFLWVVRVN-------EGEEVLISCMEELKR--VGKIVSWCSQLEVLTHPSLGCF 350
Anthocyanidin_3_5_G_2 SFGSHVKQSKAQTEEIAKGLLASGHPFLWVITSNEEE---EGDEIMEQNLVEEIQEK-GMMIVPWCAQFQVLKHPSVGCF 389
Anthocyanidin_3_5_G_3 SFGSVLRFPKAQMEEIGKGLLACGRPFLWMIREQKNDDGEEEEEEEELSCIGELKK--MGKIVSWCSQLEVLAHPALGCF 352
Region C2
UGT75L7 LTHCGWNSTIESLVSGVPLVVFPQWADQMTNAKLVEDVWKIGVRVDHKMNQDGIVIGEDIRNCLEVVMGSGGKCEELREN 426
UGT75L8 VTHCGWNSTTESLVSGVPMVAFPQWTDQSTNAKLIEDVWKCGVRMDNNRDEEGIVKADEIRRCLELVIGGGEKGEELKRN 439
Anthocyanidin_3_5_G_1 VTHCGWNSTLESISFGVPMVAFPQWFDQGTNAKLMEDVWRTGVRVRANEE-GSVVDGDEIRRCIEEVMDGGEKSRKLRES 429
Anthocyanidin_3_5_G_2 MTHCGWNSTLESIACGVPMIGFPKMFDQPTISKLIAHVWKVGVRVNAAVD--GIVGQEVIKNCIESVMDPDGIGRELNEN 467
Anthocyanidin_3_5_G_3 VTHCGWNSAVESLSCGIPVVAVPQWFDQTTNAKLIEDAWGTGVRVRMNE--GGGVDGCEIERCVEMVMDGGDKTKLVREN 430
UGT75L7 SKKWKELSREAVKEGGSSENNLRGFLDLVGLNHDVDVSLCVAENA 471
UGT75L8 AEKWKSLGREAVKEGGSSDKNLKSFLHHIGSI------------- 471
Anthocyanidin_3_5_G_1 AGKWKDLARKAMEEDGSSVNNLKVFLDEVVGI------------- 461
Anthocyanidin_3_5_G_2 VRKFMSLGKKAAEEGGSSHNNFKAFLQDMTGGTTTIN-------- 504
Anthocyanidin_3_5_G_3 AIKWKTLARQAMG-------------------------------- 443

Group F

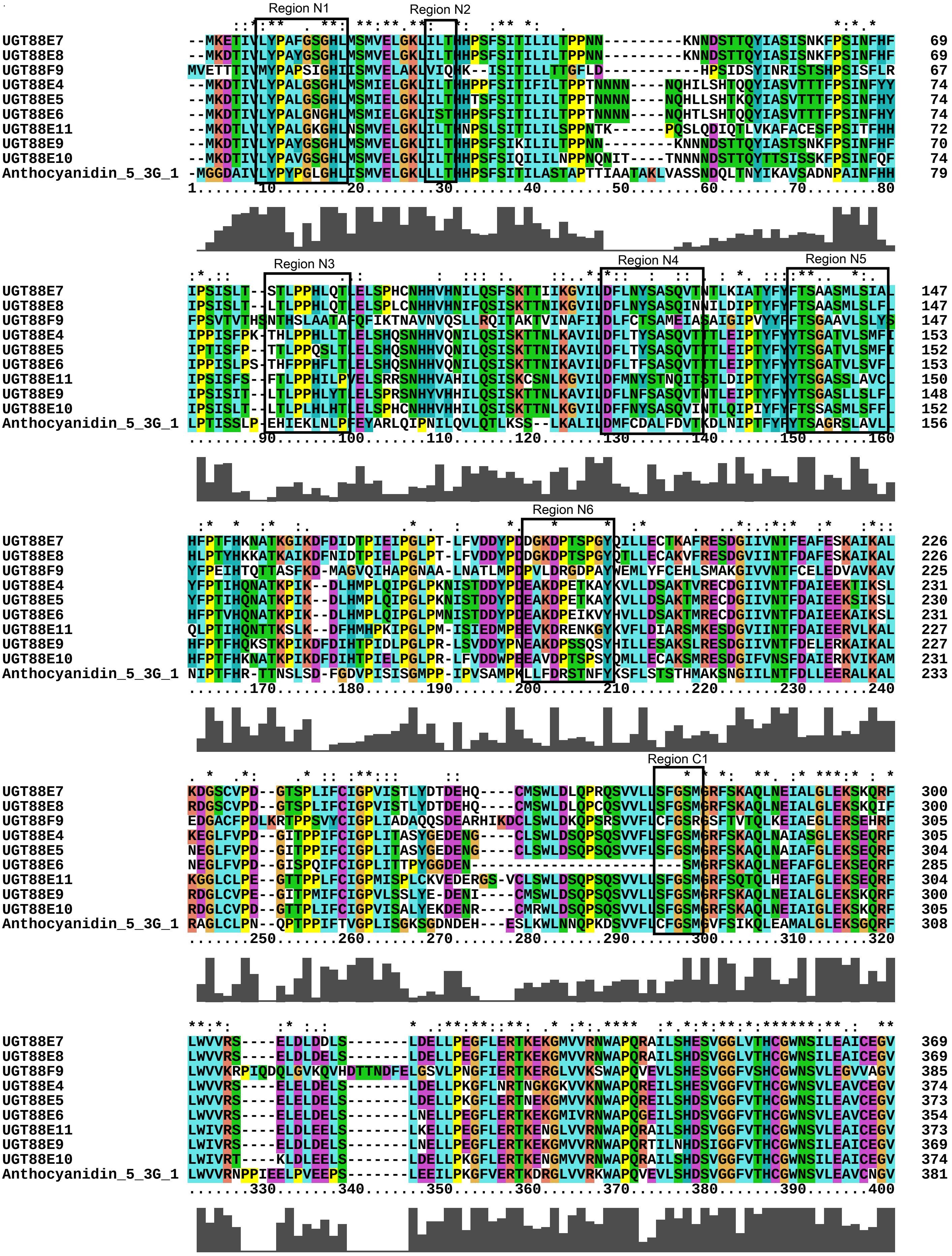
Region N1
Region N2
UGT88E7 --MKETIVLYPAFGSGHLMSMVELGKLILTHHPSFSITILILTPPNN---------KNNDSTTQYIASISNKFPSINFHF 69
UGT88E8 --MKDTIVLYPAYGSGHLMSMVELGKLILTHHPSFSITILILTPPNN---------KNNDSTTQYIASISNKFPSINFHF 69
UGT88F9 MVETTTIVMYPAPSIGHIISMVELAKLVIQHK--ISITILLTTGFLD-----------HPSIDSYINRISTSHPSISFLR 67
UGT88E4 --MKDTIVLYPALGSGHLMSMIELGKLILTHHPPFSITIFILTPPTNNNN----NQHILSHTQQYIASVTTTFPSINFYY 74
UGT88E5 --MKDTIVLYPALGSGHLMSMIELGKLILTHHTSFSITILILTPPTNNNN----NQHLLSHTKQYIASVTTTFPSINFHY 74
UGT88E6 --MKDTIVLYPALGNGHLMSMIELGKLISTHHPSFSITILILTPPTNNNN----NQHTLSHTQQYIASVTTTFPSINFHY 74
UGT88E11 --MKDTLVLYPALGKGHLNSMVELGKLILTHNPSLSITILILSPPNTK------PQSLQDIQTLVKAFACESFPSITFHH 72
UGT88E9 --MKDTIVLYPAYGSGHLMSMVELGKLILTHHPSFSIKILILTPPNN--------KNNNDSTTQYIASTSNKFPSINFHF 70
UGT88E10 --MKDTIVLYPAVGSGHLMSMVELGKLILTHHPSFSIQILILNPPNQNIT----TNNNNDSTTQYTTSISSKFPSINFQF 74
Anthocyanidin_5_3G_1 -MGGDAIVLYPYPGLGHLISMVELGKLLLTHHPSFSITILASTAPTTIAATAKLVASSNDQLTNYIKAVSADNPAINFHH 79
1.......10........20........30........40........50........60........70........80
Region N3
Region N4
Region N5
UGT88E7 IPSISLT--STLPPHLQTLELSPHCNHHVHNILQSFSKTTIIKGVILDFLNYSASQVTNTLKIATYFYFTSAASMLSIAL 147
UGT88E8 IPSISLT--LTLPPHLQTLELSPLCNHHVHNIFQSISKTTNIKGVILDFLNYSASQINNILDIPTYFYFTSAASMLSLFL 147
UGT88F9 FPSVTVTHSNTHSLAATAFQFIKTNAVNVQSLLRQITAKTVINAFIIDLFCTSAMEIASAIGIPVYYFFTSGAAVLSLYS 147
UGT88E4 IPPISFPK-THLPPHLLTLELSHQSNHHVQNILQSISKTTNLKAVILDFLTYSASQVTTTLEIPTYFYYTSGATVLSMFI 153
UGT88E5 IPTISFP--TTLPPQSLTLELSHQSNHHVQNILQSISKTTNIKAVILDFLTYSASQVTTTLEIPTYFYYTSGATVLSMFI 152
UGT88E6 IPPISLPS-THFPPHFLTLELSHQSNHHVQNILQSISKTTNIKAVILDFLTFSASQVTTTLEIPTYFYYTSGATVLSVFL 153
UGT88E11 IPSISFS--FTLPPHILPVELSRRSNHHVAHILQSISKCSNLKGVILDFMNYSTNQITSTLDIPTYFYYTSGASSLAVCL 150
UGT88E9 IPSISIT--LTLPPHLYTLELSPRSNHYVHHILQSISKTTNLKAVILDFLNFSASQVTNTLEIPTYFYYTSGASLLSLFL 148
UGT88E10 IPSISLT--LTLPLHLHTLELSPHCNHHVHHILQSISKTTNLKGVILDFFNYSASQVINTLQIPIYFYFTSSASMLSFFL 152
Anthocyanidin_5_3G_1 LPTISSLP-EHIEKLNLPFEYARLQIPNILQVLQTLKSS--LKALILDMFCDALFDVTKDLNIPTFYFYTSAGRSLAVLL 156
........90........100.......110.......120.......130.......140.......150.......160
Region N6
UGT88E7 HFPTFHKNATKGIKDFDIDTPIEIPGLPT-LFVDDYPDDGKDPTSPGYQILLECTKAFRESDGIIVNTFEAFESKAIKAL 226
UGT88E8 HLPTYHKKATKAIKDFNIDTPIELPGLPT-LFVDDYPDDGKDPTSPGYQTLLECAKVFRESDGVIINTFDAFESKAIKAL 226
UGT88F9 YFPEIHTQTTASFKD-MAGVQIHAPGNAA-LNATLMPDPVLDRGDPAYWEMLYFCEHLSMAKGIVVNTFCELEDVAVKAV 225
UGT88E4 YFPTIHQNATKPIK--DLHMPLQIPGLPKNISTDDYPDEAKDPETKAYKVLLDSAKTVRECDGIIVNTFDAIEEKTIKSL 231
UGT88E5 YFPTIHQNATKPIK--DLHMPLQIPGLPKNISTDDYPDEAKDPETKAYKVLLDSAKTMRECDGIIVNTFDAIEEKSIKSL 230
UGT88E6 HFPTVHQNATKPIK--DLHMPLQIPGLPMNISTDDYPDEAKDPEIKVYHVLLDSAKTMRECDGIIVNTFDAIEEKAIKSL 231
UGT88E11 QLPTIHQNTTKSLK--DFHMHPKIPGLPM-ISIEDMPEEVKDRENKGYKVFLDIARSMKESDGVIINTFDAIEERVLKAL 227
UGT88E9 HFPTFHQKSTKPIKDFDIHTPIDLPGLPR-LSVDDYPNEAKDPSSQSYHILLESAKSLRESDGIIVNTFDELERKAIKAL 227
UGT88E10 HFPTFHKNATKPIKDFDIHTPIELPGLPR-LFVDDWPEEAVDPTSPSYQMLLECAKSMRESDGIFVNSFDAIERKVIKAM 231
Anthocyanidin_5_3G_1 NIPTFHR-TTNSLSD-FGDVPISISGMPP-IPVSAMPKLLFDRSTNFYKSFLSTSTHMAKSNGIILNTFDLLEERALKAL 233
.......170.......180.......190.......200.......210.......220.......230.......240
Region C1
UGT88E7 KDGSCVPD--GTSPLIFCIGPVISTLYDTDEHQ----CMSWLDLQPRQSVVLLSFGSMGRFSKAQLNEIALGLEKSKQRF 300
UGT88E8 RDGSCVPD--GTSPLIFCIGPVISTLYDTDEHQ----CMSWLDLQPCQSVVLLSFGSMGRFSKAQLNEIALGLEKSKQIF 300
UGT88F9 EDGACFPDLKRTPPSVYCIGPLIADAQQSDEARHIKDCLSWLDKQPSRSVVFLCFGSRGSFTVTQLKEIAEGLERSEHRF 305
UGT88E4 KEGLFVPD--GITPPIFCIGPLITASYGEDENG----CLSWLDSQPSQSVVFLSFGSMGRFSKAQLNAIASGLEKSEQRF 305
UGT88E5 NEGLFVPD--GITPPIFCIGPLITASYGEDENG----CLSWLDSQPSQSVVFLSFGSMGRFSKAQLNAIAFGLEKSEQRF 304
UGT88E6 NEGLFVPD--GISPQIFCIGPLITTPYGGDEN--------------------------SMGRFSKAQLNEFAFGLEKSEQRF 285
UGT88E11 KGGLCLPE--GTTPPLFCIGPMISPLCKVEDERGS-VCLSWLDSQPSQSVVLLSFGSMGRFSQTQLHEIAFGLEKSEQRF 304
UGT88E9 RDGLCVPE--GITPMIFCIGPVLSSLYE-DENI----CMSWLDSQPSQSVVLLSFGSMGRFSKAQLNEIALGLEKSKQRF 300
UGT88E10 RDGLCVPD--GTTPLIFCIGPVISALYEKDENR----CMRWLDSQPSQSVVLLSFGSMGRFSKAQLNEIAIGLEKSKQRF 305
Anthocyanidin_5_3G_1 RAGLCLPN--QPTPPIFTVGPLISGKSGDNDEH---ESLKWLNNQPKDSVVFLCFGSMGVFSIKQLEAMALGLEKSGQRF 308
.......250.......260.......270.......280.......290.......300.......310.......320
UGT88E7 LWVVRS----ELDLDDLS-------LDELLPEGFLERTKEKGMVVRNWAPQRAILSHESVGGLVTHCGWNSILEAICEGV 369
UGT88E8 LWVVRS----ELDLDELS-------LDELLPEGFLERTKEKGMVVRNWAPQRAILSHDSVGGLVTHCGWNSILEAICEGV 369
UGT88F9 LWVVKRPIQDQLGVKQVHDTTNDFELGSVLPNGFIERTKERGLVVKSWAPQVEVLSHESVGGFVSHCGWNSVLEGVVAGV 385
UGT88E4 LWVVRS----ELELDELS-------LDELLPKGFLNRTNGKGKVVKNWAPQREILSHESVGGFVTHCGWNSVLEAVCEGV 374
UGT88E5 LWVVRS----ELELDELS-------LDELLPKGFLERTNEKGMVVKNWAPQREILSHDSVGGFVTHCGWNSILEAVCEGV 373
UGT88E6 LWVVRS----ELELDELS-------LNELLPEGFLERTKEKGMIVRNWAPQGEILSHDSVGGFVTHCGWNSVLEAVCEGV 354
UGT88E11 LWIVRS----ELDLEELS-------LKELLPEGFLERTKENGLVVRNWAPQRAILSHDSVGGFVTHCGWNSVLEAICEGV 373
UGT88E9 LWIVRS----ELDLDELS-------LNELLPEGFLERTKEKGMVVRNWAPQRTILNHDSIGGFVTHCGWNSILEAICEGV 369
UGT88E10 LWIVRT----KLDLEELS-------LDELLPKGFLERTKENGMVVRNWAPQRAILSHDSVGGFVTHCGWNSILEAICEGV 374
Anthocyanidin_5_3G_1 LWVVRNPPIEELPVEEPS-------LEEILPKGFVERTKDRGLVVRKWAPQVEVLSHDSVGGFVTHCGWNSVLEAVCNGV 381
.......330.......340.......350.......360.......370.......380.......390.......400

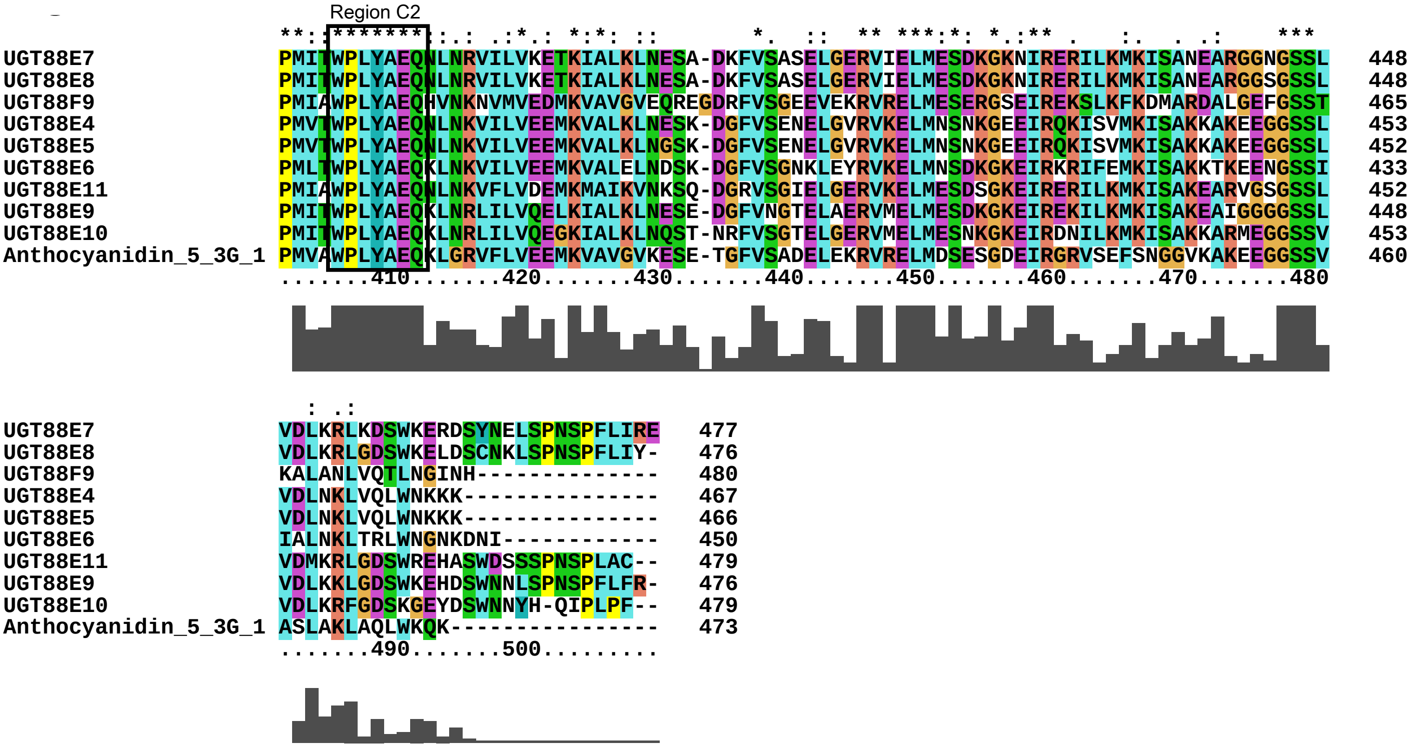
Region C2
:.*****:.: .:*.: *:*: ::   *. :: ** ***:*: *.:** .   :. . .:   ***
UGT88E7 PMITWPLYAEQNLNRVILVKETKIALKLNESA-DKFVSASELGERVIELMESDKGKNIRERILKMKISANEARGGNGSSL 448
UGT88E8 PMITWPLYAEQNLNRVILVKETKIALKLNESA-DKFVSASELGERVIELMESDKGKNIRERILKMKISANEARGGSGSSL 448
UGT88F9 PMIAWPLYAEQHVNKNVMVEDMKVAVGVEQREGDRFVSGEEVEKRVRELMESERGSEIREKSLKFKDMARDALGEFGSST 465
UGT88E4 PMVTWPLYAEQNLNKVILVEEMKVALKLNESK-DGFVSENELGVRVKELMNSNKGEEIRQKISVMKISAKKAKEEGGSSL 453
UGT88E5 PMVTWPLYAEQNLNKVILVEEMKVALKLNGSK-DGFVSENELGVRVKELMNSNKGEEIRQKISVMKISAKKAKEEGGSSL 452
UGT88E6 PMLTWPLYAEQKLNKVILVEEMKVALELNDSK-DGFVSGNKLEYRVKELMNSDKGKEIRKRIFEMKISAKKTKEENGSSI 433
UGT88E11 PMIAWPLYAEQNLNKVFLVDEMKMAIKVNKSQ-DGRVSGIELGERVKELMESDSGKEIRERILKMKISAKEARVGSGSSL 452
UGT88E9 PMITWPLYAEQKLNRLILVQELKIALKLNESE-DGFVNGTELAERVMELMESDKGKEIREKILKMKISAKEAIGGGGSSL 448
UGT88E10 PMITWPLYAEQKLNRLILVQEGKIALKLNQST-NRFVSGTELGERVMELMESNKGKEIRDNILKMKISAKKARMEGGSSV 453
Anthocyanidin_5_3G_1 PMVAWPLYAEQKLGRVFLVEEMKVAVGVKESE-TGFVSADELEKRVRELMDSESGDEIRGRVSEFSNGGVKAKEEGGSSV 460
.......410.......420.......430.......440.......450.......460.......470.......480
: .:
UGT88E7 VDLKRLKDSWKERDSYNELSPNSPFLIRE 477
UGT88E8 VDLKRLGDSWKELDSCNKLSPNSPFLIY- 476
UGT88F9 KALANLVQTLNGINH-------------- 480
UGT88E4 VDLNKLVQLWNKKK--------------- 467
UGT88E5 VDLNKLVQLWNKKK--------------- 466
UGT88E6 IALNKLTRLWNGNKDNI------------ 450
UGT88E11 VDMKRLGDSWREHASWDSSSPNSPLAC-- 479
UGT88E9 VDLKKLGDSWKEHDSWNNLSPNSPFLFR- 476
UGT88E10 VDLKRFGDSKGEYDSWNNYH-QIPLPF-- 479
Anthocyanidin_5_3G_1 ASLAKLAQLWKQK---------------- 473
.......490.......500.........

Group G

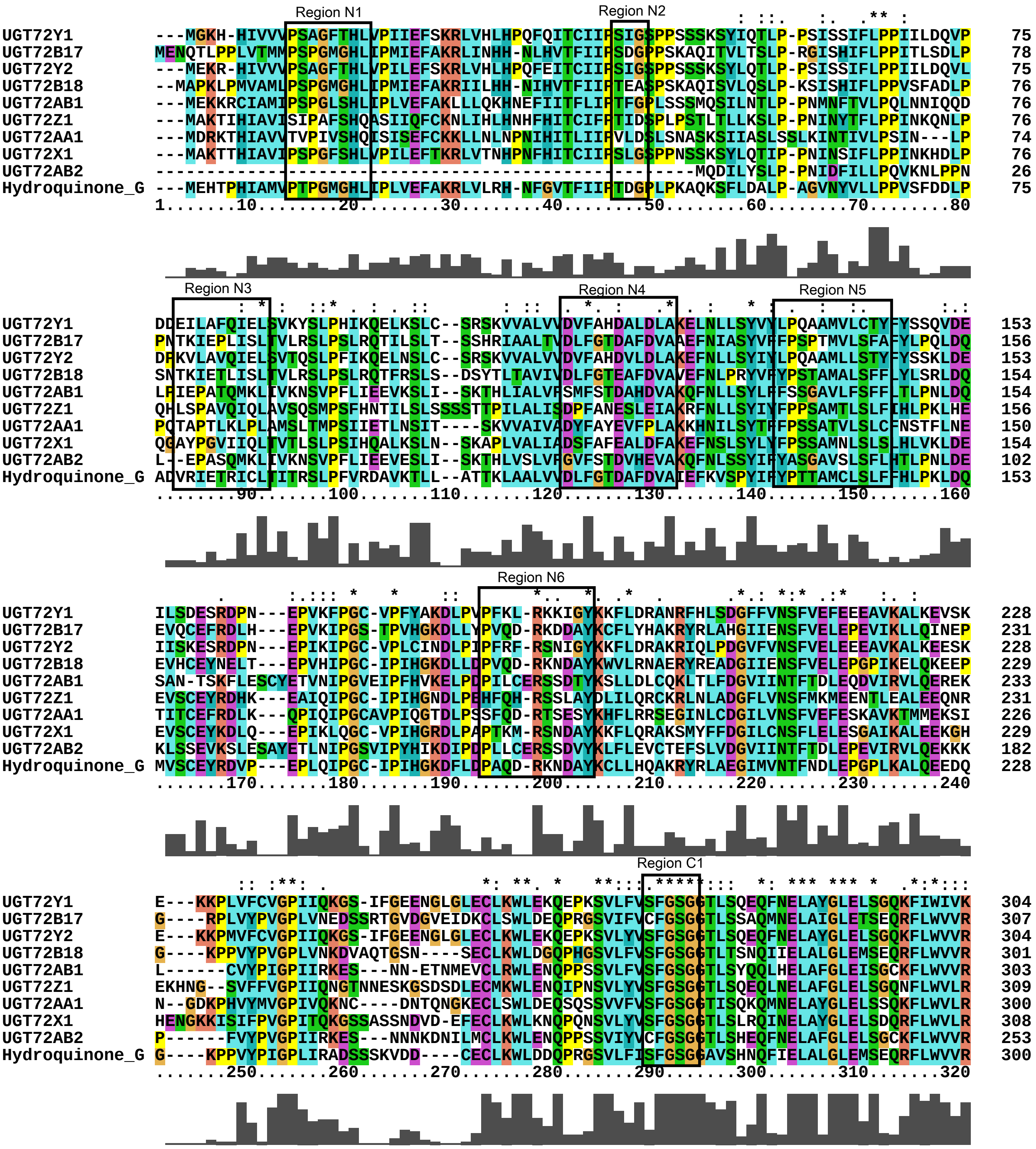
Region N1
Region N2
UGT72Y1 ---MGKH-HIVVVPSAGFTHLVPIIEFSKRLVHLHPQFQITCIIPSIGSPPSSSKSYIQTLP-PSISSIFLPPIILDQVP 75
UGT72B17 MENQTLPPLVTMMPSPGMGHLIPMIEFAKRLINHH-NLHVTFIIPSDGPPSKAQITVLTSLP-RGISHIFLPPITLSDLP 78
UGT72Y2 ---MEKR-HIVVVPSAGFTHLVPILEFSKRLVHLHPQFEITCIIPSIGSPPSSSKSYLQTLP-PSISSIFLPPIILDQVL 75
UGT72B18 --MAPKLPMVAMLPSPGMGHLIPMIEFAKRIILHH-NIHVTFIIPTEASPSKAQISVLQSLP-KSISHIFLPPVSFADLP 76
UGT72AB1 ---MEKKRCIAMIPSPGLSHLIPLVEFAKLLLQKHNEFIITFLIPTFGPLSSSMQSILNTLP-PNMNFTVLPQLNNIQQD 76
UGT72Z1 ---MAKTIHIAVISIPAFSHQASIIQFCKNLIHLHNHFHITCIFPTIDSPLPSTLTLLKSLP-PNINYTFLPPINKQNLP 76
UGT72AA1 ---MDRKTHIAVVTVPIVSHQISISEFCKKLLNLNPNIHITLIIPVLDSLSNASKSIIASLSSLKINTIVLPSIN---LP 74
UGT72X1 ---MAKTTHIAVIPSPGFSHLVPILEFTKRLVTNHPNFHITCIIPSLGSPPNSSKSYLQTIP-PNINSIFLPPINKHDLP 76
UGT72AB2 ---MQDILYSLP-PNIDFILLPQVKNLPPN 26
Hydroquinone_G ---MEHTPHIAMVPTPGMGHLIPLVEFAKRLVLRH-NFGVTFIIPTDGPLPKAQKSFLDALP-AGVNYVLLPPVSFDDLP 75
1.......10........20........30........40........50........60........70........80
Region N3
Region N4
Region N5
UGT72Y1 DDEILAFQIELSVKYSLPHIKQELKSLC--SRSKVVALVVDVFAHDALDLAKELNLLSYVYLPQAAMVLCTYFYSSQVDE 153
UGT72B17 PNTKIEPLISLTVLRSLPSLRQTILSLT--SSHRIAALTVDLFGTDAFDVAAEFNIASYVFFPSPTMVLSFAFYLPQLDQ 156
UGT72Y2 DFKVLAVQIELSVTQSLPFIKQELNSLC--SRSKVVALVVDVFAHDVLDLAKEFNLLSYIYLPQAAMLLSTYFYSSKLDE 153
UGT72B18 SNTKIETLISLTVLRSLPSLRQTFRSLS--DSYTLTAVIVDLFGTEAFDVAVEFNLPRYVFYPSTAMALSFFLYLSRLDQ 154
UGT72AB1 LPIEPATQMKLIVKNSVPFLIEEVKSLI--SKTHLIALVPSMFSTDAHDVAKQFNLLSYLFFSSGAVLFSFFLTLPNLDQ 154
UGT72Z1 QHLSPAVQIQLAVSQSMPSFHNTILSLSSSSTTPILALISDPFANESLEIAKRFNLLSYIYFPPSAMTLSLFIHLPKLHE 156
UGT72AA1 PQTAPTLKLPLAMSLTMPSIIETLNSIT----SKVVAIVADYFAYEVFPLAKKHNILSYTFFPSSATVLSLCFNSTFLNE 150
UGT72X1 QGAYPGVIIQLTVTLSLPSIHQALKSLN--SKAPLVALIADSFAFEALDFAKEFNSLSYLYFPSSAMNLSLSLHLVKLDE 154
UGT72AB2 L--EPASQMKLIVKNSVPFLIEEVESLI--SKTHLVSLVPGVFSTDVHEVAKQFNLSSYIFYASGAVSLSFLHTLPNLDE 102
Hydroquinone_G ADVRIETRICLTITRSLPFVRDAVKTLL--ATTKLAALVVDLFGTDAFDVAIEFKVSPYIFYPTTAMCLSLFFHLPKLDQ 153
........90.......100.......110.......120.......130.......140.......150.......160
Region N6
UGT72Y1 ILSDESRDPN---EPVKFPGC-VPFYAKDLPVPFKL-RKKIGYKKFLDRANRFHLSDGFFVNSFVEFEEEAVKALKEVSK 228
UGT72B17 EVQCEFRDLH---EPVKIPGS-TPVHGKDLLYPVQD-RKDDAYKCFLYHAKRYRLAHGIIENSFVELEPEVIKLLQINEP 231
UGT72Y2 IISKESRDPN---EPIKIPGC-VPLCINDLPIPFRF-RSNIGYKKFLDRAKRIQLPDGVFVNSFVELEEEAVKALKEESK 228
UGT72B18 EVHCEYNELT---EPVHIPGC-IPIHGKDLLDPVQD-RKNDAYKWVLRNAERYREADGIIENSFVELEPGPIKELQKEEP 229
UGT72AB1 SAN-TSKFLESCYETVNIPGVEIPFHVKELPDPILCERSSDTYKSLLDLCQKLTLFDGVIINTFTDLEQDVIRVLQEREK 233
UGT72Z1 EVSCEYRDHK---EAIQIPGC-IPIHGNDLPEHFQH-RSSLAYDLILQRCKRLNLADGFLVNSFMKMEENTLEALEEQNR 231
UGT72AA1 TITCEFRDLK---QPIQIPGCAVPIQGTDLPSSFQD-RTSESYKHFLRRSEGINLCDGILVNSFVEFESKAVKTMMEKSI 226
UGT72X1 EVSCEYKDLQ---EPIKLQGC-VPIHGRDLPAPTKM-RSNDAYKKFLQRAKSMYFFDGILCNSFLELESGAIKALEEKGH 229
UGT72AB2 KLSSEVKSLESAYETLNIPGSVIPYHIKDIPDPLLCERSSDVYKLFLEVCTEFSLVDGVIINTFTDLEPEVIRVLQEKKK 182
Hydroquinone_G MVSCEYRDVP---EPLQIPGC-IPIHGKDFLDPAQD-RKNDAYKCLLHQAKRYRLAEGIMVNTFNDLEPGPLKALQEEDQ 228
.......170.......180.......190.......200.......210.......220.......230.......240
Region C1
UGT72Y1 E---KKPLVFCVGPIIQKGS-IFGEENGLGLECLKWLEKQEPKSVLFVSFGSGGTLSQEQFNELAYGLELSGQKFIWIVK 304
UGT72B17 G----RPLVYPVGPLVNEDSSRTGVDGVEIDKCLSWLDEQPRGSVIFVCFGSGGTLSSAQMNELAIGLETSEQRFLWVVR 307
UGT72Y2 E---KKPMVFCVGPIIQKGS-IFGEENGLGLECLKWLEKQEPKSVLYVSFGSGGTLSQEQFNELAYGLELSGQKFLWVVR 304
UGT72B18 G----KPPVYPVGPLVNKDVAQTGSN----SECLKWLDGQPHGSVLFVSFGSGGTLTSNQIIELALGLEMSEQRFLWVVR 301
UGT72AB1 L------CVYPIGPIIRKES---NN-ETNMEVCLRWLENQPPSSVLFVSFGSGGTLSYQQLHELAFGLEISGCKFLWVVR 303
UGT72Z1 EKHNG--SVFFVGPIIQNGTNNESKGSDSDLECMKWLENQIPNSVLYVSFGSGGTLSQEQLNELAFGLELSGQNFLWVLR 309
UGT72AA1 N--GDKPHVYMVGPIVQKNC----DNTQNGKECLSWLDEQSQSSVVFVSFGSGGTISQKQMNELAYGLELSSQKFLWVLR 300
UGT72X1 HENGKKISIFPVGPITQKGSSASSNDVD-EFECLKWLKNQPQNSVLYVSFGSGGTLSLRQINELAYGLELSDQRFLWVLR 308
UGT72AB2 P------FVYPVGPIIRKES---NNNKDNILMCLKWLENQPPSSVIYVCFGSGGTLSHEQFNELAFGLELSGCKFLWVVR 253
Hydroquinone_G G----KPPVYPIGPLIRADSSSKVDD----CECLKWLDDQPRGSVLFISFGSGGAVSHNQFIELALGLEMSEQRFLWVVR 300
.......250.......260.......270.......280.......290.......300.......310.......320

Group H

```
                  *.      .    :        *.: :** **:***: : :::  *.** ::* : : ****:**** * **.:   .:*::.*
UGT72Y1          EPNG-VANASYFGGEI--EDPLNFLPIGFLERTKEQGFVVPSWGPQIQILGHSSTGGFLSHCGWNSVLESVVYGVPIIAW    381
UGT72B17         CPKDKVANVSNFIADSN-VDPFDFLPNGFVERTKEKGLVVPYWAPQAKVLNHGSTGGFLTHCGWNSVLESVVNGVPLVAW    386
UGT72Y2          EPSG-VANASYFGGEI--KDPLKFLPNGFLERTKEQGFVVPSWGPQIQILGHSSTGGFLSHCGWNSVLESVVYGVPIIAW    381
UGT72B18         SPNDKVANASYFSADTQ-ADPFDFLPKGFLERTNKRGLVVSSWAPQPQVLAHGSTGGFLTHCGWNSILESVVNAVPLVVW    380
UGT72AB1         VPSK-NSSSAYFSKQN--DDPLEYLPNGFLERTKDKGLVVASWAPQVEILVHESIGGFLSHCGWSSTLESVVNGVPMIAW    380
UGT72Z1          APSD-TSNEAYLGVKNN-DDPLNFLPKGFIERTKGKGFVIVNWGPQTQILSHISVGGFLTHCGWNSVLESVVLGVPMIAW    387
UGT72AA1         EPKD-IASANYFGVSSIQEDPLSFLPKGFLERTKKQGFLVSNWAPQVNILSHKAIGGFLTHCGWFSTLECVVNGLPIIAW    379
UGT72X1          APSD-SVSADYFEDAN--VDPLKFLPKGFLERTKEKGLVLASWAPQVEVLKQSSVGGFLSHCGWNSILESIQEGVPIVAW    385
UGT72AB2         VPSN-DVSSAYFVGKK--EDPLEYLPNGFLERTKDKGLVVPSWAPQVEILGHESIGGFLSHCGWSSTLESVVNGVPMIAW    330
Hydroquinone_G   SPNDKIANATYFSIQNQ-NDALAYLPEGFLERTKGRCLLVPSWAPQTEILSHGSTGGFLTHCGWNSILESVVNGVPLIAW    379
                 .......330.......340.......350.......360.......370.......380.......390.......400
```

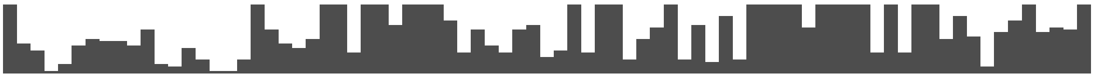

```
                 Region C2
                 **:*:* : *  : : ::*.:* :    :. :: : :: ..:: :     :    :    :  *    *  ::    *.*   :
UGT72Y1          PLFADQGVNAAMLSDGVKVALRPEVN-DNGLVERNEIDKVVRELMEGEKGVEIRKRMEHLKNAAAVAINEMGPSTKALSE    460
UGT72B17         PLYAEQKMNAVLVSEEVKVAMRPKVS-ENGLVERDEIANVVKRLMEGEEGKKLRNKMMDLKEAASIALKENGSSTKKICE    465
UGT72Y2          PLFAEQGMNAVMLCDGLKVALRAKVN-ENGLIERDEIAKVVRELLEGEEGVEICKRMEHLKSVAADAIDEMGSSTKTLSE    460
UGT72B18         PLYAEQKMNAVILTEDVKVALRPNVG-ENGLVERQEIASVVKCLMEGEEGKKLRYQMKDLKDAAVKTLGENGTSTNYISK    459
UGT72AB1         PLFAEQKMNAKELVDVLKVGVRAKVDDENGIVKRDEVVKAIKGIMEGDESLEIRKRIKELSVGAANALSEHGSSMKALSD    460
UGT72Z1          PLFAEQKLNAVLLCDGLKVAIRPKIN-ENGLVEREEIVKVIKGVMLDGEENGIRERIGELRDGAVDALKDNGSSTRAILE    466
UGT72AA1         PLFAEQRMIATILADELKVAIRPKVDHESGIVEKGEISNVLKRLMVEDEGIKICERMKFLQYAAAGAIEVDGSSTTTLSQ    459
UGT72X1          PLFAEQAMNAVMLSDGLEVAIRLKFE-DDEIVEKEKIAKVVKCLMEGEEGKGIRERMKVLKDGAAKALKDDGSSIQTLSY    464
UGT72AB2         PLFAEQRMNARELVDVLKVGVRAKVDDENGIVKRDEVVKAIKRIKEGDESLEIKNRIKELSVSAANALREHGSSKKALSD    410
Hydroquinone_G   PLYAEQKMNAVMLTEGLKVALRPKAG-ENGLIGRVEIANAVKGLMEGEEGKKFRSTMKDLKDAASRALSDDGSSTKALAE    458
                 .......410.......420.......430.......440.......450.......460.......470.......480
```

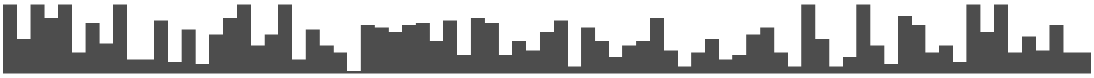

```
                 .
UGT72Y1          VADVWKGI------     468
UGT72B17         LALKWKGTQTLSNV     479
UGT72Y2          VADIWKDI------     468
UGT72B18         LALKWSNKSNVTN-     472
UGT72AB1         LVVKLHDY------     468
UGT72Z1          FGNCLKRFGRNI--     478
UGT72AA1         LVTKWTNLEGCNES     473
UGT72X1          LANQWENFG-GI--     475
UGT72AB2         LALRWHI-------     417
Hydroquinone_G   LACKWENKISST--     470
                 .......490....
```

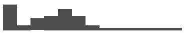

Group H

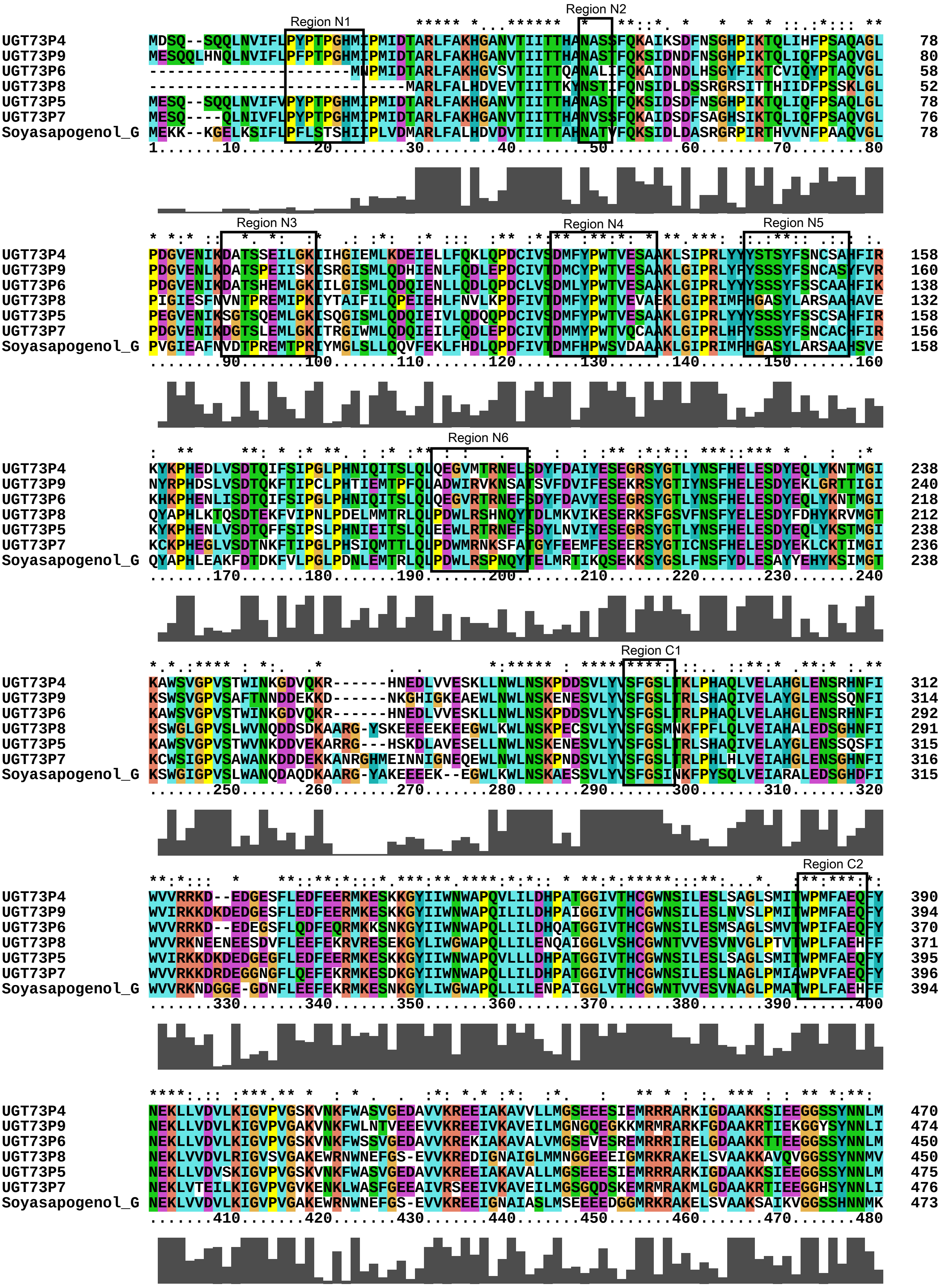

Region N1
Region N2
Region N3
Region N4
Region N5
Region N6
Region C1
Region C2
UGT73P4
UGT73P9
UGT73P6
UGT73P8
UGT73P5
UGT73P7
Soyasapogenol_G

```
                  :*: **..:* ::
UGT73P4           QLIDELKSLKISRGLEKTN---   489
UGT73P9           QLLDELTSLKIARELEKSRLDN   496
UGT73P6           QLIDELKSLKLSRGLEEQ----   468
UGT73P8           ELIQELKSIKLAKVQA------   466
UGT73P5           QLIDELKSLKISRGLEKTN---   494
UGT73P7           QLIDELKSLKKSKALAVKED--   496
Soyasapogenol_G   ELIRELKEIKLSKEAQETAPNP   495
                  .......490.......500..
```

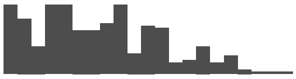

Group I

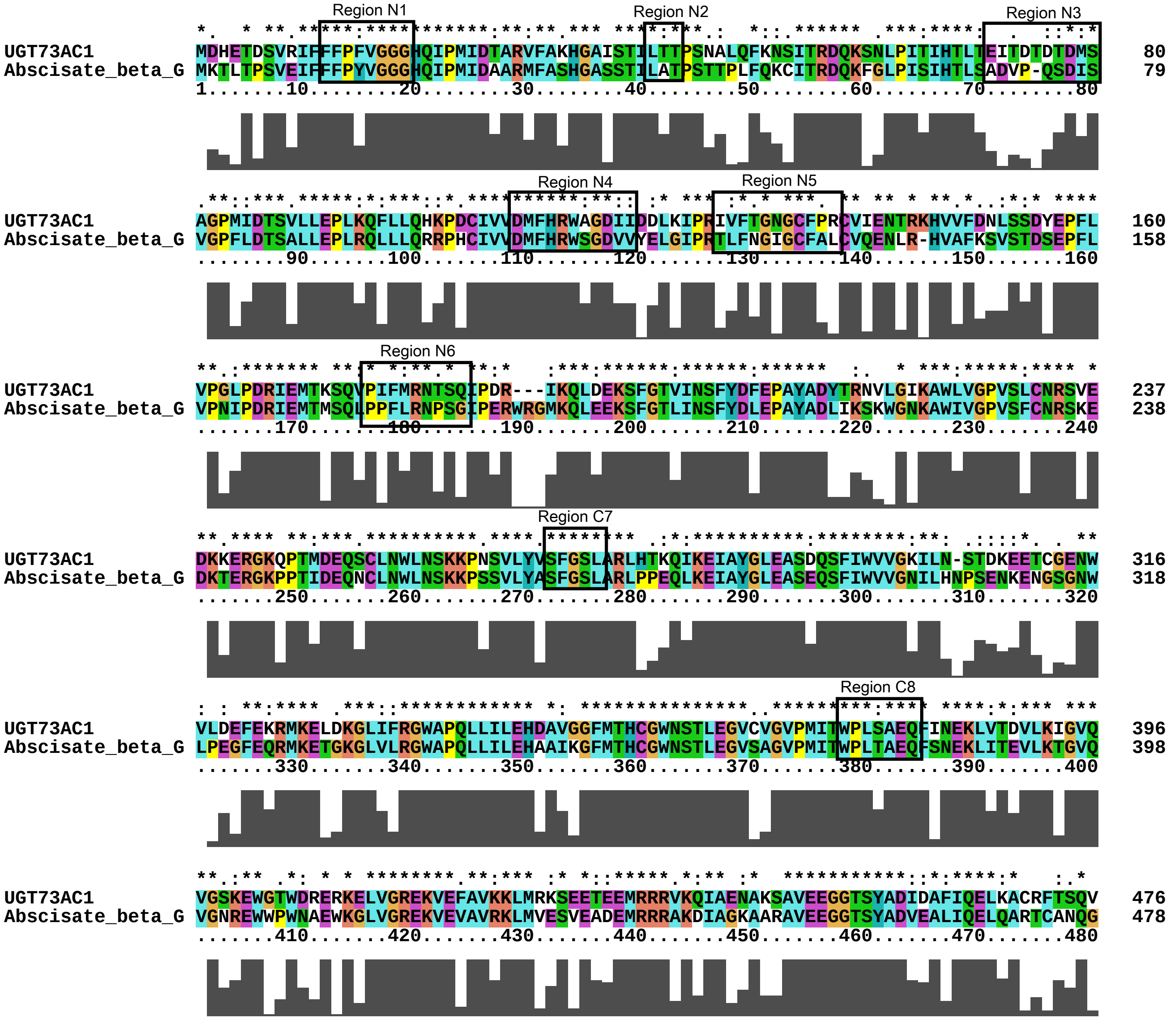
Region N1
Region N2
Region N3
Region N4
Region N5
Region N6
Region C7
Region C8
UGT73AC1
Abscisate_beta_G

Group A3 & J

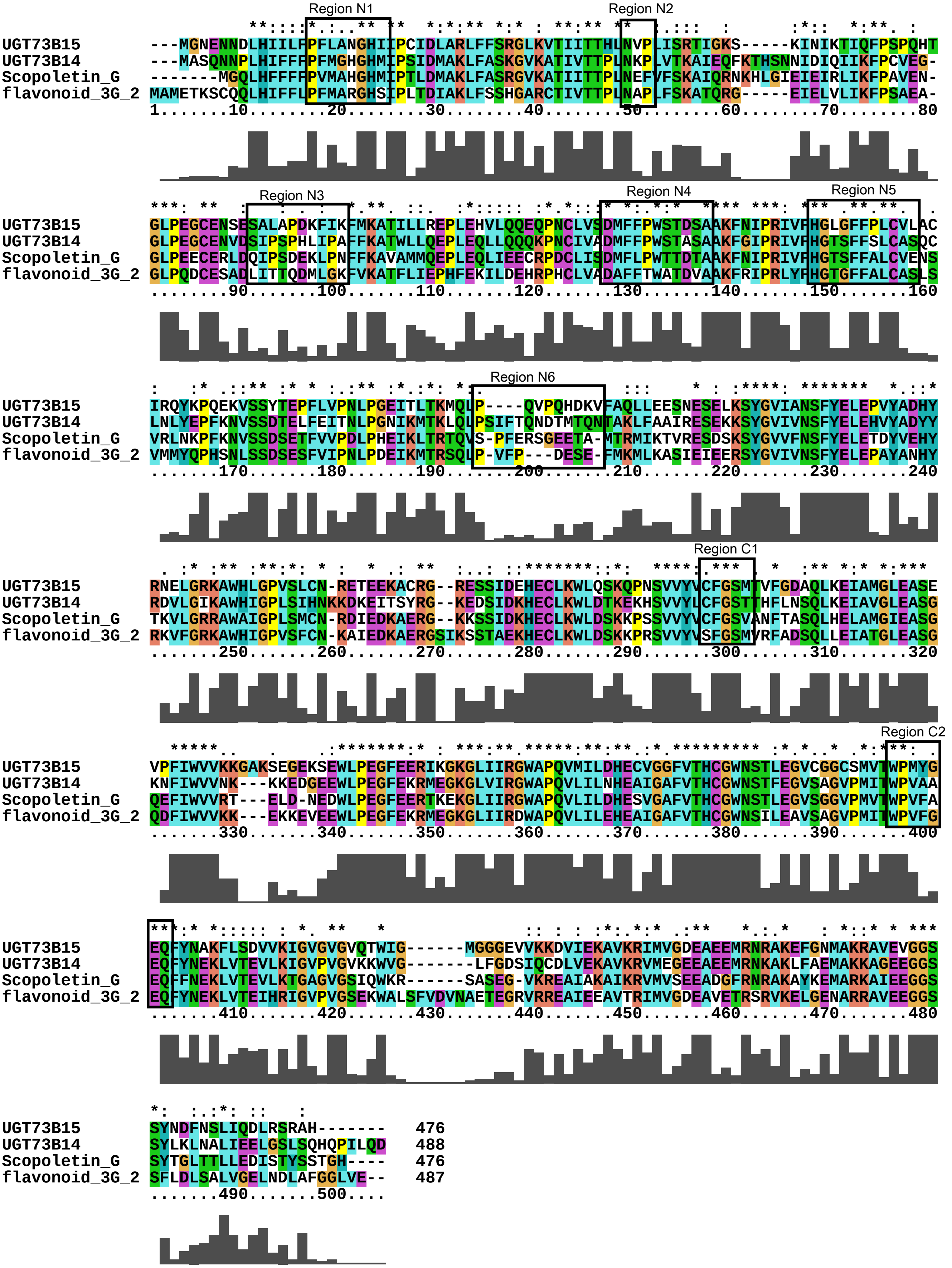
Region N1
Region N2
UGT73B15 ---MGNENNDLHIILFPFLANGHIIPCIDLARLFFSRGLKVTIITTHLNVPLISRTIGKS-----KINIKTIQFPSPQHT 72
UGT73B14 ---MASQNNPLHIFFFPFMGHGHMIPSIDMAKLFASKGVKATIVTTPLNKPLVTKAIEQFKTHSNNIDIQIIKFPCVEG- 76
Scopoletin_G -------MGQLHFFFFPVMAHGHMIPTLDMAKLFASRGVKATIITTPLNEFVFSKAIQRNKHLGIEIEIRLIKFPAVEN- 72
flavonoid_3G_2 MAMETKSCQQLHIFFLPFMARGHSIPLTDIAKLFSSHGARCTIVTTPLNAPLFSKATQRG-----EIELVLIKFPSAEA- 74
Region N3
Region N4
Region N5
UGT73B15 GLPEGCENSESALAPDKFIKFMKATILLREPLEHVLQQEQPNCLVSDMFFPWSTDSAAKFNIPRIVFHGLGFFPLCVLAC 152
UGT73B14 GLPEGCENVDSIPSPHLIPAFFKATWLLQEPLEQLLQQQKPNCIVADMFFPWSTASAAKFGIPRIVFHGTSFFSLCASQC 156
Scopoletin_G GLPEECERLDQIPSDEKLPNFFKAVAMMQEPLEQLIEECRPDCLISDMFLPWTTDTAAKFNIPRIVFHGTSFFALCVENS 152
flavonoid_3G_2 GLPQDCESADLITTQDMLGKFVKATFLIEPHFEKILDEHRPHCLVADAFFTWATDVAAKFRIPRLYFHGTGFFALCASLS 154
Region N6
UGT73B15 IRQYKPQEKVSSYTEPFLVPNLPGEITLTKMQLP----QVPQHDKVFAQLLEESNESELKSYGVIANSFYELEPVYADHY 228
UGT73B14 LNLYEPFKNVSSDTELFEITNLPGNIKMTKLQLPSIFTQNDTMTQNTAKLFAAIRESEKKSYGVIVNSFYELEHVYADYY 236
Scopoletin_G VRLNKPFKNVSSDSETFVVPDLPHEIKLTRTQVS-PFERSGEETA-MTRMIKTVRESDSKSYGVVFNSFYELETDYVEHY 230
flavonoid_3G_2 VMMYQPHSNLSSDSESFVIPNLPDEIKMTRSQLP-VFP---DESE-FMKMLKASIEIEERSYGVIVNSFYELEPAYANHY 229
Region C1
UGT73B15 RNELGRKAWHLGPVSLCN-RETEEKACRG--RESSIDEHECLKWLQSKQPNSVVYVCFGSMTVFGDAQLKEIAMGLEASE 305
UGT73B14 RDVLGIKAWHIGPLSIHNKKDKEITSYRG--KEDSIDKHECLKWLDTKEKHSVVYLCFGSTTHFLNSQLKEIAVGLEASG 314
Scopoletin_G TKVLGRRAWAIGPLSMCN-RDIEDKAERG--KKSSIDKHECLKWLDSKKPSSVVYVCFGSVANFTASQLHELAMGIEASG 307
flavonoid_3G_2 RKVFGRKAWHIGPVSFCN-KAIEDKAERGSIKSSTAEKHECLKWLDSKKPRSVVYVSFGSMVRFADSQLLEIATGLEASG 308
Region C2
UGT73B15 VPFIWVVKKGAKSEGEKSEWLPEGFEERIKGKGLIIRGWAPQVMILDHECVGGFVTHCGWNSTLEGVCGGCSMVTWPMYG 385
UGT73B14 KNFIWVVNK---KKEDGEEWLPEGFEKRMEGKGLVIRGWAPQVLILNHEAIGAFVTHCGWNSTFEGVSAGVPMITWPVAA 391
Scopoletin_G QEFIWVVRT---ELD-NEDWLPEGFEERTKEKGLIIRGWAPQVLILDHESVGAFVTHCGWNSTLEGVSGGVPMVTWPVFA 383
flavonoid_3G_2 QDFIWVVKK---EKKEVEEWLPEGFEKRMEGKGLIIRDWAPQVLILEHEAIGAFVTHCGWNSILEAVSAGVPMITWPVFG 385
UGT73B15 EQFYNAKFLSDVVKIGVGVGVQTWIG------MGGGEVVKKDVIEKAVKRIMVGDEAEEMRNRAKEFGNMAKRAVEVGGS 459
UGT73B14 EQFYNEKLVTEVLKIGVPVGVKKWVG-------LFGDSIQCDLVEKAVKRVMEGEEAEEMRNKAKLFAEMAKKAGEEGGS 464
Scopoletin_G EQFFNEKLVTEVLKTGAGVGSIQWKR------SASEG-VKREAIAKAIKRVMVSEEADGFRNRAKAYKEMARKAIEEGGS 456
flavonoid_3G_2 EQFYNEKLVTEIHRIGVPVGSEKWALSFVDVNAETEGRVRREAIEEAVTRIMVGDEAVETRSRVKELGENARRAVEEGGS 465
UGT73B15 SYNDFNSLIQDLRSRAH------- 476
UGT73B14 SYLKLNALIEELGSLSQHQPILQD 488
Scopoletin_G SYTGLTTLLEDISTYSSTGH---- 476
flavonoid_3G_2 SFLDLSALVGELNDLAFGGLVE-- 487

Group K

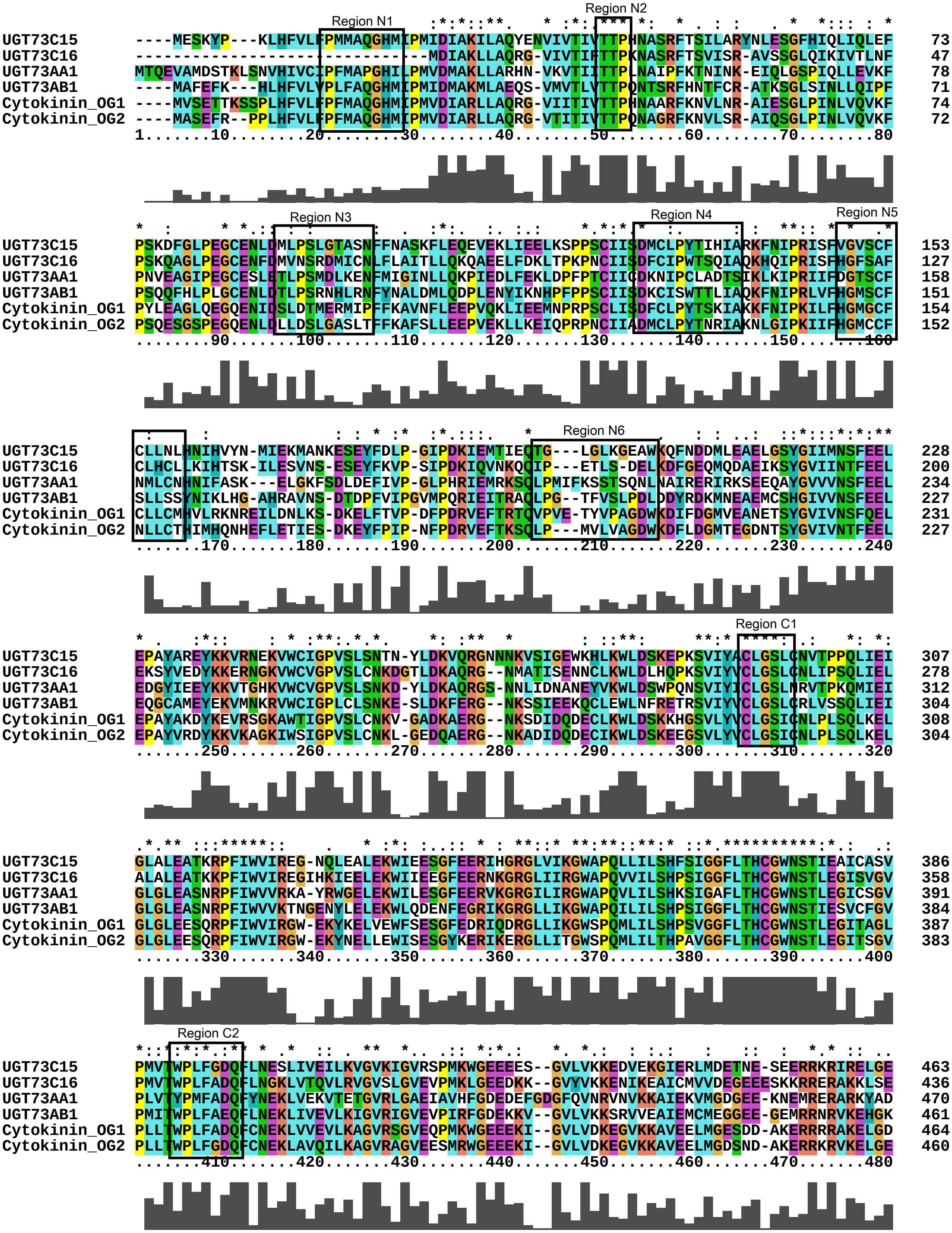
Region N1
Region N2
Region N3
Region N4
Region N5
Region N6
Region C1
Region C2
UGT73C15
UGT73C16
UGT73AA1
UGT73AB1
Cytokinin_OG1
Cytokinin_OG2

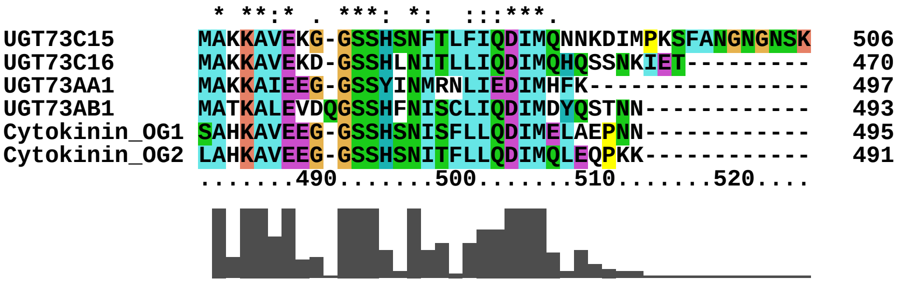
* **:* . ***: *: :::***.
UGT73C15 MAKKAVEKG-GSSHSNFTLFIQDIMQNNKDIMPKSFANGNGNSK 506
UGT73C16 MAKKAVEKD-GSSHLNITLLIQDIMQHQSSNKIET--------- 470
UGT73AA1 MAKKAIEEG-GSSYINMRNLIEDIMHFK---------------- 497
UGT73AB1 MATKALEVDQGSSHFNISCLIQDIMDYQSTNN------------ 493
Cytokinin_OG1 SAHKAVEEG-GSSHSNISFLLQDIMELAEPNN------------ 495
Cytokinin_OG2 LAHKAVEEG-GSSHSNITFLLQDIMQLEQPKK------------ 491
.......490.......500.......510.......520....

Group L

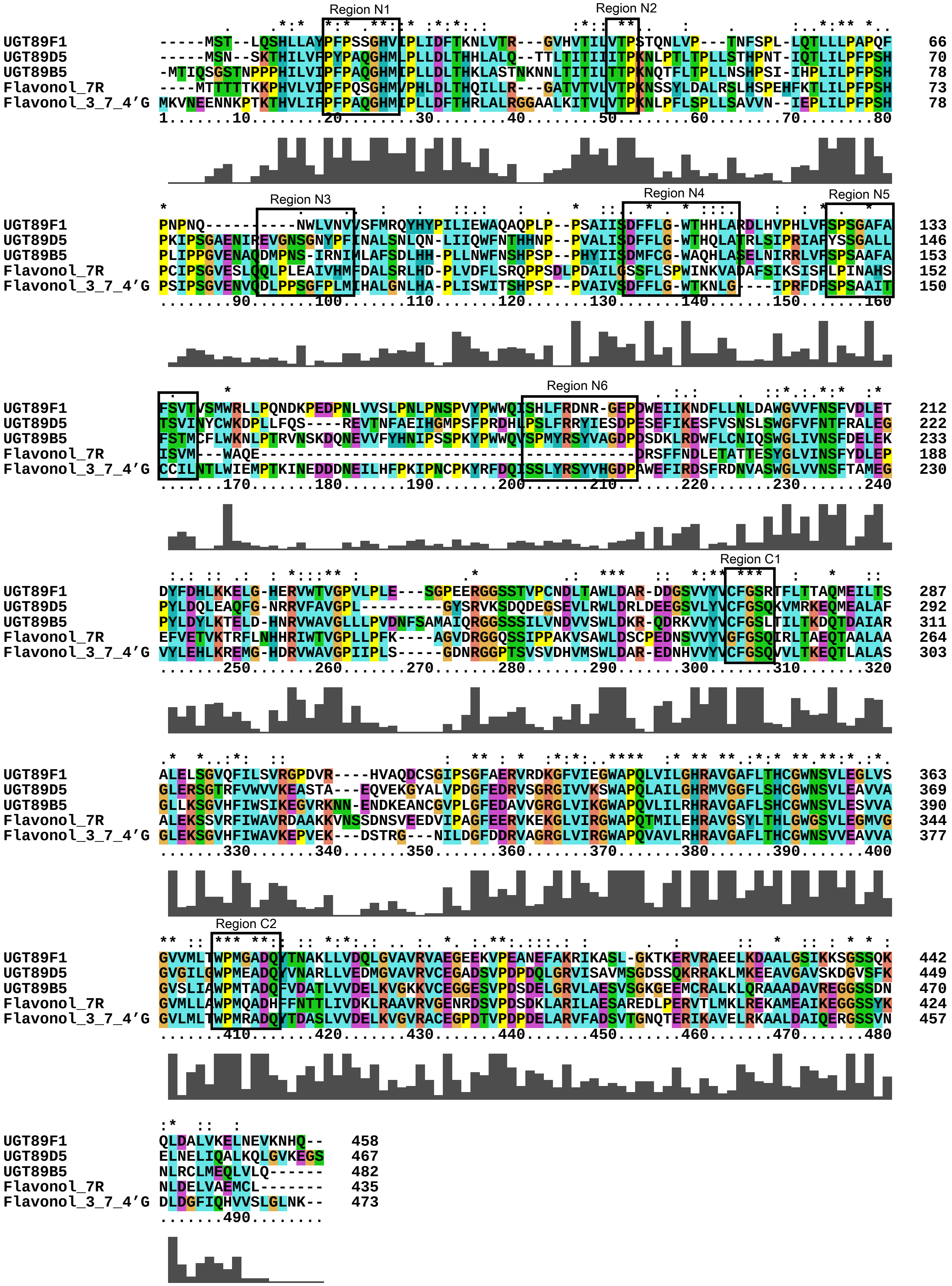
Region N1
Region N2
UGT89F1 -----MST--LQSHLLAYPFPSSGHVIPLIDFTKNLVTR---GVHVTILVTPSTQNLVP---TNFSPL-LQTLLLPAPQF 66
UGT89D5 -----MSN--SKTHILVFPYPAQGHMLPLLDLTHHLALQ--TTLTITIIITPKNLPTLTPLLSTHPNT-IQTLILPFPSH 70
UGT89B5 -MTIQSGSTNPPPHILVIPFPAQGHMIPLLDLTHKLASTNKNNLTITILTTPKNQTFLTPLLNSHPSI-IHPLILPFPSH 78
Flavonol_7R ----MTTTTTKKPHVLVIPFPQSGHMVPHLDLTHQILLR---GATVTVLVTPKNSSYLDALRSLHSPEHFKTLILPFPSH 73
Flavonol_3_7_4'G MKVNEENNKPTKTHVLIFPFPAQGHMIPLLDFTHRLALRGGAALKITVLVTPKNLPFLSPLLSAVVN--IEPLILPFPSH 78
Region N3
Region N4
Region N5
UGT89F1 PNPNQ-----------NWLVNVVSFMRQYHYPILIEWAQAQPLP--PSAIISDFFLG-WTHHLARDLHVPHLVFSPSGAFA 133
UGT89D5 PKIPSGAENIREVGNSGNYPFINALSNLQN-LIIQWFNTHHNP--PVALISDFFLG-WTHQLATRLSIPRIAFYSSGALL 146
UGT89B5 PLIPPGVENAQDMPNS-IRNIMLAFSDLHH-PLLNWFNSHPSP--PHYIISDMFCG-WAQHLASELNIRRLVFSPSAAFA 153
Flavonol_7R PCIPSGVESLQQLPLEAIVHMFDALSRLHD-PLVDFLSRQPPSDLPDAILGSSFLSPWINKVADAFSIKSISFLPINAHS 152
Flavonol_3_7_4'G PSIPSGVENVQDLPPSGFPLMIHALGNLHA-PLISWITSHPSP--PVAIVSDFFLG-WTKNLG----IPRFDFSPSAAIT 150
Region N6
UGT89F1 FSVTVSMWRLLPQNDKPEDPNLVVSLPNLPNSPVYPWWQISHLFRDNR-GEPDWEIIKNDFLLNLDAWGVVFNSFVDLET 212
UGT89D5 TSVINYCWKDPLLFQS----REVTNFAEIHGMPSFPRDHLPSLFRRYIESDPESEFIKESFVSNSLSWGFVFNTFRALEG 222
UGT89B5 FSTMCFLWKNLPTRVNSKDQNEVVFYHNIPSSPKYPWWQVSPMYRSYVAGDPDSDKLRDWFLCNIQSWGLIVNSFDELEK 233
Flavonol_7R ISVM---WAQE---DRSFFNDLETATTESYGLVINSFYDLEP 188
Flavonol_3_7_4'G CCILNTLWIEMPTKINEDDDNEILHFPKIPNCPKYRFDQISSLYRSYVHGDPAWEFIRDSFRDNVASWGLVVNSFTAMEG 230
Region C1
UGT89F1 DYFDHLKKELG-HERVWTVGPVLPLE---SGPEERGGSSTVPCNDLTAWLDAR-DDGSVVYVCFGSRTFLTTAQMEILTS 287
UGT89D5 PYLDQLEAQFG-NRRVFAVGPL---------GYSRVKSDQDEGSEVLRWLDRLDEEGSVLYVCFGSQKVMRKEQMEALAF 292
UGT89B5 PYLDYLKTELD-HNRVWAVGLLLPVDNFSAMAIQRGGSSSILVNDVVSWLDKR-QDRKVVYVCFGSLTILTKDQTDAIAR 311
Flavonol_7R EFVETVKTRFLNHHRIWTVGPLLPFK----AGVDRGGQSSIPPAKVSAWLDSCPEDNSVVYVGFGSQIRLTAEQTAALAA 264
Flavonol_3_7_4'G VYLEHLKREMG-HDRVWAVGPIIPLS-----GDNRGGPTSVSVDHVMSWLDAR-EDNHVVYVCFGSQVVLTKEQTLALAS 303
UGT89F1 ALELSGVQFILSVRGPDVR----HVAQDCSGIPSGFAERVRDKGFVIEGWAPQLVILGHRAVGAFLTHCGWNSVLEGLVS 363
UGT89D5 GLERSGTRFVWVVKEASTA---EQVEKGYALVPDGFEDRVSGRGIVVKSWAPQLAILGHRMVGGFLSHCGWNSVLEAVVA 369
UGT89B5 GLLKSGVHFIWSIKEGVRKNN-ENDKEANCGVPLGFEDAVVGRGLVIKGWAPQVLILRHRAVGAFLSHCGWNSVLESVVA 390
Flavonol_7R ALEKSSVRFIWAVRDAAKKVNSSDNSVEEDVIPAGFEERVKEKGLVIRGWAPQTMILEHRAVGSYLTHLGWGSVLEGMVG 344
Flavonol_3_7_4'G GLEKSGVHFIWAVKEPVEK---DSTRG---NILDGFDDRVAGRGLVIRGWAPQVAVLRHRAVGAFLTHCGWNSVVEAVVA 377
Region C2
UGT89F1 GVVMLTWPMGADQYTNAKLLVDQLGVAVRVAEGEEKVPEANEFAKRIKASL-GKTKERVRAEELKDAALGSIKKSGSSQK 442
UGT89D5 GVGILGWPMEADQYVNARLLVEDMGVAVRVCEGADSVPDPDQLGRVISAVMSGDSSQKRRAKLMKEEAVGAVSKDGVSFK 449
UGT89B5 GVSLIAWPMTADQFVDATLVVDELKVGKKVCEGGESVPDSDELGRVLAESVSGKGEEMCRALKLQRAAADAVREGGSSDN 470
Flavonol_7R GVMLLAWPMQADHFFNTTLIVDKLRAAVRVGENRDSVPDSDKLARILAESAREDLPERVTLMKLREKAMEAIKEGGSSYK 424
Flavonol_3_7_4'G GVLMLTWPMRADQYTDASLVVDELKVGVRACEGPDTVPDPDELARVFADSVTGNQTERIKAVELRKAALDAIQERGSSVN 457
UGT89F1 QLDALVKELNEVKNHQ-- 458
UGT89D5 ELNELIQALKQLGVKEGS 467
UGT89B5 NLRCLMEQLVLQ------ 482
Flavonol_7R NLDELVAEMCL------- 435
Flavonol_3_7_4'G DLDGFIQHVVSLGLNK-- 473

Group M

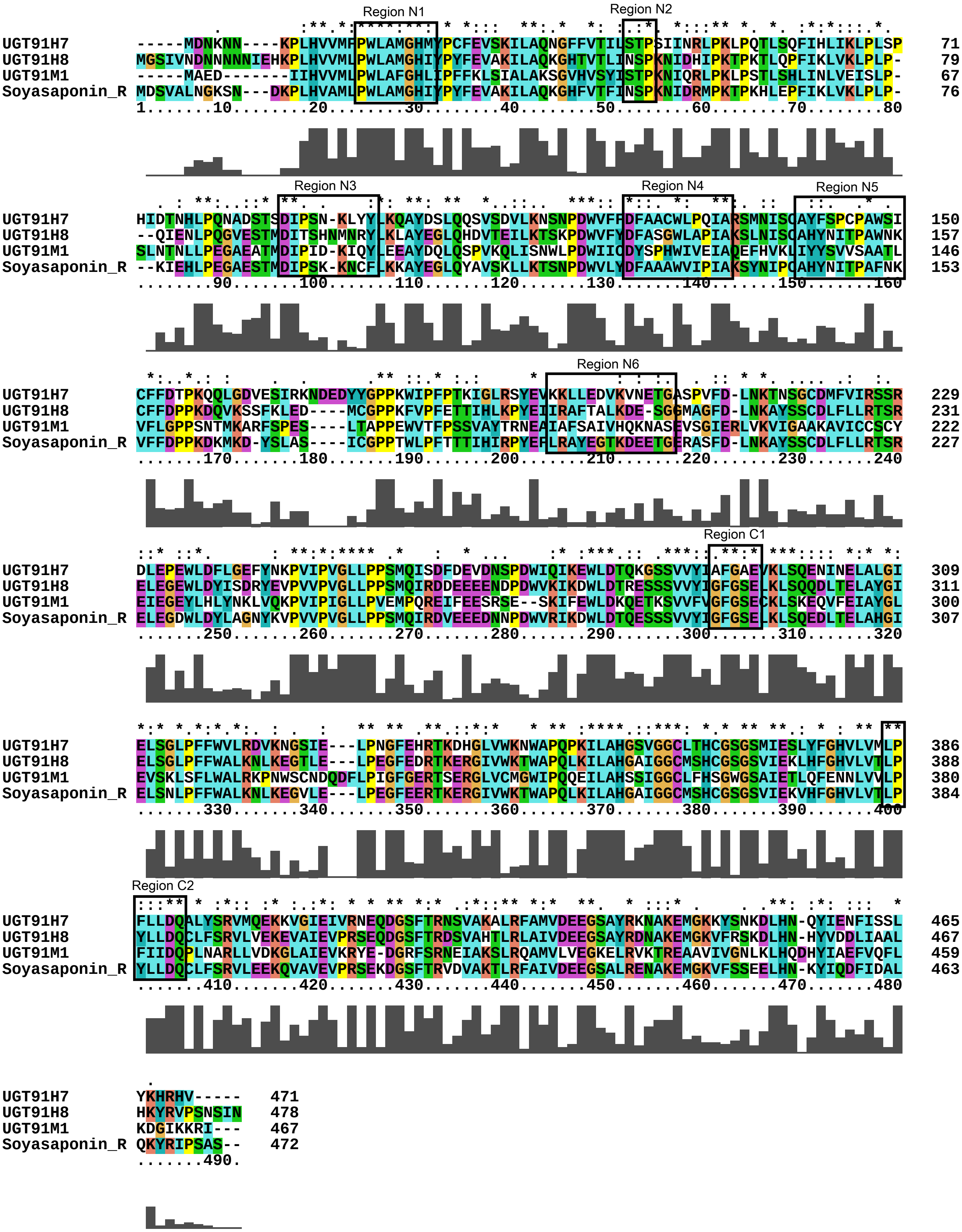
Region N1
Region N2
Region N3
Region N4
Region N5
Region N6
Region C1
Region C2
UGT91H7
UGT91H8
UGT91M1
Soyasaponin_R

Group N

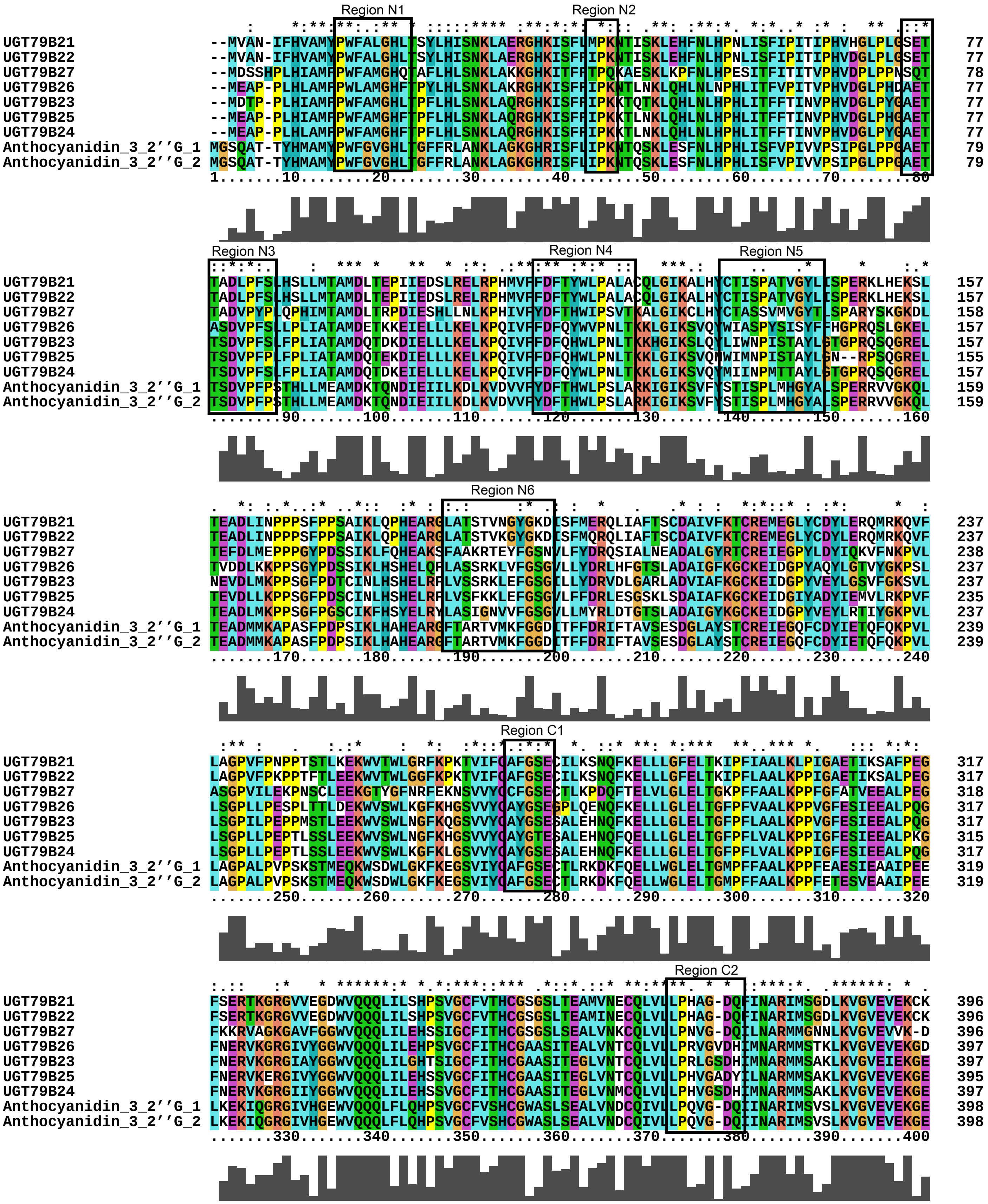
Region N1
Region N2
Region N3
Region N4
Region N5
Region N6
Region C1
Region C2
UGT79B21
UGT79B22
UGT79B27
UGT79B26
UGT79B23
UGT79B25
UGT79B24
Anthocyanidin_3_2’’G_1
Anthocyanidin_3_2’’G_2

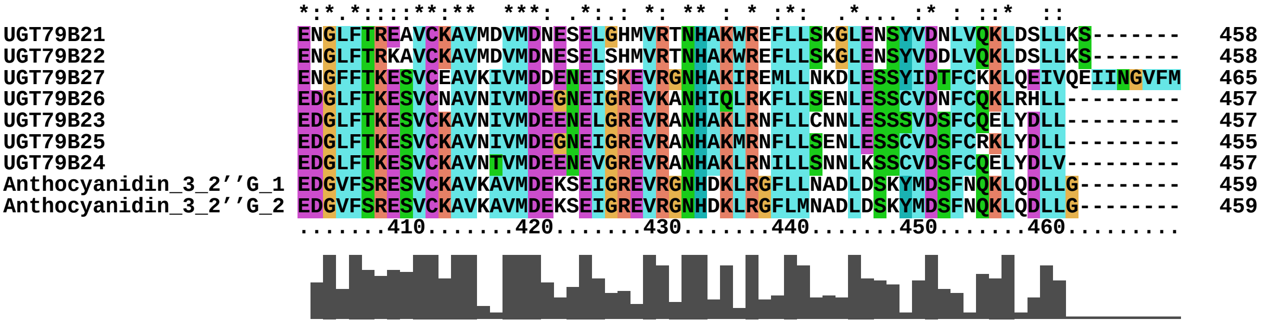
:.*::::**:** ***: .*:.: *: ** : * :*: .*... :* : ::* ::
UGT79B21 ENGLFTREAVCKAVMDVMDNESELGHMVRTNHAKWREFLLSKGLENSYVDNLVQKLDSLLKS------- 458
UGT79B22 ENGLFTRKAVCKAVMDVMDNESELSHMVRTNHAKWREFLLSKGLENSYVDDLVQKLDSLLKS------- 458
UGT79B27 ENGFFTKESVCEAVKIVMDDENEISKEVRGNHAKIREMLLNKDLESSYIDTFCKKLQEIVQEIINGVFM 465
UGT79B26 EDGLFTKESVCNAVNIVMDEGNEIGREVKANHIQLRKFLLSENLESSCVDNFCQKLRHLL--------- 457
UGT79B23 EDGLFTKESVCKAVNIVMDEENELGREVRANHAKLRNFLLCNNLESSSVDSFCQELYDLL--------- 457
UGT79B25 EDGLFTKESVCKAVNIVMDEGNEIGREVRANHAKMRNFLLSENLESSCVDSFCRKLYDLL--------- 455
UGT79B24 EDGLFTKESVCKAVNTVMDEENEVGREVRANHAKLRNILLSNNLKSSCVDSFCQELYDLV--------- 457
Anthocyanidin_3_2''G_1 EDGVFSRESVCKAVKAVMDEKSEIGREVRGNHDKLRGFLLNADLDSKYMDSFNQKLQDLLG-------- 459
Anthocyanidin_3_2''G_2 EDGVFSRESVCKAVKAVMDEKSEIGREVRGNHDKLRGFLMNADLDSKYMDSFNQKLQDLLG-------- 459
.......410.......420.......430.......440.......450.......460.........

Group O

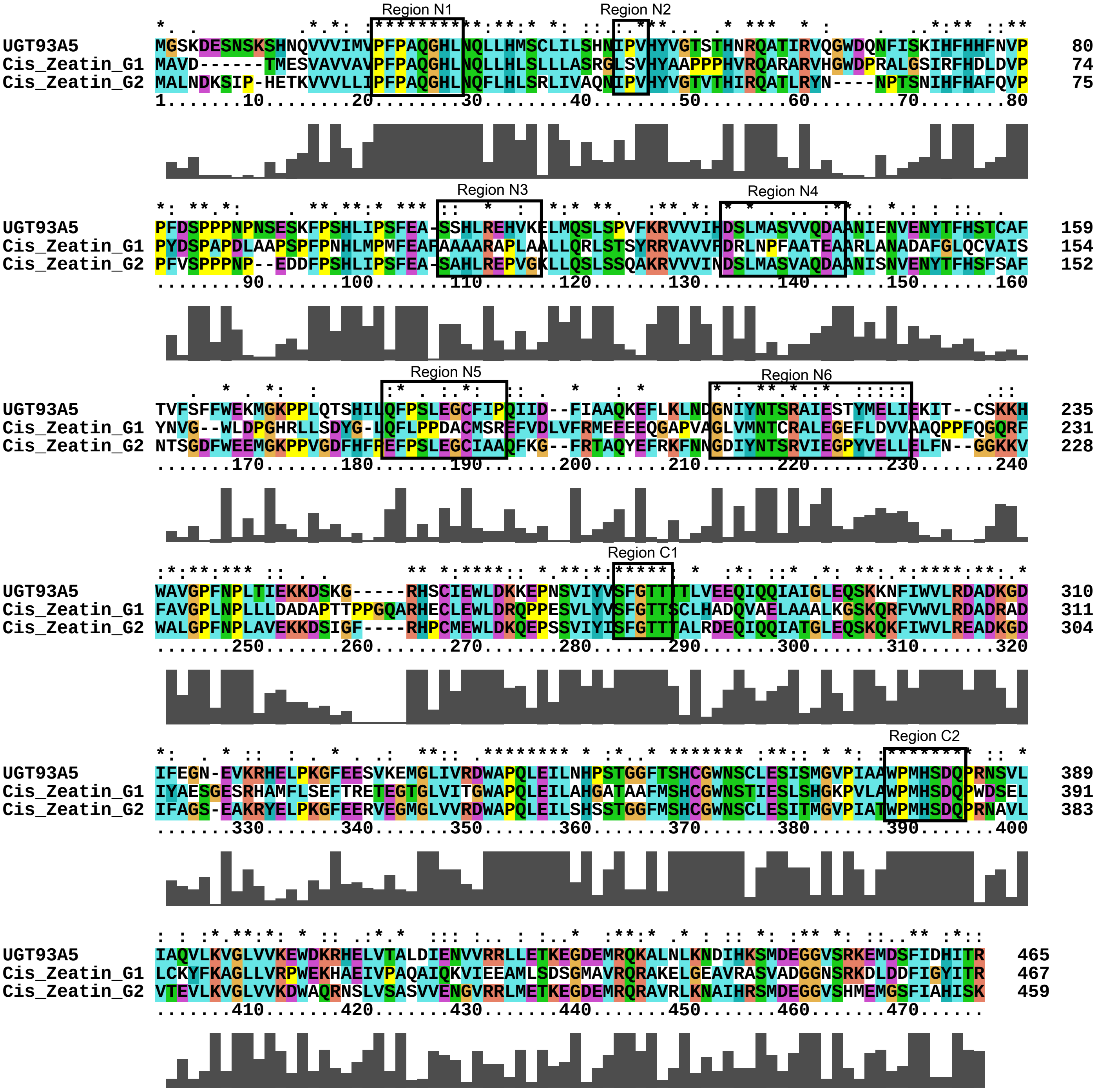
Region N1
Region N2
UGT93A5 MGSKDESNSKSHNQVVVIMVPFPAQGHLNQLLHMSCLILSHNIPVHYVGTSTHNRQATIRVQGWDQNFISKIHFHHFNVP 80
Cis_Zeatin_G1 MAVD------TMESVAVVAVPFPAQGHLNQLLHLSLLLASRGLSVHYAAPPPHVRQARARVHGWDPRALGSIRFHDLDVP 74
Cis_Zeatin_G2 MALNDKSIP-HETKVVVLLIPFPAQGHLNQFLHLSRLIVAQNIPVHYVGTVTHIRQATLRYN----NPTSNIHFHAFQVP 75
Region N3
Region N4
UGT93A5 PFDSPPPNPNSESKFPSHLIPSFEA-SSHLREHVKELMQSLSPVFKRVVVIHDSLMASVVQDAANIENVENYTFHSTCAF 159
Cis_Zeatin_G1 PYDSPAPDLAAPSPFPNHLMPMFEAFAAAARAPLAALLQRLSTSYRRVAVVFDRLNPFAATEAARLANADAFGLQCVAIS 154
Cis_Zeatin_G2 PFVSPPPNP--EDDFPSHLIPSFEA-SAHLREPVGKLLQSLSSQAKRVVVINDSLMASVAQDAANISNVENYTFHSFSAF 152
Region N5
Region N6
UGT93A5 TVFSFFWEKMGKPPLQTSHILQFPSLEGCFIPQIID--FIAAQKEFLKLNDGNIYNTSRAIESTYMELIEKIT--CSKKH 235
Cis_Zeatin_G1 YNVG--WLDPGHRLLSDYG-LQFLPPDACMSREFVDLVFRMEEEEQGAPVAGLVMNTCRALEGEFLDVVAAQPPFQGQRF 231
Cis_Zeatin_G2 NTSGDFWEEMGKPPVGDFHFPEFPSLEGCIAAQFKG--FRTAQYEFRKFNNGDIYNTSRVIEGPYVELLELFN--GGKKV 228
Region C1
UGT93A5 WAVGPFNPLTIEKKDSKG-----RHSCIEWLDKKEPNSVIYVSFGTTTTLVEEQIQQIAIGLEQSKKNFIWVLRDADKGD 310
Cis_Zeatin_G1 FAVGPLNPLLLDADAPTTPPGQARHECLEWLDRQPPESVLYVSFGTTSCLHADQVAELAAALKGSKQRFVWVLRDADRAD 311
Cis_Zeatin_G2 WALGPFNPLAVEKKDSIGF----RHPCMEWLDKQEPSSVIYISFGTTTALRDEQIQQIATGLEQSKQKFIWVLREADKGD 304
Region C2
UGT93A5 IFEGN-EVKRHELPKGFEESVKEMGLIVRDWAPQLEILNHPSTGGFTSHCGWNSCLESISMGVPIAAWPMHSDQPRNSVL 389
Cis_Zeatin_G1 IYAESGESRHAMFLSEFTRETEGTGLVITGWAPQLEILAHGATAAFMSHCGWNSTIESLSHGKPVLAWPMHSDQPWDSEL 391
Cis_Zeatin_G2 IFAGS-EAKRYELPKGFEERVEGMGLVVRDWAPQLEILSHSSTGGFMSHCGWNSCLESITMGVPIATWPMHSDQPRNAVL 383
UGT93A5 IAQVLKVGLVVKEWDKRHELVTALDIENVVRRLLETKEGDEMRQKALNLKNDIHKSMDEGGVSRKEMDSFIDHITR 465
Cis_Zeatin_G1 LCKYFKAGLLVRPWEKHAEIVPAQAIQKVIEEAMLSDSGMAVRQRAKELGEAVRASVADGGNSRKDLDDFIGYITR 467
Cis_Zeatin_G2 VTEVLKVGLVVKDWAQRNSLVSASVVENGVRRLMETKEGDEMRQRAVRLKNAIHRSMDEGGVSHMEMGSFIAHISK 459
