# Supplementary material for: Genome-Wide Identification and Tissue-Specific Expression Analysis of UDP-Glycosyltransferases Genes Confirm Their Abundance in Cicer arietinum (Chickpea) Genome
Source: PLoS One. 2014 Oct 7;9(10):e109715. doi: 10.1371/journal.pone.0109715 (PMC4188811; doi:10.1371/journal.pone.0109715)
Supplement: Figure S6 — Multiple sequence alignment of chickpea UGT protein sequences and their respective templates utilized for the homology modeling studies. (PDF) [file pone.0109715.s006.pdf]

Figure S6 Multiple sequence alignment of chickpea UGT protein sequences and their respective templates utilized for the homology modeling studies

Group A1

```
             *.   .:       ***:***** ***:***::*:::*  .*:..**** *: :* ::*   .  .  :   *** *:: **:
UGT78G2     MMPNLLKDETNVEKLHVAILAFPFGTHASPLLSLVRKIAGEVPKVTFSFFTTTASNATLFSPNNDCLP-NIKHYNVDDGL     80
2C1Z        -MSQTTTNP------HVAVLAFPFSTHAAPLLAVVRRLAAAAPHAVFSFFSTSQSNASIFHDSMHTMQCNIKSYDISDGV     80
3HBF        -MSTFKNEMNGNNLLHVAVLAFPFGTHAAPLLSLVKKIATEAPKVTFSFFCTTTTNDTLFSRSNEFLP-NIKYYNVHDGL     80
            1.......10........20........30........40........50........60........70........80
```

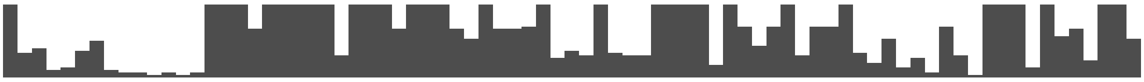

```
            *:*** :*.* * * ** :*  *.*:  :  ******: ::***:*** **.**:* ::  . *:*:*****::* **:*
UGT78G2     PEGYVPSGHPLEPIFLFIKAMPENFKSVMDEAVAETGKDITCLVTDAFYWFAADLAQQINAKWVPLWTAGPHALLTHIYT    160
2C1Z        PEGYVFAGRPQEDIELFTRAAPESFRQGMVMAVAETGRPVSCLVADAFIWFAADMAAEMGVAWLPFWTAGPNSLSTHVYI    160
3HBF        PKGYVSSGNPREPIFLFIKAMQENFKHVIDEAVAETGKNITCLVTDAFFWFGADLAEEMHAKWVPLWTAGPHSLLTHVYT    160
            ........90.......100.......110.......120.......130.......140.......150.......160
```

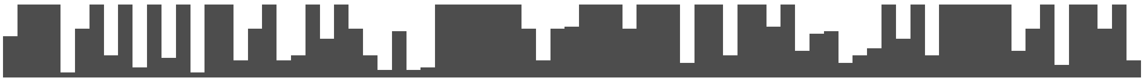

```
            * :*.  .  . ::. :.  :::.:**:.:::  ** **::  : :  *: ***:**  **:*.*: ****   :.   : *:*
UGT78G2     DLLRH--ACKQVHDVQN--VDLIPGFPELKVCDLPEGVID-DTDGPFATMLHKMGLELPRATAIAINSFATIHPLIENDL    240
2C1Z        DEIREKIGVSGIQGREDELLNFIPGMSKVRFRDLQEGIVFGNLNSLFSRMLHRMGQVLPKATAVFINSFEELDDSLTNDL    240
3HBF        DLIREKTGSKEVHDVKS--IDVLPGFPELKASDLPEGVIK-DIDVPFATMLHKMGLELPRANAVAINSFATIHPLIENEL    240
            .......170.......180.......190.......200.......210.......220.......230.......240
```

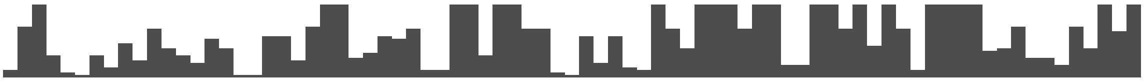

```
            :**:*  **:*** * **   :.:  ***:**.:::  ********:.  *** *: **:*:**  . .****::*...:
UGT78G2     NSKFKLLLNVGPFMLTTPQPLISDEQGCLEWLNQHKNFSVVYISFGSTIIPPPHELIALAESLEECGFPFIWALRGDPKE    320
2C1Z        KSKLKTYLNIGPFNLITPPPVVPNTTGCLQWLKERKPTSVVYISFGTVTTPPPAEVVALSEALEASRVPFIWSLRDKARV    320
3HBF        NSKFKLLLNVGPFNLTTPQRKVSDEHGCLEWLDQHENSSVVYISFGSVVTPPPHELTALAESLEECGFPFIWSFRGDPKE    320
            .......250.......260.......270.......280.......290.......300.......310.......320
```

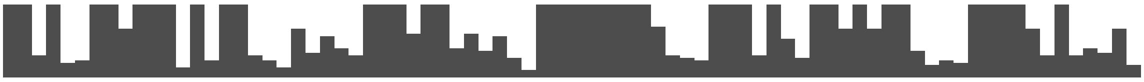

```
            :**:****:*    * :*.**** *:* *.:**. :**.****: *.:.****:*.******* **   : *.* **** ::
UGT78G2     KLPNGFLERTITKGKIVSWAPQMEILKHSSVGVCLTHCGWNSVLECIVGGVPMISRPFFGDQKLNARMLESVWEIGVGVD    400
2C1Z        HLPEGFLEKTRGYGMVVPWAPQAEVLAHEAVGAFVTHCGWNSLWESVAGGVPLICRPFFGDQRLNGRMVEDVLEIGVRIE    400
3HBF        KLPKGFLERTKTKGKIVAWAPQVEILKHSSVGVFLTHSGWNSVLECIVGGVPMISRPFFGDQGLNTILTESVLEIGVGVD    400
            .......330.......340.......350.......360.......370.......380.......390.......400
```

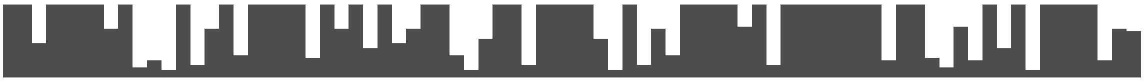

```
             **:**..  ..:.  :*.***  :*:::  *:*:* :**   :*:*: :* **:::*:.
UGT78G2     HGVLTKESTVKALKLIMSSEKGGVMRQNIVKLKESALKAVEQNGTSTKNFTTLIQIVTS----    463
2C1Z        GGVFTKSGLMSCFDQILSQEKGKKLRENLRALRETADRAVGPKGSSTENFITLVDLVSKPKDV    463
3HBF        NGVLTKESIKKALELTMSSEKGGIMRQKIVKLKESAFKAVEQNGTSAMDFTTLIQIVTS----    463
            .......410.......420.......430.......440.......450.......460...
```

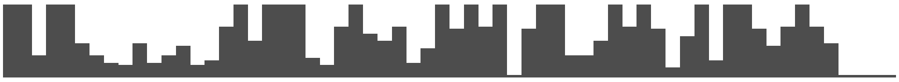

Group A2

```
        *:.:.::: *:*******:********:******* * :.**:***:****** **:*****:** *****:********
UGT71G2 MALNEMHNKKSELIFIPSPGIGHLASSLEFAKLLINTNTNLHITVLCIKFPGTPFSDSYIKTVLNSQPQIKLIDLPEVEQ   80
2ACW    MSMSDIN-KNSELIFIPAPGIGHLASALEFAKLLTNHDKNLYITVFCIKFPGMPFADSYIKSVLASQPQIQLIDLPEVEP   80
        1.......10........20........30........40........50........60........70........80
```

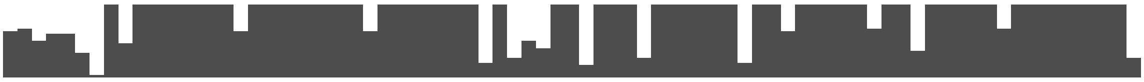

```
        **:***** *******:*******::*::** **:**********:****:***:*****:**************:***:
UGT71G2 PPHELLKSHEFYILTFMESLIPHVRSTLQTITSNQVVGLVLDFFCISMIDIGNELGIPSYMFLTSNVGFLSLMLSIKNRK  160
2ACW    PPQELLKSPEFYILTFLESLIPHVKATIKTILSNKVVGLVLDFFCVSMIDVGNEFGIPSYLFLTSNVGFLSLMLSLKNRQ  160
        ........90.......100.......110.......120.......130.......140.......150.......160
```

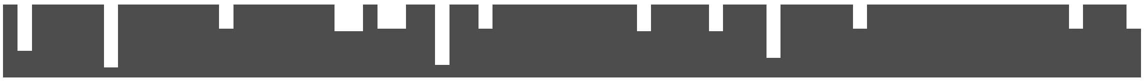

```
        **:*** :* *:*** ***:** ** ******.*******:*********.*************** :************
UGT71G2 IEDVFDYADPDNQLL-IPGFSNLVPFNVLPDAAFNKDGGYFAYYKLAERFNDTKGIIVNTFSDLEQYAIDALYDHDEKIP  240
2ACW    IEEVFDDSDRDHQLLNIPGISNQVPSNVLPDACFNKDGGYIAYYKLAERFRDTKGIIVNTFSDLEQSSIDALYDHDEKIP  240
        .......170.......180.......190.......200.......210.......220.......230.......240
```

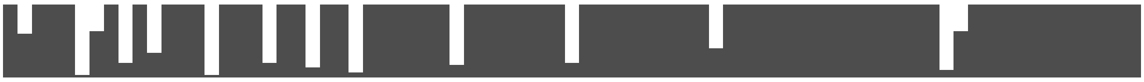

```
        ********:***.*.**:***********:*:**************** **  ** ********:*******:
UGT71G2 PIYAVGPLIDLKSQTNPNLDQAQHDLILKFLNEQPDKSVVFLCFGSMG-SFVLSQTREIALGLKKSGVRFLWAMRSPPTS  320
2ACW    PIYAVGPLLDLKGQPNPKLDQAQHDLILKWLDEQPDKSVVFLCFGSMGVSFGPSQIREIALGLKHSGVRFLWSN------  320
        .......250.......260.......270.......280.......290.......300.......310.......320
```

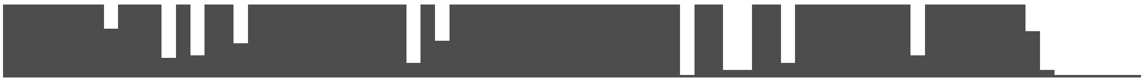

```
        . * * :*********::*************************************:*********************:***:
UGT71G2 NNEGKSLPEGFLEWMNMEGKGMICGWAPQVEVLAHKAIGGFVSHCGWNSILESLWFGVPILTWPIYAEQQLNAFRMVKEF  400
2ACW    SAEKKVFPEGFLEWMELEGKGMICGWAPQVEVLAHKAIGGFVSHCGWNSILESMWFGVPILTWPIYAEQQLNAFRLVKEW  400
        .......330.......340.......350.......360.......370.......380.......390.......400
```

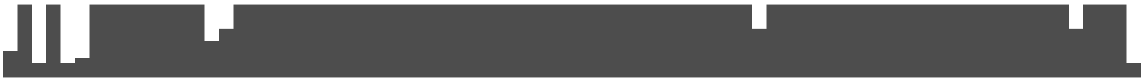

```
        *:*: **:***:***** *********.**:**.:*:**:***   :**********:*******::: ***
UGT71G2 GLGVELRMDYRNGSDVVLAEEIEKGLKHLMEKDNVVQKKLQEM---ARNAVVDGGSSFISVGKLIQNMIGSN  472
2ACW    GVGLGLRVDYRKGSDVVAAEEIEKGLKDLMDKDSIVHKKVQEMKEMSRNAVVDGGSSLISVGKLIDDITGSN  472
        .......410.......420.......430.......440.......450.......460.......470..
```

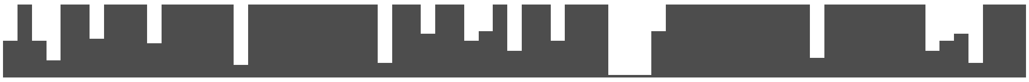

Group B

```
           *********:*.**:*******:****:***.*:*   :*********.***********:.*******.:*****: ::
```

```
           * **:*****.*: ***.*******:*******************************::::********:*******:**
UGT85H3    MRNFTNRKPHAVLTPYPAQGHINPLLKLAKLLHLRGFHITFVNTEYNHKRLLKSRGPNSLNGFTDFNFETIPDGLTPIEG    80
2PQ6       MGNFANRKPHVVMIPYPVQGHINPLFKLAKLLHLRGFHITFVNTEYNHKRLLKSRGPKAFDGFTDFNFESIPDGLTPMEG    80
           1.......10........20........30........40........50........60........70........80
```

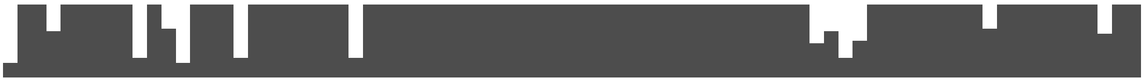

```
           *********:*.**:*******:****:***.*:*   :*********.***********:.*******.:*****: ::
UGT85H3    DGDVSQDVPSLSQSIRKNFLKPFCELLARLNDSANDALIPPVTCLVSDSCMSFTIQAAEELSLPNVLYFPASACSLMCIL   160
2PQ6       DGDVSQDVPTLCQSVRKNFLKPYCELLTRLNHSTN---VPPVTCLVSDCCMSFTIQAAEEFELPNVLYFSSSACSLLNVM   160
           ........90.......100.......110.......120.......130.......140.......150.......160
```

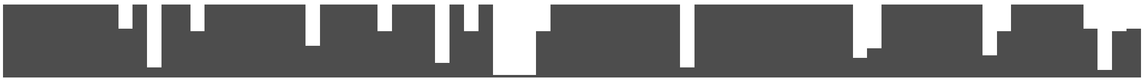

```
           *******:*: *:****** ** **:**:************** **************:**:*****:::**:*******
UGT85H3    HFRSFVEKGLTPLKDESYLRNGFLEAKVEWIPGLKNFRLKDIVAFIRTTNPNDIMLEFLIEMADRVNRESTIVLNTFNEL   240
2PQ6       HFRSFVERGIIPFKDESYLTNGCLETKVDWIPGLKNFRLKDIVDFIRTTNPNDIMLEFFIEVADRVNKDTTILLNTFNEL   240
           .......170.......180.......190.......200.......210.......220.......230.......240
```

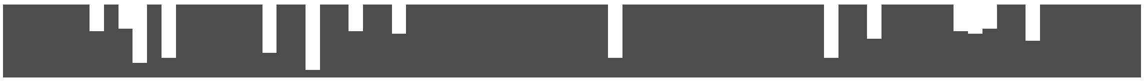

```
           *****:**** :**:**********:**** * * **..*******:**:******* *:******* **** **:****
UGT85H3    ESDVIDALSSMFPSLYPIGPLPSLLNQTPQNH-LASLGCNLWKEDTKCLEWLESKEPRSIVYVNFGSITVMTHEQVLEFA   320
2PQ6       ESDVINALSSTIPSIYPIGPLPSLLKQTPQIHQLDSLDSNLWKEDTECLDWLESKEPGSVVYVNFGSTTVMTPEQLLEFA   320
           .......250.......260.......270.......280.......290.......300.......310.......320
```

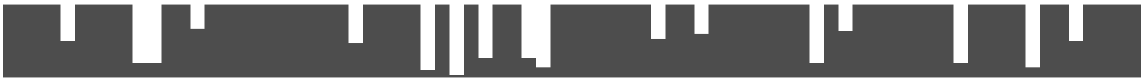

```
           *****.**.***************::****.** :***********:*********************************
UGT85H3    WGLANSKKPFLWIIRPDLVIGGSVVLSSEFVNETSDRGLIASWCPQEKVLNHPSIGGFLTHCGWNSTTESICAGVPMLCW   400
2PQ6       WGLANCKKSFLWIIRPDLVIGGSVIFSSEFTNEIADRGLIASWCPQDKVLNHPSIGGFLTHCGWNSTTESICAGVPMLCW   400
           .......330.......340.......350.......360.......370.......380.......390.......400
```

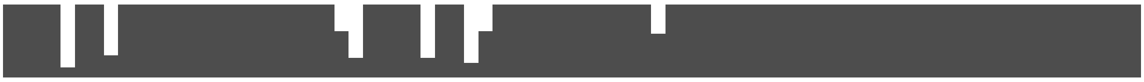

```
           *.*.****:***********:**********: **:**:*.*:*****::******:** *:** ********:***::*
UGT85H3    PVFGDQPTNCRFICNEWEIGLEIDTNVKREEVEKLVNELIVGEKGKKMREKAMELKNKAMEDTRVGGCSYMNLDKVINEV   480
2PQ6       PFFADQPTDCRFICNEWEIGMEIDTNVKREELAKLINEVIAGDKGKKMKQKAMELKKKAEENTRPGGCSYMNLNKVIKDV   480
           .......410.......420.......430.......440.......450.......460.......470.......480
```

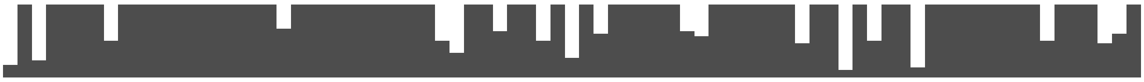

```
           ***:*
UGT85H3    LLKHN    485
2PQ6       LLKQN    485
           .....
```

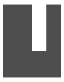

Group G

```
                 .* * **::***********::*****::  *.: ***:*. *..***** :**:***.*** :*****.::**.*.*
UGT72B18    -MAPKLPMVAMLPSPGMGHLIPMIEFAKRIILHHNIHVTFIIPTEASPSKAQISVLQSLPKSISHIFLPPVSFADLPSNT   80
2VCH        MEESKTPHVAIIPSPGMGHLIPLVEFAKRLVHLHGLTVTFVIAGEGPPSKAQRTVLDSLPSSISSVFLPPVDLTDLSSST   80
            1.......10........20........30........40........50........60........70........80
```

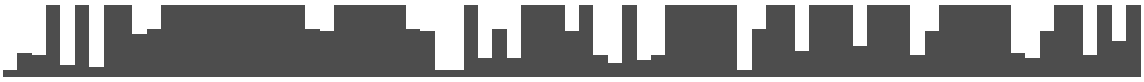

```
            :**: ***** ** *.**:.* *: :.  * **::******:********::* *:***:** .*****:*.:**: * *
UGT72B18    KIETLISLTVLRSLPSLRQTFRSLSDSYTL-TAVIVDLFGTEAFDVAVEFNLPRYVFYPSTAMALSFFLYLSRLDQEVHC  160
2VCH        RIESRISLTVTRSNPELRKVFDSFVEGGRLPTALVVDLFGTDAFDVAVEFHVPPYIFYPTTANVLSFFLHLPKLDETVSC  160
            ........90.......100.......110.......120.......130.......140.......150.......160
```

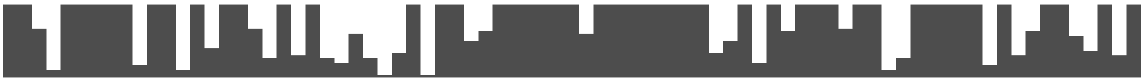

```
            *:.*****: :***:*: ***:***.****:*****:*:*::**:**:**: *:*.****..** **:   .********
UGT72B18    EYNELTEPVHIPGCIPIHGKDLLDPVQDRKNDAYKWVLRNAERYREADGIIENSFVELEPGPIKELQKEEPGKPPVYPVG  240
2VCH        EFRELTEPLMLPGCVPVAGKDFLDPAQDRKDDAYKWLLHNTKRYKEAEGILVNTFFELEPNAIKALQEPGLDKPPVYPVG  240
            .......170.......180.......190.......200.......210.......220.......230.......240
```

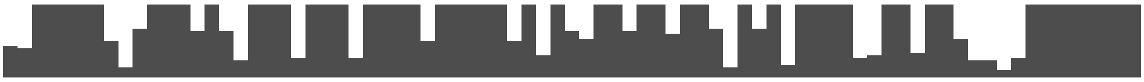

```
            ****  *: *:  .:********.** ****:**********.:*: ******  ********:***.. :**:***.:.
UGT72B18    PLVN--KDVAQTGSNSECLKWLDGQPHGSVLFVSFGSGGTLTSNQIIELALGLEMSEQRFLWVVRSPNDKVANASYFSAD  320
2VCH        PLVNIGKQEAKQTEESECLKWLDNQPLGSVLYVSFGSGGTLTCEQLNELALGLADSEQRFLWVIRSPSG-IANSSYFDSH  320
            .......250.......260.......270.......280.......290.......300.......310.......320
```

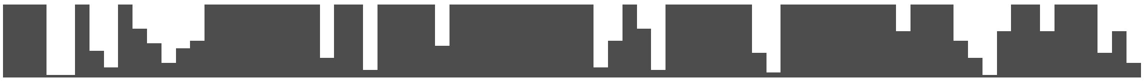

```
            :*:**: *** ******:***:*:.  ****.***** ************* *****..:**:.***********:*:**
UGT72B18    TQADPFDFLPKGFLERTNKRGLVVSSWAPQPQVLAHGSTGGFLTHCGWNSILESVVNAVPLVVWPLYAEQKMNAVILTED  400
2VCH        SQTDPLTFLPPGFLERTKKRGFVIPFWAPQAQVLAHPSTGGFLTHCGWNSTLESVVSGIPLIAWPLYAEQKMNAVLLSED  400
            .......330.......340.......350.......360.......370.......380.......390.......400
```

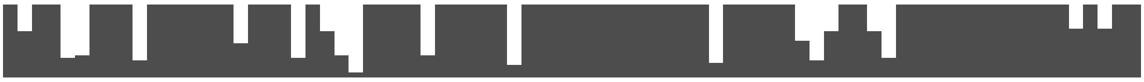

```
            ::.****..*::***.*:*:* *** ******** :* :**:**:** :.* ::****: :* :****. .:.  :
UGT72B18    VKVALRPNVGENGLVERQEIASVVKCLMEGEEGKKLRYQMKDLKDAAVKTLGENGTSTNYISKLALKWSNKSNVTN----  480
2VCH        IRAALRPRAGDDGLVRREEVARVVKGLMEGEEGKGVRNKMKELKEAACRVLKDDGTSTKALSLVALKWKAHKKELEQNGN  480
            .......410.......420.......430.......440.......450.......460.......470.......480
```

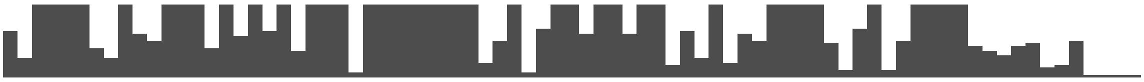

```
UGT72B18    -   481
2VCH        H   481
            .
```

Group G

```
             :**.*:*:*****:.**:*::**:****  *  : :* :*.. *.*.::.:: *:::*..*.*:****::  **...
UGT72X1    --MAKTTHIAVIPSPGFSHLVPILEFTKRLVTNHPNFHITCIIPSLGSPPNSSKSYLQTIPPNINSIFLPPINKHDLPQG    80
2VCH       MEESKTPHVAIIPSPGMGHLIPLVEFAKRLVHLHG-LTVTFVIAGEGPPSKAQRTVLDSLPSSISSVFLPPVDLTDLSSS    80
           1.......10........20........30........40........50........60........70........80
```

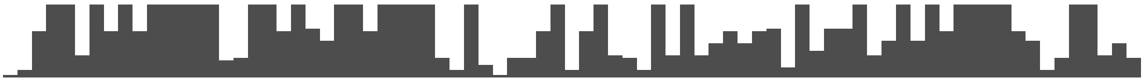

```
           :     *.**** * *.::::.:.*:  .  .  * .**:.* *. :*:*.* **:   .*:::*::*  **: *** **** **
UGT72X1    AYPGVIIQLTVTLSLPSIHQALKSLNSKAPL-VALIADSFAFEALDFAKEFNSLSYLYFPSSAMNLSLSLHLVKLDEEVS   160
2VCH       TRIESRISLTVTRSNPELRKVFDSFVEGGRLPTALVVDLFGTDAFDVAVEFHVPPYIFYPTTANVLSFFLHLPKLDETVS   160
           ........90.......100.......110.......120.......130.......140.......150.......160
```

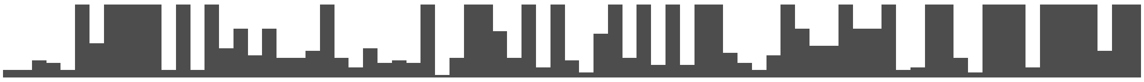

```
           **:::* **: * ****: *:*:  *:: * .:**** :*:.:*     :*** *:*:***..*****:* *  .    * .
UGT72X1    CEYKDLQEPIKLQGCVPIHGRDLPAPTKMRSNDAYKKFLQRAKSMYFFDGILCNSFLELESGAIKALEEKGHHENGKKIS   240
2VCH       CEFRELTEPLMLPGCVPVAGKDFLDPAQDRKDDAYKWLLHNTKRYKEAEGILVNTFFELEPNAIKALQEPGLD----KPP   240
           .......170.......180.......190.......200.......210.......220.......230.......240
```

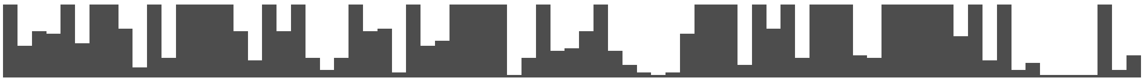

```
           ::****:.: *..  ::::.:* ******.*** .*************: .*:**** **  *:******:*:**. ..:.
UGT72X1    IFPVGPITQKGSSASSNDVDEFECLKWLKNQPQNSVLYVSFGSGGTLSLRQINELAYGLELSDQRFLWVLRAPSDSVSAD   320
2VCH       VYPVGPLVNIGKQE-AKQTEESECLKWLDNQPLGSVLYVSFGSGGTLTCEQLNELALGLADSEQRFLWVIRSPSGIANSS   320
           .......250.......260.......270.......280.......290.......300.......310.......320
```

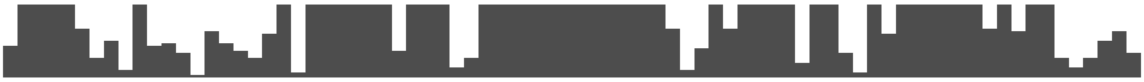

```
           **:. .  .***.*** *******::*:*:.  ****.:** :.*.****:****** ***:  .*:*::****:*** ****
UGT72X1    YFEDAN-VDPLKFLPKGFLERTKEKGLVLASWAPQVEVLKQSSVGGFLSHCGWNSILESIQEGVPIVAWPLFAEQAMNAV   400
2VCH       YFDSHSQTDPLTFLPPGFLERTKKRGFVIPFWAPQAQVLAHPSTGGFLTHCGWNSTLESVVSGIPLIAWPLYAEQKMNAV   400
           .......330.......340.......350.......360.......370.......380.......390.......400
```

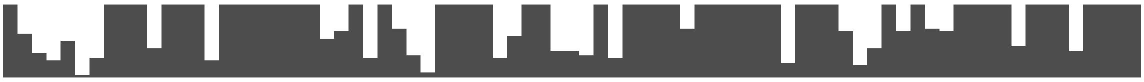

```
           :**:.:..*:* :  ** :*.:*::*:*** *********:*::** **:.*.:.*****:* ::** :* :*: .
UGT72X1    MLSDGLEVAIRLKFEDDEIVEKEKIAKVVKCLMEGEEGKGIRERMKVLKDGAAKALKDDGSSIQTLSYLANQWENFGGI-   480
2VCH       LLSEDIRAALRPRAGDDGLVRREEVARVVKGLMEGEEGKGVRNKMKELKEAACRVLKDDGTSTKALSLVALKWKAHKKEL   480
           .......410.......420.......430.......440.......450.......460.......470.......480
```

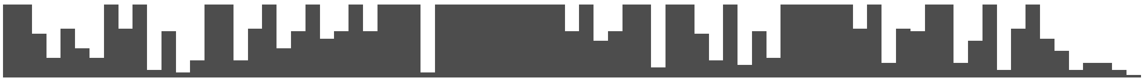

```
UGT72X1    ------   486
2VCH       EQNGNH   486
           ......
```
