# Supplementary material for: Genome-Wide Identification and Tissue-Specific Expression Analysis of UDP-Glycosyltransferases Genes Confirm Their Abundance in Cicer arietinum (Chickpea) Genome
Source: PLoS One. 2014 Oct 7;9(10):e109715. doi: 10.1371/journal.pone.0109715 (PMC4188811; doi:10.1371/journal.pone.0109715)
Supplement: Table S6 — Statistics of Blast results. (DOC) [file pone.0109715.s014.doc]

Table S6 Statistics of Blast results

| **Serial number** | **UGT** | **Group** | **Template** | **Identity (%)** | **Resolution (Å)** |
| --- | --- | --- | --- | --- | --- |
| 1 | UGT78G2 | A1 | 3HBF  2C1Z | 76  47 | 2.10  1.90 |
| 2 | UGT71G2 | A2 | 2ACV | 79 | 2 |
| 3 | UGT85H3 | B | 2PQ6 | 79 | 2.10 |
| 4 | UGT72B18 | G | 2VCH | 62 | 1.45 |
| 5 | UGT72X1 | G | 2VCH | 48 | 1.45 |
