# Supplementary material for: Genome-Wide Identification and Tissue-Specific Expression Analysis of UDP-Glycosyltransferases Genes Confirm Their Abundance in Cicer arietinum (Chickpea) Genome
Source: PLoS One. 2014 Oct 7;9(10):e109715. doi: 10.1371/journal.pone.0109715 (PMC4188811; doi:10.1371/journal.pone.0109715)
Supplement: Table S7 — Structure evaluation statistics of generated homology models of Ca UGTs protein sequences. (DOC) [file pone.0109715.s015.doc]

**Table S7 Structure evaluation statistics of generated homology models of *Ca*UGTs protein sequences.**

| **Serial number** | **UGT** | **Group** | **Ramachandran plot** | **Verify3D score** | **ERRAT value** |
| --- | --- | --- | --- | --- | --- |
| 1 | UGT78G2 | A1 | 92.3*, 0** | 96.48 | 85.2 |
| 2 | UGT71G2 | A2 | 92.4, 0.7 | 96.58 | 76 |
| 3 | UGT85H3 | B | 90.1, 0.5 | 95.46 | 70.33 |
| 4 | UGT72B18 | G | 91.7, 0.5 | 98.52 | 79.52 |
| 5 | UGT72X1 | G | 99, 1 | 98.93 | 84.4 |

*** Represent percent of the total residue present in the allowed region of Ramachandran plot. ** Represent percent of the total residue present in the disallowed region of Ramachandran plot. The values in the fifth and sixth column represent the Verify3D score and ERRAT score of CaUGT models.**
